# Direct Semi-Synthesis of the Anticancer Lead-Drug Protoapigenone from Apigenin, and Synthesis of Further New Cytotoxic Protoflavone Derivatives

**DOI:** 10.1371/journal.pone.0023922

**Published:** 2011-08-30

**Authors:** Attila Hunyadi, Da-Wei Chuang, Balazs Danko, Michael Y. Chiang, Chia-Lin Lee, Hui-Chun Wang, Chin-Chung Wu, Fang-Rong Chang, Yang-Chang Wu

**Affiliations:** 1 Institute of Pharmacognosy, University of Szeged, Szeged, Hungary; 2 Graduate Institute of Natural Products, Kaohsiung Medical University, Kaohsiung, Taiwan; 3 Department of Chemistry, National Sun Yat-Sen University, Kaohsiung, Taiwan; 4 Graduate Institute of Integrated Medicine, College of Chinese Medicine, China Medical University, Taichung, Taiwan; 5 Cancer Center, Kaohsiung Medical University Hospital, Kaohsiung, Taiwan; 6 Research and Development Center of Chinese Herbal Medicines and New Drugs, College of Pharmacy, Kaohsiung Medical University, Kaohsiung, Taiwan; 7 Natural Medicinal Products Research Center, China Medical University Hospital, Taichung, Taiwan; University of Sydney, Australia

## Abstract

Protoapigenone, a natural flavonoid possessing an unusual *p*-quinol moiety on its B-ring, is a novel prospective anticancer agent with low toxicity that is currently in development. The first economical, one-step synthesis of protoapigenone from apigenin is described on up to gram scale. 13 new 1′-*O*-alkylflavone analogs were also synthesized, either from apigenin or β-naphthoflavone. The *in vitro* cytotoxic activity of each compound was tested on six human cancer cell lines (HepG2, Hep3B, Ca9-22, A549, MCF-7 and MDA-MB-231). In the case of 1′-*O*-alkyl-protoapigenone derivatives, structure-activity relationships were found depending on the side-chain, and protoapigenone 1′-*O*-butyl ether was found to exert significantly stronger activity against three of the cell lines (Hep3B, MCF-7 and MDA-MB-231) than its non-substituted analog, protoapigenone itself. In contrast to this, all β-naphthoflavone derivatives bearing the same pharmacophore on their B-ring showed decreased cytotoxic activities when substituted with an *O*-alkyl side-chain at position 1′, comparing to that of the non-substituted compound.

## Introduction

Flavonoids are among the most widespread secondary plant metabolites and play a significant role in the prevention of several chronic diseases, including cancer [1]–[3]. Due to their numerous anticancer functions, including antiproliferative, cell cycle arresting and pro-apoptotic effects, certain members of this chemical group are believed to have potential not only in chemoprevention but also as future chemotherapy agents for treating cancer [4], [5].

**Figure 1:**
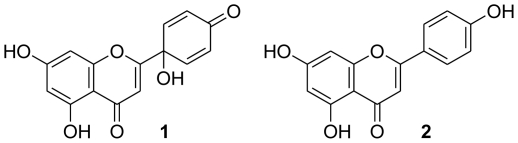
Structures of protoapigenone (1) and apigenin (2).

Protoapigenone (**1**) was first isolated by our group from the Formosan fern *Thelypteris torresiana* [6]. This compound has a close structural and likely biosynthetic relationship with apigenin (**2**), a common 4′-hydroxy-flavone (Figure 1) that is abundantly present in fruits and vegetables and is considered a particularly potent cancer chemopreventive flavonoid, according to a growing body of data [7], [8].

*In vitro*, **1** exhibits significant antitumor activities toward HepG2, Hep3B, MCF-7, A549 and MDA-MB-231 tumor cell lines (IC_50_ 0.27–3.88 µg/mL). The compound was also found to induce apoptosis in human prostate cancer cells [9] and to selectively inhibit ovarian cancer cell growth both *in vitro* and *in vivo* in nude mice while causing no major side effects in the latter case [10]. In a recent study, **1** was shown to exert dose-dependent DNA damage, apoptosis, and G2/M arrest at micromolar concentrations in a lung cancer cell line (H1299) [11]. Based on its potent pro-apoptotic activity and apparent low toxicity, **1** was chosen by the National Science Council of Taiwan as the lead compound for the development of a new class of anticancer drugs. Presently, *in vivo* toxicology studies are being performed as part of the preclinical phase, which have created a need for an economical way to drastically increase the production of **1**.

**Figure 2:**
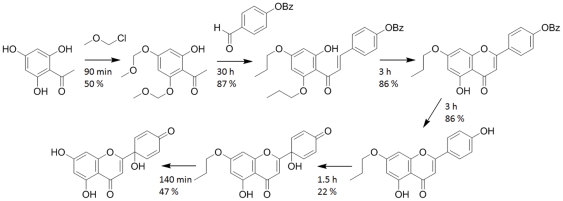
The total-synthesis of protoapigenone as reported previously [**12**]. Reaction times and isolated yields are shown for each reaction step. Purification of each intermediate product was performed by column chromatography on silica.

In 2007, a six-step total synthesis of **1** was reported by our group [12]. After difficulties in carrying out the direct semisynthesis of **1** from **2**, we used methoxymethyl-diprotected trihydroxyacetophenones and 4-benzyloxybenzaldehyde as starting materials to prevent the A-ring of **2** from oxidation, and we applied a hypervalent iodine reaction to the 7-protected apigenin skeleton in the second-to-last step to obtain the *p*-quinol moiety on the B-ring. Finally, **1** was successfully obtained after the removal of the protecting group. To carry out the key oxidation step that formed the pharmacophore on the B-ring, we applied 2 equivalent (eq.) of [bis(trifluoroacetoxy)iodo]benzene (commonly referred to as PIFA) in the presence of 0.2 eq. of TEMPO, a commercially available free radical catalyst. Although in some related oxidative dearomatization reactions phenyliodine diacetate (PIDA) was found to give higher yield of product as compared to when PIFA was used [13], PIDA was less effective in our case. This was also in accordance with the results of Wells et al. [14], who found the use of PIFA and TEMPO advantegous over PIDA. Based on these, PIFA was kept as the only oxidizing agent when the methodology reported here was developed. The reaction was originally performed by stirring at 25°C for 90 min in acetonitrile∶water (9∶1, v/v) at a concentration of 18 mg/mL of 7-protected **2**, and, after deprotection, **1** was successfully obtained. Figure 2 shows an outline of this procedure, including reaction times and isolated yields of the reaction steps.

The procedure resulted in the synthesis of protoapigenone as well as several of its derivatives which allowed SAR data to be gathered. However, due to the long reaction times and the slow chromatographic purifications, the procedure took approximately one to two months to complete, and resulted in only a 3.3% overall yield of **1**. Because this means of production was unable to satisfy the high material needs for the extensive preclinical pharmacological investigation, the improvement of the synthetic route has become an urgent need and a primary objective of our research program.

## Results and Discussion

### Synthesis

Our attempt to improve the yield of the original method began with the optimization of the oxidation of 7-MOM-apigenin but led to the surprising observation that with appropriate reaction conditions, **1** could be directly synthesized from **2** without the need for protecting groups. Optimization of the direct semisynthesis provided the following insights:

- By decreasing the concentration of the starting material to 1 mg/mL, the yield could be improved significantly; further decreases of concentration did not provide further increases in the yield.
- TEMPO significantly decreases the yield, and the formation of **1** may be prevented completely by increasing the amount of TEMPO used. Thus, it should be omitted from the reaction.
- The reaction is highly dependent on the solvent used. For success, the majority of the solvent should be a non-nucleophilic polar solvent, preferably acetonitrile. When using acetone∶water (9∶1, v/v) and THF∶water (9∶1, v/v), the reaction failed. EtOAc saturated with water gave traces of **1**, while 1,1,1,3,3,3-hexafluoro-isopropanol∶water (9∶1, v/v) resulted in about half the yield of that obtained using the ACN∶water mixture.
- Low energy microwave heating (70°C, 1 min, 500 W) can increase the yield by more than two-fold, compared to when the reaction is performed at room temperature.
- The TFA (trifluoroacetic acid) derived from the PIFA in the reaction significantly decreases the pH. Compound **1** does not suffer quick decomposition, but it is slightly sensitive to acidic environments, so it is recommended that it be purified as soon as possible. On the other hand, neutralizing the TFA is not favorable, as the remaining ionizable phenolic hydroxyl groups may lead to a less complete separation at the purification process.

**Figure 3:**
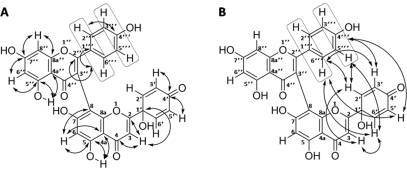
Structure, numbering and key HMBC (A) and NOESY (B) correlations of 1″.

As mentioned above, we performed the reaction via a quick, microwave-assisted oxidation of **2** (1 mg/mL in acetonitrile∶water 9∶1, v/v) with 2 eq. of PIFA, which resulted in a 31% isolated yield for 100 mg of starting material. This was a great improvement over the previous method [12]. The purification consisted of a solid phase extraction followed by gel chromatography on Sephadex LH-20, which also resulted in the isolation of two side-products, **1′** and **1″**. Based on its NMR spectra and chromatographic properties in comparison with available testcompound and literature data, **1′** was identified as luteolin, a common flavone and an expected side-product of the performed reaction [15]. By means of ESI-MS and HRMS data, **1″** was found to have a molecular mass of 554 and a constitution of C_30_H_18_O_11_ corresponding to an apigenin-protoapigenone dimer. The ^1^H NMR spectrum suggested, that a ring corresponding to the B-ring of apigenin is present (2H at both 7.38 and 6.77 ppm, *J* = 8.8 Hz). Interestingly, four separate hydrogens were shown as doublet-doublets with the larger coupling constant of 10.0 Hz. This coupling would be characteristic to the two doublets, two hydrogens each, of the B-ring of protoapigenone. Based on the ^1^H-^1^H COSY spectrum, these four hydrogens were also on the same ring, suggesting a cyclohexadienone ring, as in protoapigenone, also to be present. Differences between the pairs of corresponding protons on this ring are suggested to arise from the effect of the close aromatic B-ring of the apigenin part, which was also shown by NOESY correlations between hydrogens of these two rings. The number of non-OH hydrogens and the presence of only two meta-coupling doublets accompanied by two singlets in the ^1^H spectrum suggested a C-C bond between parts of the dimer. Further assignments were made based on the HMQC and HMBC spectra, and an 8-3**″** junction was suggested. Structure, numbering and key HMBC and NOESY correlations of **1″** are shown in Figure 3.

**Figure 4:**
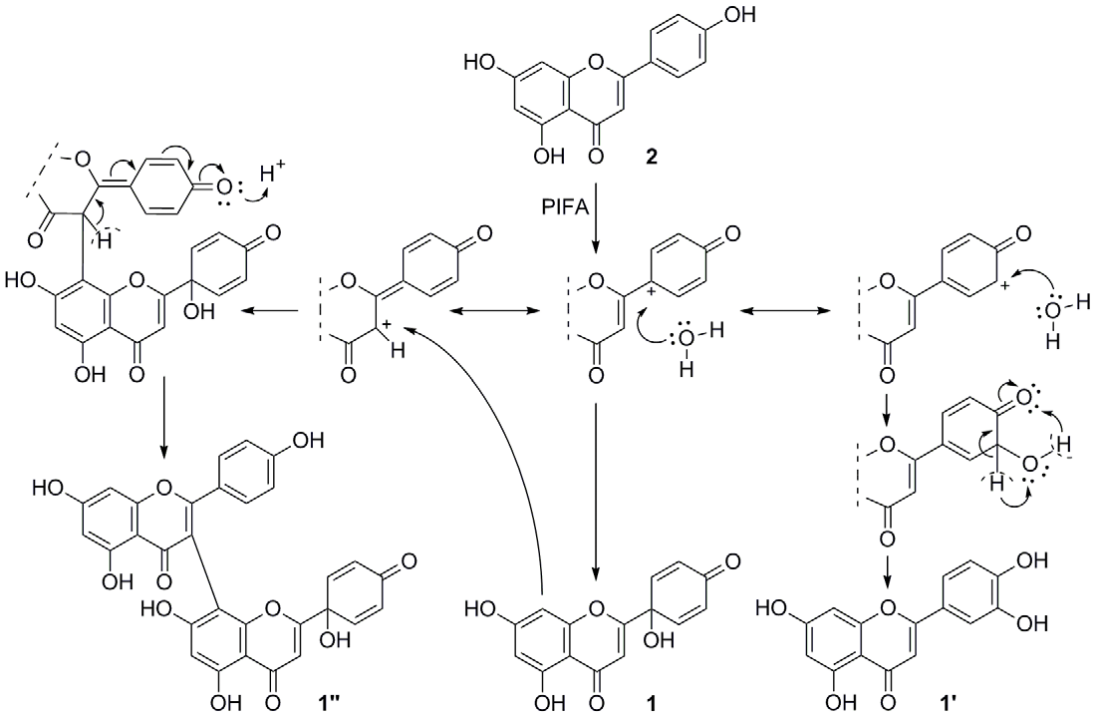
Suggested reaction mechanism for the synthesis of 1 from 2 and product structures obtained.

Hypervalent iodine reagents are widely used for the oxidative dearomatization of 4-substituted phenols in the presence of an appropriate nucleophile to obtain 4,4-disubstituted cyclohexadienones [15]–[16], and reaction mechanisms involving carbocation [13] and/or cation-radical [17] intermediates have been suggested in the literature. Considering the structures of our isolated products we suggest the mechanism shown in Figure 4.

The presence of **1″** as a side-product also provides a possible explanation for the failure of the previous conditions, which most likely failed due to the much higher concentration of starting material and thus the more favorable dimerization or even oligomer formation via C-C coupling side-reactions. Considering the large variety of other side-reactions also available in this system, the 31% yield of **1** shows an unexpectedly good selectivity of PIFA for the 4′-OH group. This selectivity may arise from two phenomena: (1) the greater acidity of the 7-OH group compared to that of the 4′-OH group (in a mixture of MeOH∶water (1∶2, v/v) p*K*_a_ = 6.9 and 8.6, respectively [18]) causes **2** to mostly exist in the 7-mono-anionic form at the neutral pH of the reaction's start, and (2) the intramolecular hydrogen-bond to the 5-OH group makes it more stable than the 4′-OH.

According to our main purpose of establishing a useful method for larger scale production of **1**, we scaled-up the procedure by oxidizing 800.0 mg, 2.0 g and 5.0 g of **2**, which provided 251.3 mg (29.6%), 546.2 mg (25.8%) and 1.18 g (22.3%) of **1**, respectively (see Materials and Methods section). Due to the large volumes of solvent required (in order to prevent excessive side-reactions, as mentioned above), further increases in the amount of starting material used would have been difficult under laboratory conditions. On the other hand, the solvent volume increase was the most probable reason for the slight decrease of the yield on larger scales, because it resulted in slower cooling after the reaction was finished and a much longer evaporation process. This level of productivity should, however, be suitable to obtain appropriate amounts of **1** for the remaining preclinical studies.

The optimized reaction was also performed on 50.0 mg of β-naphthoflavone (**10**), which served as the basis for the most active synthetic derivative to date. This reaction yielded a remarkable 59% of the corresponding analog (**11**), compared to the 16% yield reported previously [12].

**Figure 5:**
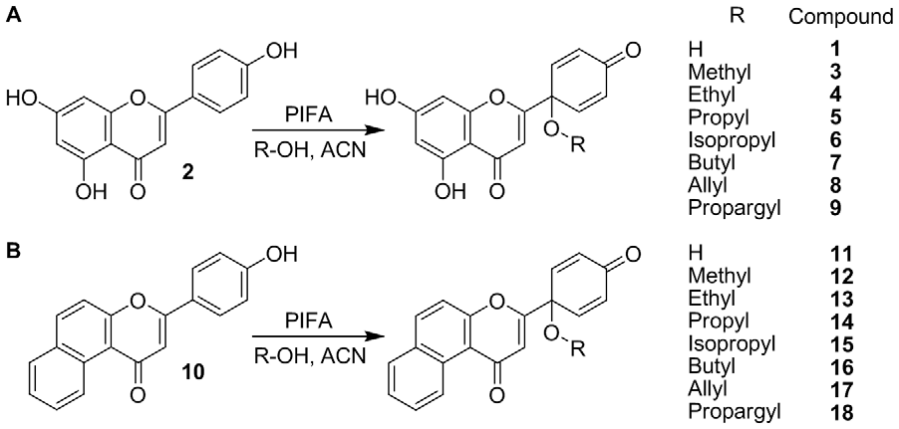
The reaction of 2 and 10 with PIFA in presence of water or various alcohols.

For both starting materials **2** and **10**, further derivatives were also synthesized by replacing water with various alcohols to obtain the corresponding 1′-*O*-alkyl ethers, as shown in Figure 5.

### *In vitro* activity

The cytotoxic activities of all protoapigenone analogs obtained were tested on six human cancer cell lines, including HepG2 and Hep3B (hepatic), Ca9-22 (oral), A549 (lung), as well as MCF-7 and MDA-MB-231 (breast) cancer cells, with doxorubicin as a positive control. IC_50_ values and 95% confidence intervals of the best fit values obtained by nonlinear regression are presented in Table 1.

**Table 1:** *In vitro* cytotoxic activities of the compounds obtained.

Compound	IC_50_ (µM)					
	HepG2	Hep3B	Ca9-22	A549	MCF-7	MDA-MB-231
**1**	3.07 (2.83–3.34)	1.21 (1.19–1.24)	0.78 0.49–1.24)	11.29 (10.07–12.65)	1.70 (1.58–1.82)	1.35 (1.17–1.55)
**1″**	∼19.73	>36	16.89 (14.58–19.57)	>36	>36	∼18.38
**3**	8.99 (6.12–13.21)	3.36 (3.19–3.54)	2.02 1.64–2.49)	21.78 (20.41–23.24)	6.76 (6.07–7.53)	2.82 (2.61–3.04)
**4**	8.27 (8.03–8.51)	1.73 (1.68–1.78)	1.00 (0.81–1.24)	13.45 (12.01–15.07)	3.15 (2.23–4.45)	1.57 (1.45–1.71)
**5**	∼6.90	1.66 (1.65–1.68)	0.81 (0.80–0.82)	12.39 (11.04–13.91)	2.36 (2.06–2.69)	1.26 (1.04–1.53)
**6**	∼19.70	3.59 (3.08–4.19)	1.80 (0.89–3.65)	20.06 (16.22–24.80)	11.43 (10.81–12.09)	2.30 (2.06–2.56)
**7**	8.78 (8.10–9.51)	**0.82** ^(^ * ^)^ (0.80–0.83)	∼0.70	10.76 (9.25–12.51)	**1.38** ^(^ * ^)^ (1.32–1.44)	**0.62** ^(^ *** ^)^ (0.47–0.81)
**8**	7.92 (7.74–8.10)	1.61 (1.54–1.67)	0.93 (0.88–0.98)	17.02 (16.14–17.94)	2.47 (2.26–2.70)	1.53 (1.41–1.66)
**9**	∼7.81	1.20 (1.19–1.21)	0.89 (0.83–0.96)	20.35 (17.33–23.91)	2.32 (2.14–2.54)	1.75 (1.58–1.93)
**11**	1.06 (1.04–1.08)	0.25 (0.20–0.30)	0.23 (0.16–0.33)	2.19 (2.03–2.37)	∼0.61	0.43 (0.41–0.45)
**12**	19.48 (17.02–22.30)	0.98 (0.90–1.06)	0.82 (0.73–0.92)	23.17 (20.91–25.68)	2.03 (1.78–2.32)	1.32 (1.18–1.46)
**13**	9.96 (8.64–11.49)	0.85 (0.83–0.86)	1.03 (0.94–1.21)	17.88 (15.26–20.95)	3.45 (3.10–3.83)	1.68 (1.55–1.81)
**14**	10.53 (8.68–12.77)	0.88 (0.85–0.91)	0.79 (0.77–0.81)	21.72 (16.81–28.07)	2.92 (2.49–3.44)	1.46 (1.34–1.59)
**15**	19.21 (17.00–21.71)	2.48 (1.98–3.11)	2.18 (1.73–2.73)	20.66 (18.00–23.72)	2.33 (2.10–2.59)	2.97 (2.66–3.31)
**16**	7.96 (7.85–8.06)	0.94 0.85–1.03)	1.01 (0.89–1.15)	15.66 (14.90–16.45)	3.05 (2.50–3.72)	1.70 (1.56–1.84)
**17**	9.43 (9.32–9.55)	0.85 (0.81–0.90)	0.83 (0.81–0.86)	15.38 (14.00–16.90)	2.28 (2.14–2.43)	1.46 (1.37–1.56)
**18**	8.14 (7.82–8.47)	0.88 (0.73–1.05)	0.86 (0.85–0.87)	19.30 (17.10–21.79)	2.24 (2.10–2.40)	1.89 (1.68–2.12)
**D**	0.83 0.49–1.39	1.14 (0.78–1.69)	0.60 (0.47–0.76)	∼1.86	1.24 (0.76–2.00)	∼1.73

Individual IC_50_ values were compared to those of **1** (in case of **3**–**9**) or **11** (in case of **12–18**) by one way ANOVA and Dunnet's Multiple Comparison Test and by unpaired T-tests in case of compounds **7** and **1** (Hep3B and MCF7 only). Significantly stronger activities than that of the reference compound are only marked. n = 3;

*: p<0.05 by unpaired T-test, while variances were not significantly different;

*** : p<0.001 by ANOVA;

∼: ambiguous fitting, very wide confidence intervals; **D**: doxorubicin.

**Figure 6:**
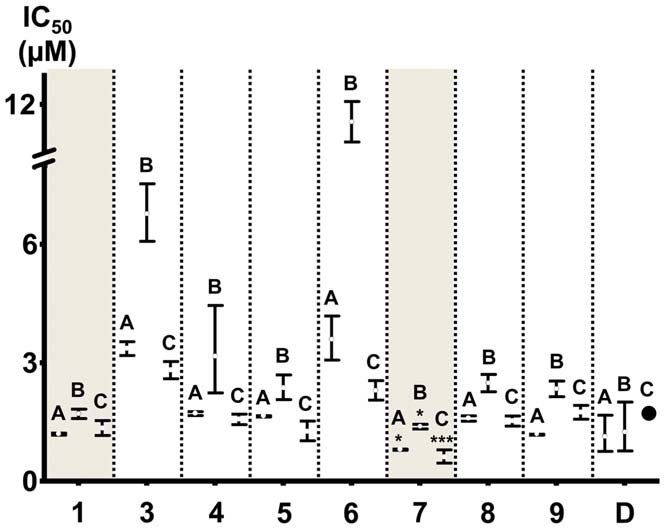
*In vitro* cytotoxic activities of the protoapigenone analogs against the Hep3B, MCF-7 and MDA-MB-231 cell lines. Numbers on the abscissa represent the corresponding compounds, while charts A, B and C represent activities against Hep3B, MCF-7 and MDA-MB-231 cell lines, respectively. Error bars represent the 95% confidence intervals of the IC_50_ values; doxorubicin: ambiguous fitting, no confidence interval available. Significantly stronger activities comparing to that of **1** are marked; *: p<0.05 by unpaired T-test; ***: p<0.001 by ANOVA.

To elaborate on the findings of the previous investigation, which showed that 1′-*O*-methyl substitution decreases the activity of **1** [12], the present results demonstrated that increasing the length of a 1′-*O*-alkyl side-chain on the protoapigenone skeletone typically increases the activity comparing to that of the methyl-substituted derivative (compound **3**). As a result of this tendency, several analogs were found to exert practically the same, or, as in case of the butyl-substituted compound **7** against the Hep3B, MCF-7 and MDA-MB-231 cell lines, even higher *in vitro* cytotoxic activities comparing to that of the non-substituted compound **1**. On the other hand, a 2″ to 3″ unsaturated side-chain (allyl, propargyl derivatives; **8**, **9**) decreased the activity on the A549 cell line comparing to that of compound **5** bearing a saturated propyl side-chain (p<0.05 and 0.001, respectively). In addition to this, presence of a branching side-chain (isopropyl derivative; **6**) strongly decreased the activity on each cell line as compared to that of **5** (p<0.01 in case of Ca9-22 and p<0.001 in case of all other cell lines), resulting in low activities comparable to those of the methyl-substituted compound **3**. Figure 6 shows the activities of all protoapigenone analogs on the Hep3B (A), MCF-7 (B), and MDA-MB-231 (C) cell lines, where **7** was found to exert significantly stronger activity than that of the lead compound **1**.

Interestingly, the activity of the β-naphthoflavone derivatives (**12**–**18**) does not seem to follow the same trend, except for the effect of the side-chain branching (isopropyl; **15**, compared to the propyl; **14**) on most of the cell lines with the exception of A-549, and all of these derivatives showed a marked decrease in the activity as compared to that of **11**.

These results may underline the importance of lipophilicity in the activity of these compounds, which would also explain why derivatives of the more lipophilic β-naphthoflavone as compared to those of apigenin did not show a similar increase in activity when length of the lipophilic side-chain was increased.

The fact, that a 1′-*O* substitution of protoapigenone by a longer, 3 to 4 carbon aliphatic side-chain can restore, or, in case of some cell lines, even increase the *in vitro* activity comparing to that of the non-substituted compound, makes these derivatives, and particularly compound **7**, very interesting new candidates for *in vivo* studies. Not only their increased lipophilicity will result in significantly different pharmacokinetical properties and consequential activity change, but we can also assume, that the 1′-*O*-alkylation may increase the stability of the pharmacophore B-ring by protecting it from decomposition and/or metabolic conversion via re-aromatization to the original flavone, apigenin in this case.

### Conclusions

Our results can be summarized as the following:

1. The first economical method to obtain gram scale amounts of protoapigenone was developed via its direct semisynthesis from apigenin. This achievement is a great step forward for research on this promising anticancer compound, considering the need for large amounts of material to carry out toxicological studies.
2. Several new 1′-*O*-alkyl-protoapigenone as well as the corresponding β-naphthoflavone derivatives were synthesized. The *in vitro* cytotoxicity assay of these compounds revealed, that in case of the protoapigenone derivatives a longer aliphatic side-chain can be beneficial on the activity comparing to that of the non-substituted protoapigenone, while this is not true in case of the naphthoflavone derivatives.
3. Based on these findings, new candidates for further studies were discovered, among which protoapigenone 1′-O-butyl ether (**7**) is the most promising one. The *in vivo* study of this compound is planned in the near future, and the synthesis of further derivatives is currently in progress.

## Materials and Methods

### General

Solvents, reagents and chromatographic stationary phases were purchased from the following companies: solvents and octadecyl silica from Merck Chemicals, PIFA and Sephadex LH-20 gel from Sigma and β-naphthoflavone from Indofine Chemical Company Ltd. (NJ, USA). Apigenin was synthesized from naringenine (Indofine Ltd.) by Chuang DW in previous experiments (not discussed here). All compounds tested possessed a purity of over 95%, except for **1″** (89.1%), **3** (94.8%) and **6** (94.8%). Compound purities were checked using HPLC-UV with one of the solvent systems described below at 245 nm using a gradient system consisting of two Jasco PU2080 LC pump and a Jasco UV2075 ultraviolet detector connected to a Hercule 2000 chromatographic interface. Columns used: Agilent Zorbax XDB-C8, 5 µm, 150×4.6 mm (RP-HPLC), and Agilent Zorbax-sil 5 µm, 250×4.6 mm (NP-HPLC). Solvent systems: RP-HPLC_1_: 55% aqueous MeOH increasing to 80% in 10 minutes; RP-HPLC_2_: 35% v/v aqueous MeOH increasing to 80% in 10 minutes; NP-HPLC: 5% v/v isopropanol in dichloromethane increasing to 17% in 12 minutes and flow rate was 1 mL/min in each case. Chromatograms are shown in Figures S4–S20. ^1^H NMR spectra were taken using a Varian Gemini-2000 200 MHz FT-NMR, and ^13^C and 2D NMR spectra were taken using a Varian Mercury-plus 400 MHz FT-NMR spectrometer. Shifts are given in ppm downfield of TMS and were referenced to internal residual protosolvent. ^1^H NMR spectra of all new compounds are shown in Figures S21–S60. These spectra were taken prior to crystallization, while activity assays and HPLC were done with the pure crystals obtained for all compounds, except for **1″** which could not be crystallized. Hence, Figures S22–S60. do not represent final purity and are only shown to assist interpretation of NMR data. Mass spectra were measured on a PECIEX API 3000 instrument with a turbo ion spray source, Agilent-1100, LC/MSD-Trap, or Shimadzu LCMS-IT-TOF with an ESI interface. Crystallographic data for structural analysis have been deposited with the Cambridge Crystallographic Data Centre (deposition numbers CCDC 762721–762723). These data sets can be obtained free of charge via http://www.ccdc.cam.ac.uk/cgi-bin/catreq.cgi (or from the CCDC, 12 Union Road, Cambridge CB2 1EZ, UK; fax: +44 1223 336 033; e-mail: deposit@ccdc.camac.uk). The melting points were measured on VEB Wagetechnik Rapido PHNK melt microscope (upper limit: 360°C). The structures of **1**, **1′**, **11** and **12** were determined based on literature data [6], [12], [19], their melting points were found 183–184°C (lit. for **1** of natural origin: 180–181°C [6]), 328–330 (lit. for **1′** of natural origin: 328–330°C [20]), 197–198°C and 171–183°C, respectively. HPLC and purity data for the known compounds **1**, **11** and **12** are as follows. **1**. RP-HPLC_1_: 7.057 min (Figure S4), purity: 95.9%. **11**. NP-HPLC: 9.733 min (Figure S13), purity: 98.8%. **12**. NP-HPLC: 10.580 min (Figure S14), purity: 99.5%. Yields given represent isolated yields.

### Assay for Cytotoxicity

Human breast (MCF-7 and MDA-MB-231), liver (HepG2 and Hep3B), and lung (A549) cancer cell lines were obtained from the American Type Culture Collection. The human oral squamous cell carcinoma Ca9-22 was a kind gift from Dr. Jeff Yi-Fu Chen, Kaohsiung Medical University, Kaohsiung, Taiwan. All cell lines were propagated in RPMI-1640 medium supplemented with 10% (v/v) FBS, 100 U/mL penicillin, and 100 µg/mL streptomycin at 37°C in a humidified atmosphere of 5% CO_2_ and 95% air. Cell viability was measured by the MTT colorimetric method [21]. Cells were seeded at densities of 5,000 to 10,000 cells/well in 96-well tissue culture plates. On day two, cells were treated with the test compounds for another 72 h. After drug treatment, attached cells were incubated with MTT (0.5 mg/mL, 1 h) and subsequently solubilized in DMSO. The absorbtion at 550 nm was then measured using a microplate reader. The IC_50_ was recorded as the concentration of the agent that reduced cell viability by 50% under the experimental conditions, and it was determined by applying variable slope nonlinear regression with automatic outlier elimination at Q = 1.0% by using GraphPad Prism 5.0 (GraphPad Software Inc.). Statistical evaluation of the individual IC_50_ values was done by one way ANOVA with Dunnet's Multiple Comparison Test by choosing compounds **1** and **11** as references to their corresponding 1′-O-alkylated analogs. To reveal structure-activity relationships, the activites of each compound were compared by Bonferroni tests, and, in case of comparing the activities of **1** and **7**, unpaired T-tests were also performed.

### General procedure for 1

Compound **2** (100.0 mg) was dissolved in ACN∶water (9∶1, v/v; 100 mL). A quantity of PIFA (318.0 mg, 2 eq.) was added to the container, and microwave heating was applied (70°C, max. power 500 W, 1 min) with continuous stirring. The resulting mixture was evaporated, adsorbed onto 2.0 g of octadecyl silica, layered on the top of 2.0 g of octadecyl silica, washed with 10 mL of 20% aqueous MeOH and eluted with 20 mL of 60% aq. MeOH. The latter fraction was evaporated, redissolved in MeOH, and purified on a Sephadex LH-20 column (385×20 mm; eluent: MeOH). Fractions of 10 mL were collected, and fractions 11 through 14 gave protoapigenone (33.0 mg, 31.2%). The side-products **1′** and **1″** were isolated from fractions 15 through 18 and 19 through 21, respectively, with repeated purification on the same Sephadex LH-20 column.

### Scale-up procedure for 1

Different quantities of **2** (800.0 mg, 2.0 g, and 5.0 g) were dissolved in ACN∶water (9∶1, v/v; 800 mL, 2 L and 5 L) in staunch containers that were then pre-heated to 70°C. After releasing the pressure, PIFA (2 eq.; 2.55 g, 6.37 g and 15.93 g) was added to each solution in a careful manner to prevent sudden explosive boiling, and after re-sealing the containers, microwave heating was applied (70°C, max. 500 W, 3 min) with continuous stirring. The samples obtained were cooled under a stream of water and subsequently with an ice bath. Each sample was immediately evaporated in a 10 L roundbottom flask using a Büchi Rotavapor R-220 at 50°C. After redissolving them, the samples were adsorbed onto octadecyl silica (16.0 g, 40.0 g or 100.0 g) that was layered on top of the same amount of octadecyl silica. SPE was performed by eluting with 15% aqueous MeOH (80 mL, 200 mL or 500 mL) followed by 50% aqueous MeOH (200 mL, 500 mL and 1200 mL. The fractions containing 50% MeOH were purified on silica gel with CH_2_Cl_2_-MeOH (20∶1, v/v) to give 251.3 mg (29.6%), 546.2 mg (25.8%) and 1.18 g (22.3%) of protoapigenone, respectively.

### General procedure for 3 through 9 and 11 through 18

A total of 50.0 mg of **2** or **10** was dissolved in 50 mL of a 9∶1 v/v mixture of acetonitrile and the corresponding alcohol (MeOH, EtOH, PrOH, iPrOH, BuOH, allyl alcohol or propargyl alcohol) or water in the case of **11** and reacted with PIFA (159 mg or 150 mg, 2 eq.) under the same conditions used to obtain **1**. To purify the products, flash chromatography (Biotage Flash+) on silica columns (Biotage Si 24+M 2758-1; 11×150 mm) was performed using a dry-loading technique and *n*-hexane∶EtOAc∶acetone solvent systems (all ratios are given in v/v/v; 7∶3∶1 for **3**–**5**, 9∶3∶1 for **6**, **8** and **9**, and 11∶3∶1 for **7** each containing 0.01% TFA, as well as 16∶3∶1 for **11** and **12**, 27∶3∶1 for **13**, 36∶3∶1 for **14**, **15**, **17** and **18**, and 44∶3∶1 for **16** (no added acid was used)) at flow rates of 24 mL/min. Finally, **3** through **9** and **12** were crystallized from hexane∶acetone (1∶1, v/v), while **13** through **18** were crystallized from hexane∶CH_2_Cl_2_ (1∶1, v/v).

### Experimental

#### Protoapigenone-(8-3″)-apigenin (1″)

Yield: 4% (8.2 mg) as a side-product of the reaction of **2** into **1**; RP-HPLC_2_: 6.843 min (Figure S5), purity: 89.1%; yellow solid, mp: >360°C, ^1^H NMR (acetone-d_6_) *δ* 12.87 (1H, s, OH-5″), 12.65 (1H, s, OH-5), 7.38 (2H, d, *J* = 8.8 Hz, H-2″′ and H-3″′), 6.81 (1H, dd, *J* = 10.0, 3.2 Hz, H-2′ or H-6′), 6.78 (2H, d, *J* = 8.8 Hz, H-3″′ and H-5″′), 6.76 (1H, dd, *J* = 9.6, 3.2 Hz, H-6′ or H-2′), 6.57 (1H, s, H-3), 6.50 (1H, d, *J* = 2.0 Hz, H-8″), 6.30 (1H, s, H-6), 6.29 (1H, d, *J* = 2.0 Hz, H-6″), 6.08 (1H, dd, *J* = 10.0, 2.0 Hz, H-3′ or H-5′), 5.85 (1H, dd, *J* = 10.0, 1.6 Hz, H-5′ or H-3′) (Figures S21 and S22); ^13^C NMR (acetone-d_6_) *δ* 185.2 (C-4′), 184.1 (C-4), 182.5 (C-4″), 168.8 (C-2), 165.8 (C-7″), 165.4 (C-2″), 164.0 (C-8a), 163.9 (C-7, C-5″), 163.5 (C-5), 161.4 (C-4″′), 159.5 (C-8a″), 148.1 (C-2′ or C-6′), 148.0 (C-6′ or C-2′), 131.6 (C-2″′, C-6″′), 130.6 (C-3′ or C-5′), 130.5 (C-5′ or C-3′), 125.3 (C-1″′), 116.7 (C-3″′, C-5″′), 111.3 (C-3″), 108.0 (C-3), 106.1 (C-4a), 105.3 (C-4a″), 101.3 (C-8), 100.5 (C-6), 100.3 (C-6″), 95.4 (C-8″), 71.3 (C-1′); ESI-MS (*m/z*, %): 555 (M^+^+H, 100), 577 (M^+^+Na, 92); HRMS: C_30_H_18_O_11_Na, calculated 577.0747; found 577.0750.

#### Protoapigenone 1′-*O*-methylether (3)

Yield: 42% (23.3 mg); RP-HPLC_1_: 3.860 min (Figure S6), purity: 94.8%; white crystals, mp: 153–155°C, ^1^H NMR (acetone-d_6_) *δ* 12.59 (1H, br, OH-5), 6.99 (2H, d, *J* = 10.2 Hz, H-2′ and H-6′), 6.55 (2H, d, *J* = 10.2 Hz, H-3′ and H-5′), 6.50 (1H, s, H-3), 6.29 (1H, d, *J* = 2.0 Hz, H-8), 6.24 (1H, d, *J* = 2.0 Hz, H-6), 3.42 (3H, s, OCH_3_-1′) (Figures S23 and S24); ^13^C NMR (acetone-d_6_) *δ* 183.4 (C-4′), 181.6 (C-4), 164.7 (C-2), 164.0 (C-7), 162.0 (C-5), 157.5 (C-8a), 144.8 (C-2′ and C-6′), 132.4 (C-3′ and C-5′), 106.9 (C-3), 104.3 (C-4a), 98.8 (C-6), 93.5 (C-8), 74.2 (C-1′), 51.6 (C-1″); ESI-MS (*m/z*, %): 323 (M^+^+Na, 100), 301(M^+^+H, 82); HRMS: C_16_H_12_O_6_Na, calculated 323.0532; found 323.0530.

#### Protoapigenone 1′-*O*-ethylether (4)

Yield: 33% (19.2 mg); RP-HPLC_1_: 5.157 min (Figure S7), purity: 96.7%; white crystals, mp: 170–172°C, ^1^H NMR (acetone-d_6_) *δ* 12.59 (1H, br, OH-5), 7.01 (2H, d, *J* = 10.2 Hz, H-2′ and H-6′), 6.56 (1H, s, H-3), 6.51 (2H, d, *J* = 10.0 Hz, H-3′ and H-5′), 6.28 (1H, br, H-8), 6.24 (1H, br, H-6), 3.63 (2H, q, *J* = 3.5 Hz, H-1″), 1.27 (3H, s, H-2″) (Figures S25, S26 and S27); ^13^C NMR (acetone-d_6_) *δ* 183.6 (C-4′), 181.7 (C-4), 164.9 (C-2), 163.9 (C-7), 162.0 (C-5), 157.5 (C-8a), 145.3 (C-2′ and C-6′), 131.9 (C-3′ and C-5′), 106.9 (C-3), 104.3 (C-4a), 98.7 (C-6), 93.4 (C-8), 74.0 (C-1′), 60.0 (C-1″), 14.6 (C-2″); ESI-MS (*m/z*, %): 337 (M^+^+Na, 100), 315 (M^+^+H, 96); HRMS C_17_H_14_O_6_Na, calculated 337.0688; found 337.0686.

#### Protoapigenone 1′-*O*-propylether (5)

Yield: 36% (21.9 mg); RP-HPLC_1_: 8.240 min (Figure S8), purity: 97.8%; white crystals, mp: 181–182°C, ^1^H NMR (acetone-d_6_) *δ* 12.59 (1H, br, OH-5), 7.01 (2H, d, *J* = 10.2 Hz, H-2′ and H-6′), 6.56 (1H, s, H-3), 6.52 (2H, d, *J* = 10.2 Hz, H-3′ and H-5′), 6.28 (1H, d, *J* = 2 Hz, H-8), 6.24 (1H, d, *J* = 2 Hz, H-6), 3.54 (2H, t, *J* = 6.3 Hz, H-1″), 1.68 (2H, m, H-2″), 0.98 (3H, t, *J* = 7.3 Hz, H-3″) (Figures S28, S29 and S30); ^13^C NMR (acetone-d_6_) *δ* 183.6 (C-4′), 181.7 (C-4), 164.8 (C-2), 163.9 (C-7), 162.0 (C-5), 157.5 (C-8a), 145.4 (C-2′ and C-6′), 131.9 (C-3′ and C-5′), 106.9 (C-3), 104.3 (C-4a), 98.7 (C-6), 93.5 (C-8), 74.0 (C-1′), 65.9 (C-1″), 22.7 (C-2″), 9.6 (C-3″); ESI-MS (*m/z*, %): 329 (M^+^+H, 100), 351 (M^+^+Na, 80); HRMS C_18_H_16_O_6_Na, calculated 351.0845; found 351.0846.

#### Protoapigenone 1′-*O*-isopropylether (6)

Yield: 26% (15.8 mg); RP-HPLC_1_: 7.637 min (Figure S9), purity: 94.9%; light-brownish crystals, mp: 138–140°C, ^1^H NMR (acetone-d_6_) *δ* 12.59 (1H, br, OH-5), 7.07 (2H, d, *J* = 9.8 Hz, H-2′ and H-6′), 6.56 (1H, s, H-3), 6.50 (2H, d, *J* = 9.8 Hz, H-3′ and H-5′), 6.28 (1H, br, H-8), 6.24 (1H, br, H-6), 3.94 (1H, m, *J* = 6.1 Hz, H-1″), 1.24 (6H, d, *J* = 6.2 Hz, H-2″ and H-3″) (Figures S31, S32, S33 and S34); ^13^C NMR (acetone-d_6_) *δ* 162.0 (C-5), 145.7 (C-2′ and C-6′), 131.3 (C-3′ and C-5′), 107.0 (C-3), 104.2 (C-4a), 98.7 (C-6), 93.4 (C-8), 68.0 (C-1″), 23.5 (C-2″ and C-3″); ESI-MS (*m/z*, %): 329 (M^+^+H, 100), 351 (M^+^+Na, 84), 270 (M^+^+H – OC_3_H_6_, 16); HRMS C_18_H_16_O_6_Na, calculated 351.0845; found 351.0844.

#### Protoapigenone 1′-*O*-butylether (7)

Yield: 44% (27.9 mg); RP-HPLC_1_: 9.437 min (Figure S10), purity: 96.1%; white crystals, mp: 167–168°C, ^1^H NMR (acetone-d_6_) *δ* 12.60 (1H, br, OH-5), 7.01 (2H, d, *J* = 10.8 Hz, H-2′ and H-6′), 6.55 (1H, s, H-3), 6.52 (2H, d, *J* = 10.8 Hz, H-3′ and H-5′), 6.28 (1H, br, H-8), 6.25 (1H, br, H-6), 3.58 (2H, t, *J* = 6.0 Hz, H-1″), 1.63 (2H, m, br, H-2″), 1.44 (2H, m, br, H-3″), 0.93 (3H, t, *J* = 7.2 Hz, H-4″) (Figures S35, S36 and S37); ^13^C NMR (acetone-d_6_) *δ* 183.6 (C-4′), 181.7 (C-4), 164.9 (C-2), 163.9 (C-7), 162.0 (C-5), 157.5 (C-8a), 145.4 (C-2′ and C-6′), 132.0 (C-3′ and C-5′), 106.9 (C-3), 104.3 (C-4a), 98.7 (C-6), 93.4 (C-8), 74.0 (C-1′), 64.1 (C-1″), 31.5 (C-2″), 18.6 (C-3″), 12.8 (C-4″); HRMS C_19_H_18_O_6_Na, calculated 365.1001; found 365.1000.

#### Protoapigenone 1′-*O*-allylether (8)

Yield: 29% (17.5 mg); RP-HPLC_1_: 8.283 min (Figure S11), purity: 97.2%; transparent crystals, ^1^H NMR (acetone-d_6_) *δ* 12.59 (1H, br, OH-5), 7.04 (2H, d, *J* = 10.2 Hz, H-2′ and H-6′), 6.57 (1H, s, H-3), 6.53 (2H, d, *J* = 10.4 Hz, H-3′ and H-5′), 6.29 (1H, d, *J* = 2.2 Hz, H-8), 6.24 (1H, *J* = 2.2 Hz, H-6), 6.00 (1H, m, *J* = 5.4 Hz, H-2″), 5.37 (1H, d, *J* = 17.2 Hz, H-3″, cis), 5.20 (1H, d, *J* = 10.4 Hz, H-3″, trans), 4.14 (2H, *J* = 5.2 Hz, H-1″) (Figures S38 and S39); ^13^C NMR (acetone-d_6_) *δ* 164.9 (C-2), 163.9 (C-7), 162.0 (C-5), 157.5 (C-8a), 145.4 (C-2′ and C-6′), 132.0 (C-3′ and C-5′), 106.9 (C-3), 98.7 (C-6), 93.4 (C-8), 72.9 (C-1′), 65.9 (C-1″); ESI-MS (*m/z*, %): 349 (M^+^+Na, 100), 327 (M^+^+H, 85); HRMS C_18_H_14_O_6_Na, calculated 349.0688; found 349.0690; X-ray data are shown in Figure S1.

#### Protoapigenone 1′-*O*-propargylether (9)

Yield: 36% (21.6 mg); RP-HPLC_1_: 4.180 min (Figure S12), purity: 97.4%; yellow crystalline powder, mp: 205–207°C, ^1^H NMR (acetone-d_6_) *δ* 12.55 (1H, s, OH-5), 7.08 (2H, d, *J* = 10.0 Hz, H-2′ and H-6′), 6.55 (2H, d, *J* = 10.2 Hz, H-3′ and H-5′), 6.53 (1H, s, H-3), 6.30 (1H, d, *J* = 2.2 Hz, H-8), 6.24 (1H, d, *J* = 2.0 Hz, H-6), 4.34 (2H, d, *J* = 2.4 Hz, H-1″), 3.11 (1H, t, *J* = 2.4 Hz, H-3″) (Figures S40 and S41, 400 MHz ^1^H spectrum is also provided in Figure S42); ^13^C NMR (acetone-d_6_) *δ* 185.4 (C-4′), 183.5 (C-4), 166.1 (C-2), 166.0 (C-7), 163.9 (C-5), 159.4 (C-8a), 145.9 (C-2′ and C-6′), 134.3 (C-3′ and C-5′), 109.0 (C-3), 106.2 (C-4a), 100.8 (C-6), 95.4 (C-8), 81.3 (C-1′), 78.0 (C-3″), 76.4 (C-2″), 54.8 (C-1″); HRMS C_18_H_12_O_6_Na, calculated 347.0532; found 347.0535.

#### 3-(1-Ethoxy-4-oxocyclohexa-2,5-dienyl)-*1H*-benzo[*f*]chromen-1-one (13)

Yield: 65% (37.5 mg); NP-HPLC: 7.507 min (Figure S15), purity: 99.8%; white crystals, mp: 158–160°C, ^1^H NMR (CDCl3) *δ* 9.97 (1H, d, *J* = 8.4 Hz, H-5), 8.04 (1H, d, *J* = 8.8 Hz, H-9), 7.87 (1H, dd, *J* = 7.6, 1.0 Hz, H-8), 7.74 (1H, td, *J* = 8.4, 1.4 Hz, H-7), 7.60 (1H, td, *J* = 8.0, 1.0 Hz, H-6), 7.36 (1H, d, *J* = 9.2 Hz, H-10), 6.95 (1H, s, H-3), 6.89 (2H, d, *J* = 10.2 Hz, H-2′ and H-6′), 6.55 (2H, d, *J* = 10.4 Hz, H-3′ and H-5′), 3.61 (2H, q, *J* = 7.0 Hz, H-1″), 1.307 (3H, t, *J* = 7.0 Hz, H-2″) (Figures S43, S44 and S45); ^13^C NMR (CDCl3) *δ* 184.2 (C-4′), 179.5 (C-4), 160.9 (C-2), 156.9 (C-10a), 145.8 (C-2′ and C-6′), 132.2 (C-3′ and C-5′), 135.3, 130.2, 129.9, 129.0, 127.8, 126.6, 126.4 (C-4b, C-5, C-6, C-7, C-8, C-8a, C-9), 116.9 (C-10), 116.9 (C-4a), 112.1 (C-3), 73.9 (C-1′), 60.5 (C-1″), 15,3 (C-2″); ESI-MS (*m/z*, %): 355 (M^+^+Na, 100), 333 (M^+^+H, 79); HRMS C_21_H_16_O_4_Na, calculated 355.0946; found 355.0945.

#### 3-(1-Propoxy-4-oxocyclohexa-2,5-dienyl)-*1H*-benzo[*f*]chromen-1-one (14)

Yield: 64% (38.4 mg); NP-HPLC: 5.977 min (Figure S16), purity: 98.9%; transparent crystals, ^1^H NMR (CDCl3) *δ* 9.96 (1H, d, *J* = 8.8 Hz, H-5), 8.01 (1H, d, *J* = 9.0 Hz, H-9), 7.85 (1H, d, br, *J* = 8.0 Hz, H-8), 7.73 (1H, td, *J* = 7.0, 1.6 Hz, H-7), 7.58 (1H, td, *J* = 8.0, 1.2 Hz, H-6), 7.33 (1H, d, *J* = 9.2 Hz, H-10), 6.92 (1H, s, H-3), 6.87 (2H, d, *J* = 9.8 Hz, H-2′ and H-6′), 6.55 (2H, d, *J* = 10.2 Hz, H-3′ and H-5′), 3.50 (2H, t, *J* = 6.2 Hz, H-1″), 1.68 (2H, sextet, *J* = 7.0 Hz, H-2″), 0.99 (3H, t, *J* = 7.3 Hz, H-3″) (Figures S46, S47 and S48); ^13^C NMR (CDCl3) *δ* 145.8 (C-2′ and C-6′), 132.2 (C-3′ and C-5′), 135.3, 129.0, 127.8, 126.6, 126.4 (C-4b, C-7, C-8, C-8a, C-9), 116.9 (C-10), 116.9 (C-4a), 112.1 (C-3), 73.9 (C-1′), 66.5 (C-1″), 23.0 (C-2″), 10.2 (C-3″); ESI-MS (*m/z*, %): 369 (M^+^+Na, 100), 347 (M^+^+H, 60); HRMS C_22_H_18_O_4_Na, calculated 369.1103; found 369.1105; X-ray data are shown in Figure S2.

#### 3-(1-Isopropoxy-4-oxocyclohexa-2,5-dienyl)-*1H*-benzo[*f*]chromen-1-one (15)

Yield: 52% (31.2 mg); NP-HPLC: 6.307 min (Figure S17), purity: 99.0%; transparent crystals, ^1^H NMR (CDCl_3_) *δ* 9.97 (1H, d, *J* = 8.8 Hz, H-5), 8.03 (1H, d, *J* = 9.0 Hz, H-9), 7.94 (1H, d, br, *J* = 8.2 Hz, H-8), 7.74 (1H, td, *J* = 7.8, 1.0 Hz, H-7), 7.60 (1H, td, *J* = 7.6, 1.0 Hz, H-6), 7.35 (1H, d, *J* = 9.2 Hz, H-10), 6.94 (1H, s, H-3), 6.91 (2H, d, *J* = 10.6 Hz, H-2′ and H-6′), 6.53 (2H, d, *J* = 9.8 Hz, H-3′ and H-5′), 3.89 (1H, septet, *J* = 6.2 Hz, H-1″), 1.24 (6H, d, *J* = 6.2 Hz, H-2″ and H-3″) (Figures S49, S50 and S51); ^13^C NMR (CDCl3) *δ* 184.5 (C-4′), 179.6 (C-4), 161.2 (C-2), 156.9 (C-10a), 146.2 (C-2′ and C-6′), 131.5 (C-3′,C-5′), 135.3, 130.2, 129.9, 129.0, 127.7, 126.6, 126.4 (C-4b, C-5, C-6, C-7, C-8, C-8a, C-9), 116.9 (C-10), 116.9 (C-4a), 112.2 (C-3), 74.1 (C-1′), 68.5 (C-1″), 24.3 (C-2″ and C-3″); ESI-MS (*m/z*, %): 369 (M^+^+Na, 100), 347 (M^+^+H, 54); HRMS C_22_H_18_O_4_Na, calculated 369.1103; found 369.1104.

#### 3-(1-Butoxy-4-oxocyclohexa-2,5-dienyl)-*1H*-benzo[*f*]chromen-1-one (16)

Yield: 54% (33.8 mg); NP-HPLC: 5.230 min (Figure S18), purity: 95.8%; transparent crystals, mp: 72–73°C, ^1^H NMR (CDCl_3_) *δ* 9.97 (1H, d, *J* = 8.8 Hz, H-5), 8.03 (1H, d, *J* = 9.2 Hz, H-9), 7.86 (1H, d, br, *J* = 8.0 Hz, H-8), 7.74 (1H, td, *J* = 8.4, 1.6 Hz, H-7), 7.59 (1H, td, *J* = 7.6, 1.6 Hz, H-6), 7.34 (1H, d, *J* = 9.0 Hz, H-10), 6.92 (1H, s, H-3), 6.87 (2H, d, *J* = 10.2 Hz, H-2′ and H-6′), 6.55 (2H, d, *J* = 10.4 Hz, H-3′ and H-5′), 3.54 (2H, t, *J* = 6.2 Hz, H-1″), 1.65 (2H, m, *J* = 7.0 Hz, H-2″), 1.45 (2H, m, *J* = 7.3 Hz, H-3″), 0.95 (3H, t, *J* = 7.4 Hz, H-4″) (Figures S52, S53 and S54); ^13^C NMR (CDCl3) *δ* 184.3 (C-4′), 179.5 (C-4), 160.1 (C-2), 156.9 (C-10a), 145.9 (C-2′ and C-6′), 132.2 (C-3′ and C-5′), 135.3, 130.2, 129.9, 129.0, 127.8, 126.6, 126.4 (C-4b, C-5, C-6, C-7, C-8, C-8a, C-9), 116.9 (C-10), 116.9 (C-4a), 112.1 (C-3), 73.8 (C-1′), 64.6 (C-1″), 31.7 (C-2″), 18.8 (C-3″), 13.4 (C-4″); ESI-MS (*m/z*, %): 383 (M^+^+Na, 100), 361 (M^+^+H, 65); HRMS C_23_H_20_O_4_Na, calculated 383.1259; found 383.1258.

#### 3-(1-Allyloxy-4-oxocyclohexa-2,5-dienyl)-*1H*-benzo[*f*]chromen-1-one (17)

Yield: 51% (30.5 mg); NP-HPLC: 6.650 min (Figure S19), purity: 98.0%; transparent crystals, mp: 148–150°C, ^1^H NMR (CDCl_3_) *δ* 9.96 (1H, d, *J* = 8.2 Hz, H-5), 8.03 (1H, d, *J* = 8.8 Hz, H-9), 7.86 (1H, d, br, *J* = 8.2 Hz, H-8), 7.74 (1H, td, *J* = 8.4, 1.4 Hz, H-7), 7.60 (1H, td, *J* = 7.6, 1.0 Hz, H-6), 7.35 (1H, d, *J* = 9.2 Hz, H-10), 6.94 (1H, s, H-3), 6.91 (2H, d, *J* = 11.0 Hz, H-2′ and H-6′), 6.57 (2H, d, *J* = 10.2 Hz, H-3′ and H-5′), 5.95 (1H, m, *J* = 5.0 Hz, H-2″), 5.38 (1H, dd, *J* = 18.8, 1.6 Hz, H-3″, cis), 5.25 (1H, dd, *J* = 10.4, 1.6 Hz, H-3″, trans), 4.09 (2H, *J* = 5.6 Hz, H-1″) (Figures S55, S56 and S57); ^13^C NMR (CDCl3) *δ* 184.1 (C-4′), 179.4 (C-4), 160.6 (C-2), 156.9 (C-10a), 145.3 (C-2′ and C-6′), 132.4 (C-3′ and C-5′), 135.3, 130.2, 129.9, 129.0, 127.8, 126.6, 126.4 (C-4b, C-5, C-6, C-7, C-8, C-8a, C-9), 116.9 (C-10), 116.9 (C-4a), 112.2 (C-3), 74.1 (C-1′), 65.7(C-1″), 133.2 (C-2″), 117.2 (C-3″); ESI-MS (*m/z*, %): 367 (M^+^+Na, 100), 345 (M^+^+H, 65); HRMS C_22_H_16_O_4_Na, calculated 367.0946; found 367.0949; X-ray data are shown in Figure S3.

#### 3-(1-(Prop-2-ynoxy)-4-oxocyclohexa-2,5-dienyl)-*1H*-benzo[*f*]chromen-1-one (18)

Yield: 45% (26.7 mg), NP-HPLC: 9.527 min (Figure S20), purity: 99.6%; transparent crystals, mp: 142–145°C, ^1^H NMR (CDCl_3_) *δ* 9.95 (1H, d, *J* = 7.4 Hz, H-5), 8.04 (1H, d, *J* = 9.2 Hz, H-9), 7.87 (1H, d, br, *J* = 8.0 Hz, H-8), 7.74 (1H, td, *J* = 8.4, 1.6 Hz, H-7), 7.60 (1H, td, *J* = 8.2, 1.0 Hz, H-6), 7.37 (1H, d, *J* = 9.2 Hz, H-10), 6.97 (2H, d, *J* = 10.4 Hz, H-2′ and H-6′), 6.91 (1H, s, H-3), 6.59 (2H, d, *J* = 10.4 Hz, H-3′ and H-5′), 4.26 (2H, d, *J* = 2.6 Hz, H-1″), 2.55 (1H, t, *J* = 2.4 Hz, H-3″) (Figures S58, S59 and S60); ^13^C NMR (CDCl3) *δ* 183.9 (C-4′), 179.3 (C-4), 159.9 (C-2), 156.9 (C-10a), 144.2 (C-2′ and C-6′), 132.6 (C-3′ and C-5′), 135.4, 130.2, 129.8, 129.1, 127.8, 126.6, 126.5 (C-4b, C-5, C-6, C-7, C-8, C-8a, C-9), 116.8 (C-10), 116.8 (C-4a), 112.2 (C-3), 74.6 (C-1′), 53.2 (C-1″), 75.7 (C-3″),79.0 (C-2″); ESI-MS (*m/z*, %): 365 (M^+^+Na, 100), 343 (M^+^+H, 52); HRMS C_22_H_14_O_4_Na, calculated 365.0790; found 365.0788.

## Supporting Information

Figure S1 **X-ray diffraction data for compound 8.** CCDC 762721; formula: C_18_H_14_O_6_; unit cell parameters: a 6.9461(20) b 30.2804(66) c 7.6340(13) beta 93.752(19) space group P21/c.
(TIF)

Figure S2 **X-ray diffraction data for compound 14.** CCDC 762722; formula: C_22_H_18_O_4_; unit cell parameters: a 7.8452(8) b 25.285(5) c 18.144(2) beta 96.096(9) space group P21/c.
(TIF)

Figure S3 **X-ray diffraction data for compound 17.** CCDC 762723; formula: C_22_H_16_O_4_; unit cell parameters: a 7.9122(8) b 24.817(5) c 17.8259(16) beta 96.380(8) space group P21/c.
(TIF)

Figure S4 **RP-HPLC chromatogram of compound 1.** Solvent: 35% v/v aqueous MeOH increasing to 80% in 10 min and subsequently changing back to 35%; flow rate: 1 mL/min, λ = 245 nm
(TIF)

Figure S5 **RP-HPLC chromatogram of compound 1″.** Solvent: 35% v/v aqueous MeOH increasing to 80% in 10 min and subsequently changing back to 35%; flow rate:1 mL/min, λ = 245 nm
(TIF)

Figure S6 **RP-HPLC chromatogram of compound 3.** Solvent: 55% v/v aqueous MeOH increasing to 80% in 10 min and subsequently changing back to 55%; flow rate:1 mL/min, λ = 245 nm
(TIF)

Figure S7 **RP-HPLC chromatogram of compound 4.** Solvent: 55% v/v aqueous MeOH increasing to 80% in 10 min and subsequently changing back to 55%; flow rate:1 mL/min, λ = 245 nm
(TIF)

Figure S8 **RP-HPLC chromatogram of compound 5.** Solvent: 55% v/v aqueous MeOH increasing to 80% in 10 min and subsequently changing back to 55%; flow rate:1 mL/min, λ = 245 nm
(TIF)

Figure S9 **RP-HPLC chromatogram of compound 6.** Solvent: 55% v/v aqueous MeOH increasing to 80% in 10 min and subsequently changing back to 55%; flow rate:1 mL/min, λ = 245 nm
(TIF)

Figure S10 **RP-HPLC chromatogram of compound 7.** Solvent: 55% v/v aqueous MeOH increasing to 80% in 10 min and subsequently changing back to 55%; flow rate:1 mL/min, λ = 245 nm
(TIF)

Figure S11 **RP-HPLC chromatogram of compound 8.** Solvent: 55% v/v aqueous MeOH increasing to 80% in 10 min and subsequently changing back to 55%; flow rate:1 mL/min, λ = 245 nm
(TIF)

Figure S12 **RP-HPLC chromatogram of compound 9.** Solvent: 55% v/v aqueous MeOH increasing to 80% in 10 min and subsequently changing back to 55%; flow rate:1 mL/min, λ = 245 nm
(TIF)

Figure S13 **NP-HPLC chromatogram of compound 11.** Solvent: 5% v/v isopropanol in dichloromethane increasing to 17% in 12 min; flow rate:1 mL/min, λ = 245 nm
(TIF)

Figure S14 **NP-HPLC chromatogram of compound 12.** Solvent: 5% v/v isopropanol in dichloromethane increasing to 17% in 12 min; flow rate:1 mL/min, λ = 245 nm
(TIF)

Figure S15 **NP-HPLC chromatogram of compound 13.** Solvent: 5% v/v isopropanol in dichloromethane increasing to 17% in 12 min; flow rate:1 mL/min, λ = 245 nm
(TIF)

Figure S16 **NP-HPLC chromatogram of compound 14.** Solvent: 5% v/v isopropanol in dichloromethane increasing to 17% in 12 min; flow rate:1 mL/min, λ = 245 nm
(TIF)

Figure S17 **NP-HPLC chromatogram of compound 15.** Solvent: 5% v/v isopropanol in dichloromethane increasing to 17% in 12 min; flow rate:1 mL/min, λ = 245 nm
(TIF)

Figure S18 **NP-HPLC chromatogram of compound 16.** Solvent: 5% v/v isopropanol in dichloromethane increasing to 17% in 12 min; flow rate:1 mL/min, λ = 245 nm
(TIF)

Figure S19 **NP-HPLC chromatogram of compound 17.** Solvent: 5% v/v isopropanol in dichloromethane increasing to 17% in 12 min; flow rate:1 mL/min, λ = 245 nm
(TIF)

Figure S20 **NP-HPLC chromatogram of compound 18.** Solvent: 5% v/v isopropanol in dichloromethane increasing to 17% in 12 min; flow rate:1 mL/min, λ = 245 nm
(TIF)

Figure S21 **400 MHz ^1^H NMR spectrum of compound 1″.**
(TIF)

Figure S22 **Zoom of 400 MHz ^1^H NMR spectrum of compound 1″.**
(TIF)

Figure S23 **200 MHz ^1^H NMR spectrum of compound 3 before crystallization.**
(TIF)

Figure S24 **Zoom of 200 MHz ^1^H NMR spectrum of compound 3 before crystallization.**
(TIF)

Figure S25 **200 MHz ^1^H NMR spectrum of compound 4 before crystallization.**
(TIF)

Figure S26 **Zoom of 200 MHz ^1^H NMR spectrum of compound 4 before crystallization.**
(TIF)

Figure S27 **Zoom of 200 MHz ^1^H NMR spectrum of compound 4 before crystallization.**
(TIF)

Figure S28 **200 MHz ^1^H NMR spectrum of compound 5 before crystallization.**
(TIF)

Figure S29 **Zoom of 200 MHz ^1^H NMR spectrum of compound 5 before crystallization.**
(TIF)

Figure S30 **Zoom of 200 MHz ^1^H NMR spectrum of compound 5 before crystallization.**
(TIF)

Figure S31 **200 MHz ^1^H NMR spectrum of compound 6 before crystallization.**
(TIF)

Figure S32 **Zoom of 200 MHz ^1^H NMR spectrum of compound 6 before crystallization.**
(TIF)

Figure S33 **Zoom of 200 MHz ^1^H NMR spectrum of compound 6 before crystallization.**
(TIF)

Figure S34 **Zoom of 200 MHz ^1^H NMR spectrum of compound 6 before crystallization.**
(TIF)

Figure S35 **200 MHz ^1^H NMR spectrum of compound 7 before crystallization.**
(TIF)

Figure S36 **Zoom of 200 MHz ^1^H NMR spectrum of compound 7 before crystallization.**
(TIF)

Figure S37 **Zoom of 200 MHz ^1^H NMR spectrum of compound 7 before crystallization.**
(TIF)

Figure S38 **200 MHz ^1^H NMR spectrum of compound 8 before crystallization.**
(TIF)

Figure S39 **Zoom of 200 MHz ^1^H NMR spectrum of compound 8 before crystallization.**
(TIF)

Figure S40 **200 MHz ^1^H NMR spectrum of compound 9 before crystallization.**
(TIF)

Figure S41 **Zoom of 200 MHz ^1^H NMR spectrum of compound 9 before crystallization.**
(TIF)

Figure S42 **400 MHz ^1^H NMR spectrum of compound 9 before crystallization.**
(TIF)

Figure S43 **200 MHz ^1^H NMR spectrum of compound 13 before crystallization.**
(TIF)

Figure S44 **Zoom of 200 MHz ^1^H NMR spectrum of compound 13 before crystallization.**
(TIF)

Figure S45 **Zoom of 200 MHz ^1^H NMR spectrum of compound 13 before crystallization.**
(TIF)

Figure S46 **200 MHz ^1^H NMR spectrum of compound 14 before crystallization.**
(TIF)

Figure S47 **Zoom of 200 MHz ^1^H NMR spectrum of compound 14 before crystallization.**
(TIF)

Figure S48 **Zoom of 200 MHz ^1^H NMR spectrum of compound 14 before crystallization.**
(TIF)

Figure S49 **200 MHz ^1^H NMR spectrum of compound 15 before crystallization.**
(TIF)

Figure S50 **Zoom of 200 MHz ^1^H NMR spectrum of compound 15 before crystallization.**
(TIF)

Figure S51 **Zoom of 200 MHz ^1^H NMR spectrum of compound 15 before crystallization.**
(TIF)

Figure S52 **200 MHz ^1^H NMR spectrum of compound 16 before crystallization.**
(TIF)

Figure S53 **Zoom of 200 MHz ^1^H NMR spectrum of compound 16 before crystallization.**
(TIF)

Figure S54 **Zoom of 200 MHz ^1^H NMR spectrum of compound 16 before crystallization.**
(TIF)

Figure S55 **200 MHz ^1^H NMR spectrum of compound 17 before crystallization.**
(TIF)

Figure S56 **Zoom of 200 MHz ^1^H NMR spectrum of compound 17 before crystallization.**
(TIF)

Figure S57 **Zoom 200 MHz ^1^H NMR spectrum of compound 17 before crystallization.**
(TIF)

Figure S58 **200 MHz ^1^H NMR spectrum of compound 18 before crystallization.**
(TIF)

Figure S59 **Zoom of 200 MHz ^1^H NMR spectrum of compound 18 before crystallization.**
(TIF)

Figure S60 **Zoom of 200 MHz ^1^H NMR spectrum of compound 18 before crystallization.**
(TIF)
