# Supplementary figures and images for: Direct Semi-Synthesis of the Anticancer Lead-Drug Protoapigenone from Apigenin, and Synthesis of Further New Cytotoxic Protoflavone Derivatives
Source: PLoS One. 2011 Aug 30;6(8):e23922. doi: 10.1371/journal.pone.0023922 (PMC3166065; doi:10.1371/journal.pone.0023922)

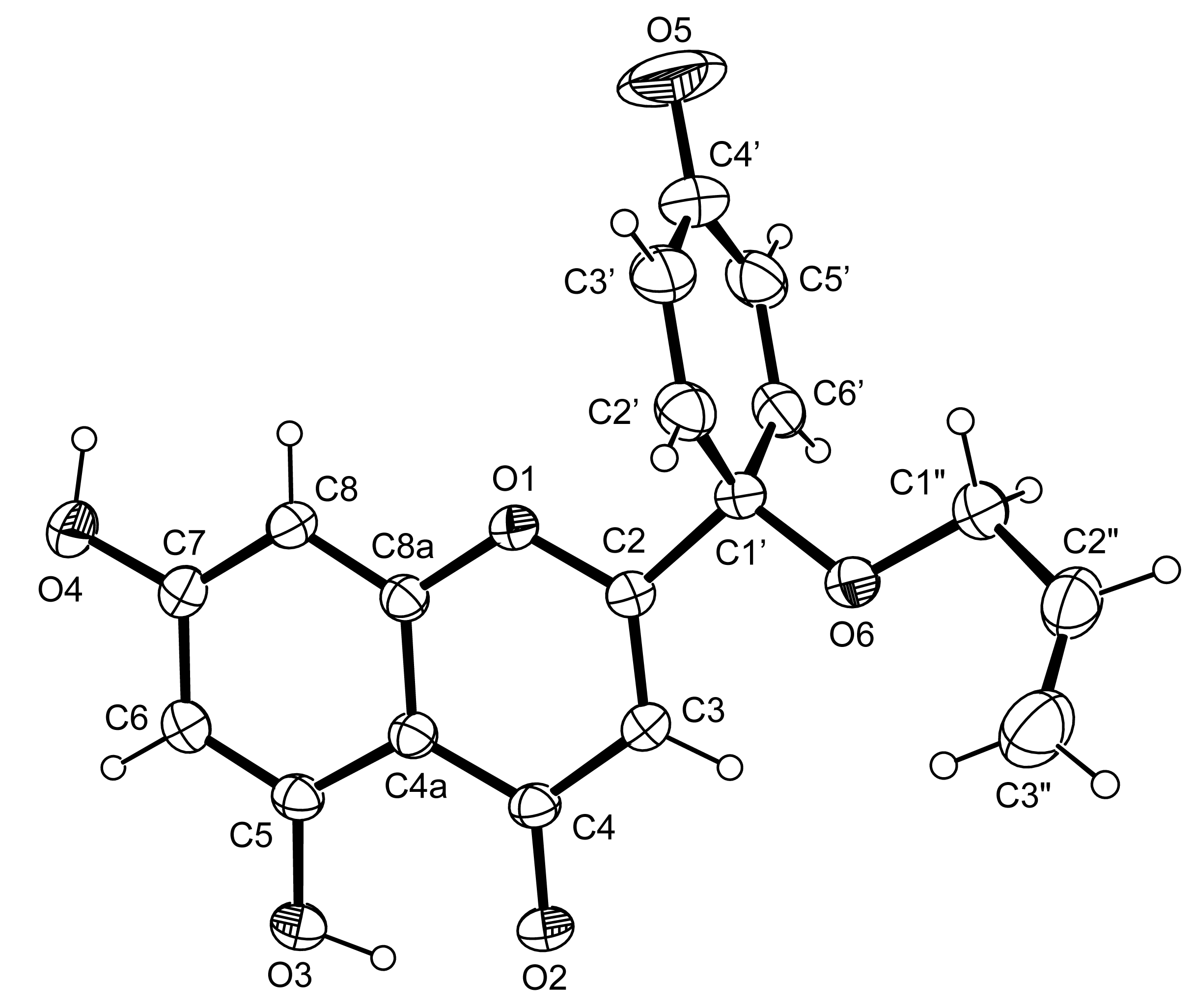

Supplement: Figure S1 — X-ray diffraction data for compound 8. CCDC 762721; formula: C18H14O6; unit cell parameters: a 6.9461(20) b 30.2804(66) c 7.6340(13) beta 93.752(19) space group P21/c. (TIF) [file pone.0023922.s001.tif]

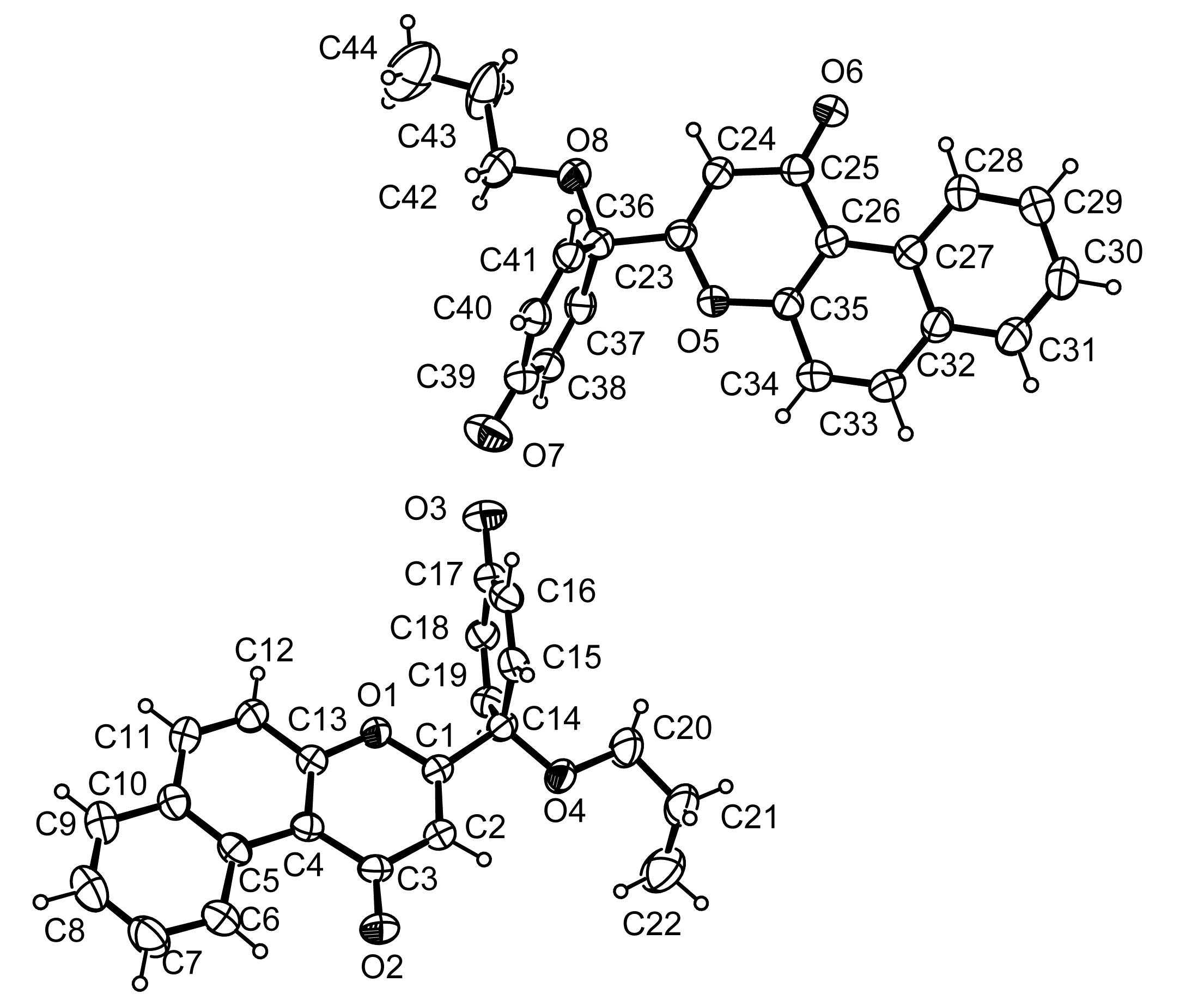

Supplement: Figure S2 — X-ray diffraction data for compound 14. CCDC 762722; formula: C22H18O4; unit cell parameters: a 7.8452(8) b 25.285(5) c 18.144(2) beta 96.096(9) space group P21/c. (TIF) [file pone.0023922.s002.tif]

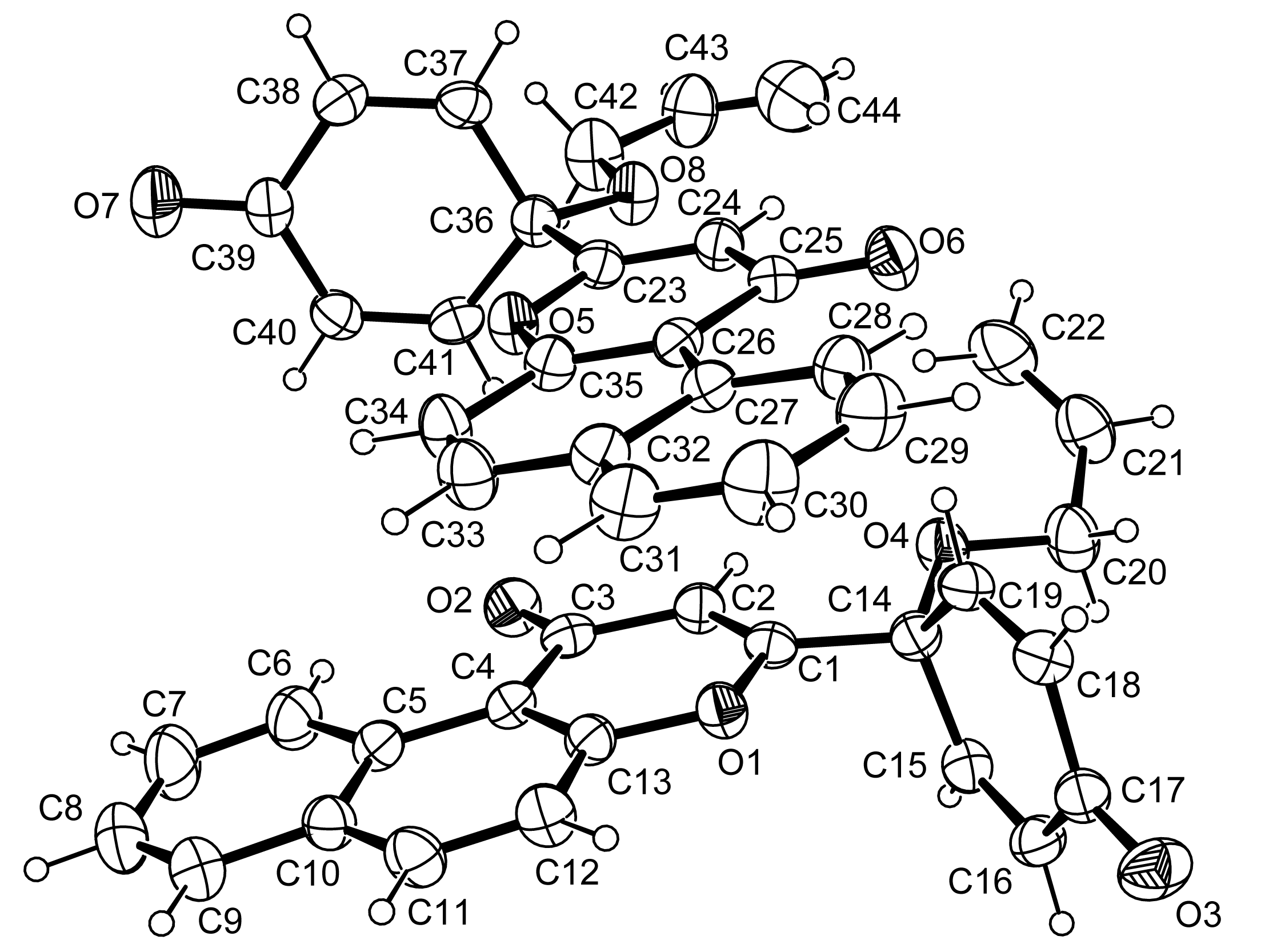

Supplement: Figure S3 — X-ray diffraction data for compound 17. CCDC 762723; formula: C22H16O4; unit cell parameters: a 7.9122(8) b 24.817(5) c 17.8259(16) beta 96.380(8) space group P21/c. (TIF) [file pone.0023922.s003.tif]

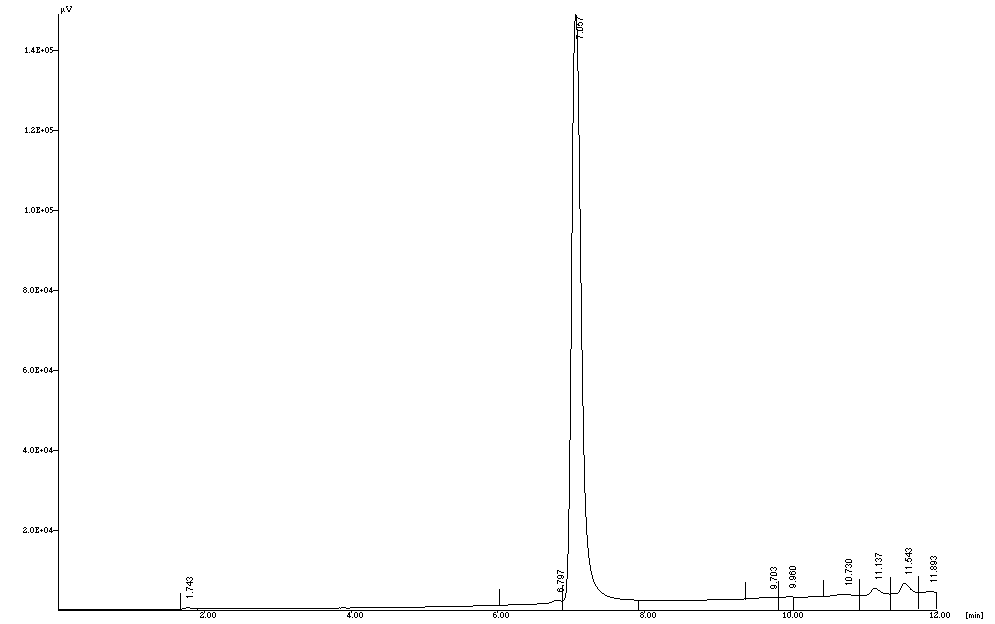

Supplement: Figure S4 — RP-HPLC chromatogram of compound 1. Solvent: 35% v/v aqueous MeOH increasing to 80% in 10 min and subsequently changing back to 35%; flow rate: 1 mL/min, λ = 245 nm (TIF) [file pone.0023922.s004.tif]

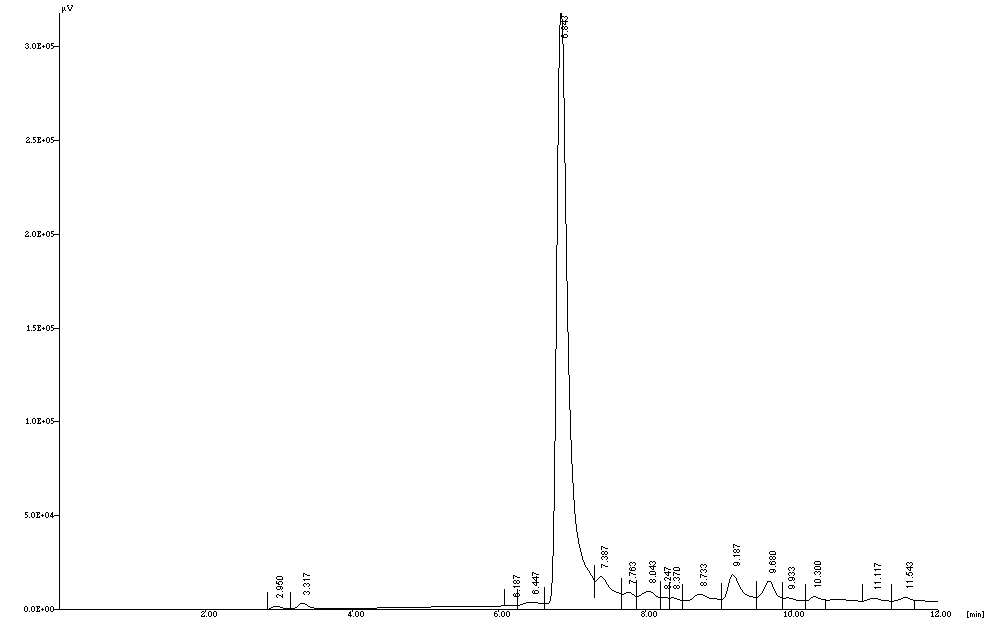

Supplement: Figure S5 — RP-HPLC chromatogram of compound 1″. Solvent: 35% v/v aqueous MeOH increasing to 80% in 10 min and subsequently changing back to 35%; flow rate:1 mL/min, λ = 245 nm (TIF) [file pone.0023922.s005.tif]

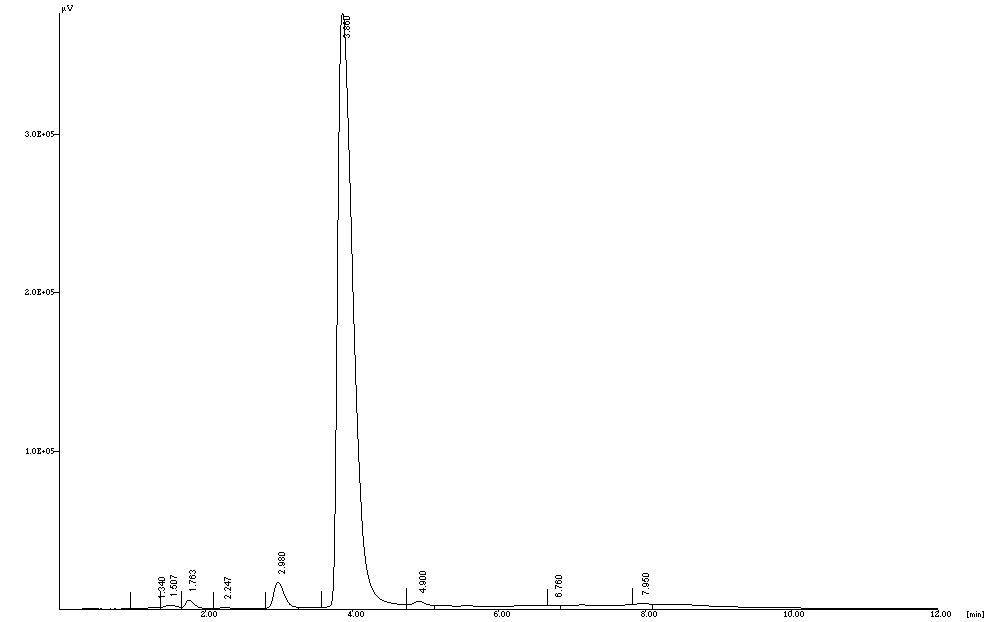

Supplement: Figure S6 — RP-HPLC chromatogram of compound 3. Solvent: 55% v/v aqueous MeOH increasing to 80% in 10 min and subsequently changing back to 55%; flow rate:1 mL/min, λ = 245 nm (TIF) [file pone.0023922.s006.tif]

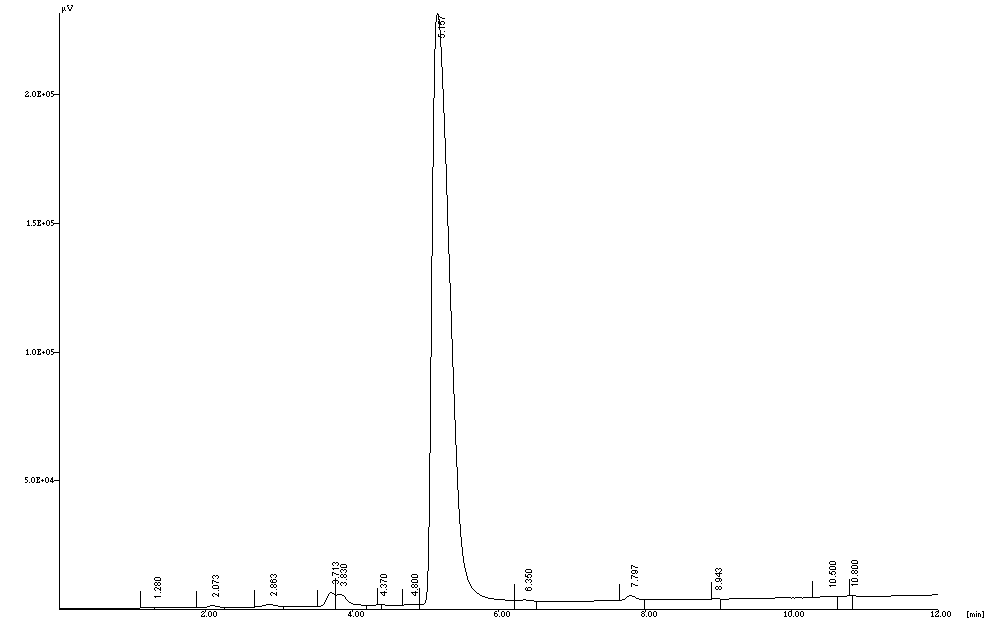

Supplement: Figure S7 — RP-HPLC chromatogram of compound 4. Solvent: 55% v/v aqueous MeOH increasing to 80% in 10 min and subsequently changing back to 55%; flow rate:1 mL/min, λ = 245 nm (TIF) [file pone.0023922.s007.tif]

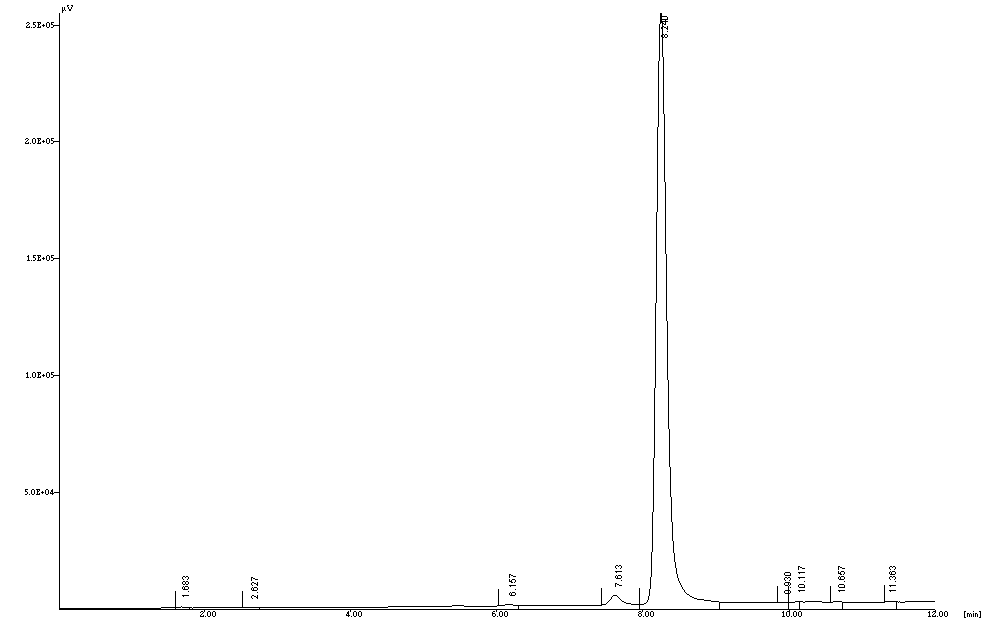

Supplement: Figure S8 — RP-HPLC chromatogram of compound 5. Solvent: 55% v/v aqueous MeOH increasing to 80% in 10 min and subsequently changing back to 55%; flow rate:1 mL/min, λ = 245 nm (TIF) [file pone.0023922.s008.tif]

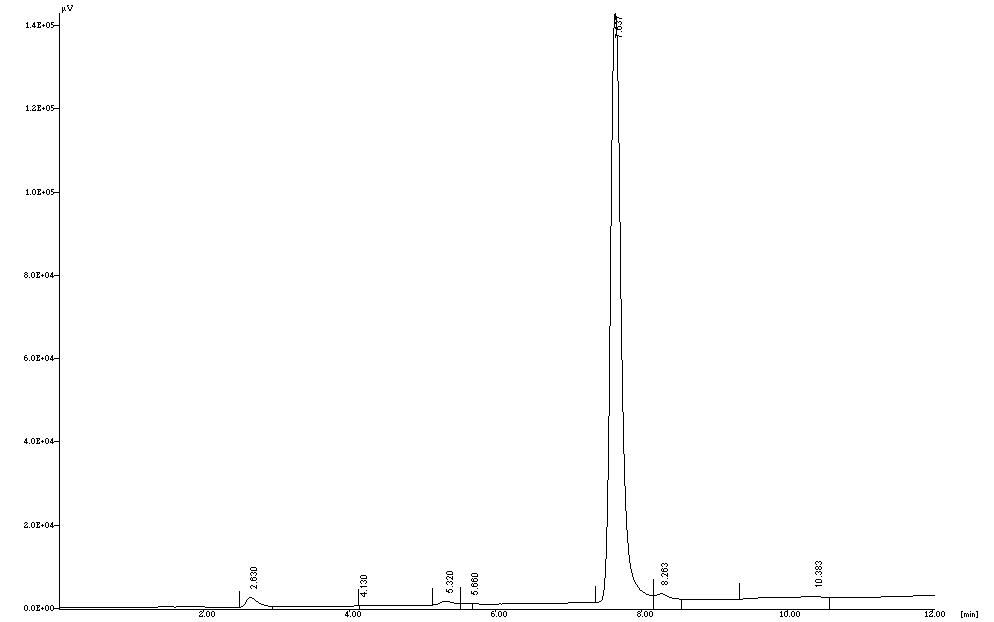

Supplement: Figure S9 — RP-HPLC chromatogram of compound 6. Solvent: 55% v/v aqueous MeOH increasing to 80% in 10 min and subsequently changing back to 55%; flow rate:1 mL/min, λ = 245 nm (TIF) [file pone.0023922.s009.tif]

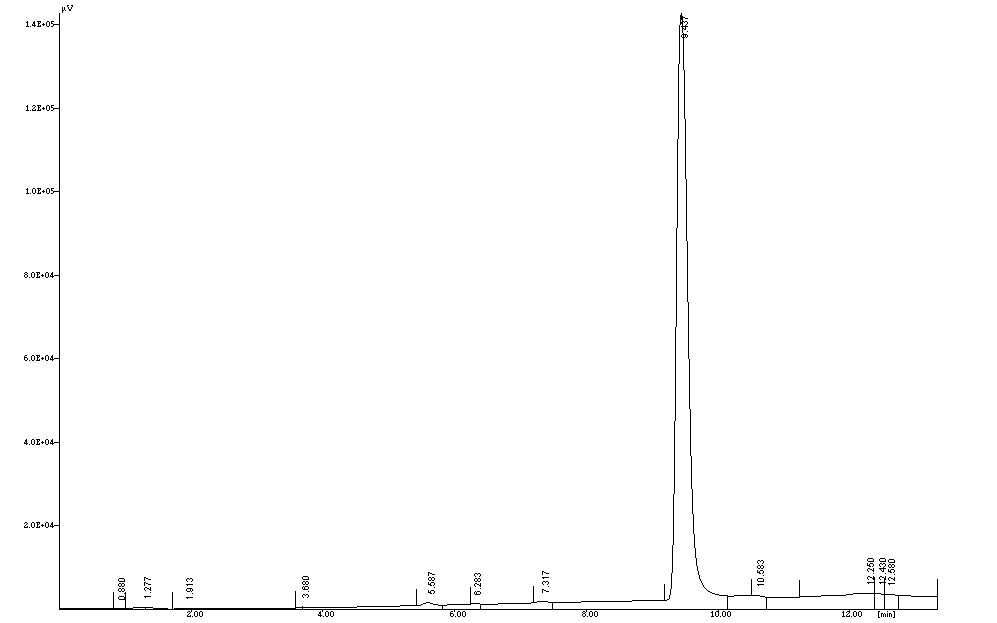

Supplement: Figure S10 — RP-HPLC chromatogram of compound 7. Solvent: 55% v/v aqueous MeOH increasing to 80% in 10 min and subsequently changing back to 55%; flow rate:1 mL/min, λ = 245 nm (TIF) [file pone.0023922.s010.tif]

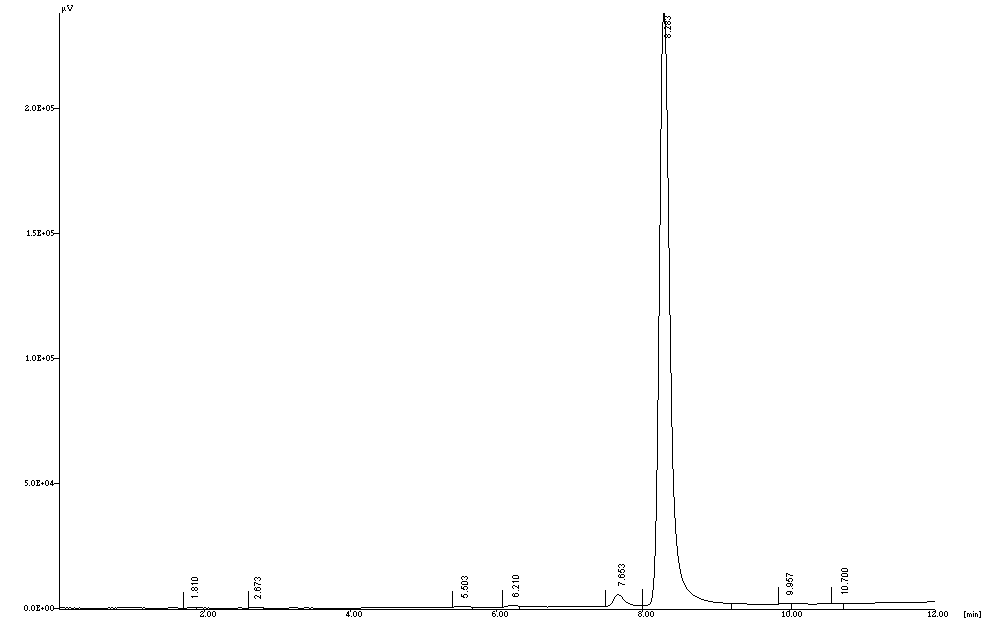

Supplement: Figure S11 — RP-HPLC chromatogram of compound 8. Solvent: 55% v/v aqueous MeOH increasing to 80% in 10 min and subsequently changing back to 55%; flow rate:1 mL/min, λ = 245 nm (TIF) [file pone.0023922.s011.tif]

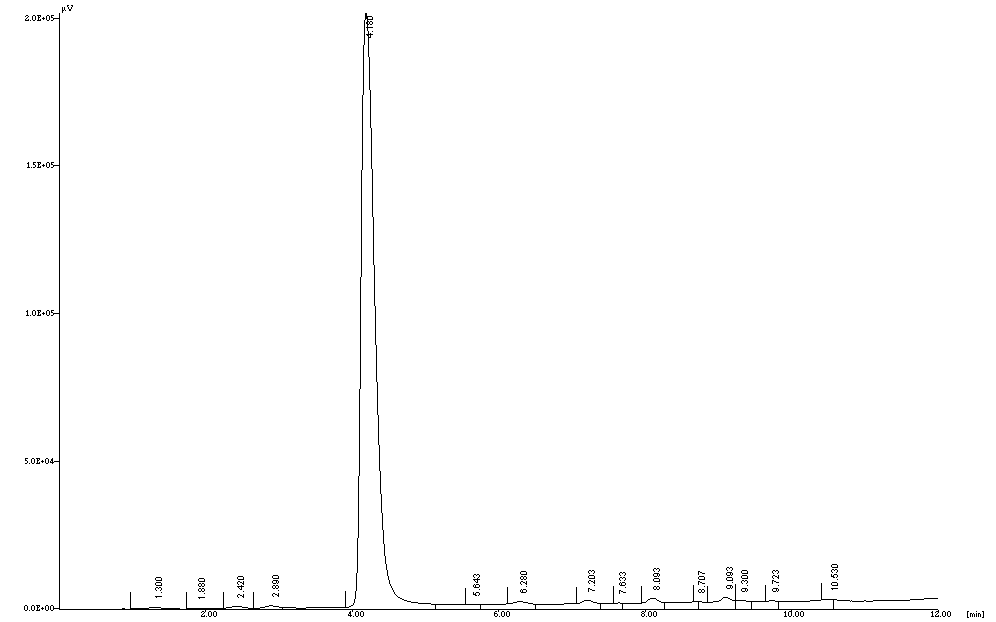

Supplement: Figure S12 — RP-HPLC chromatogram of compound 9. Solvent: 55% v/v aqueous MeOH increasing to 80% in 10 min and subsequently changing back to 55%; flow rate:1 mL/min, λ = 245 nm (TIF) [file pone.0023922.s012.tif]

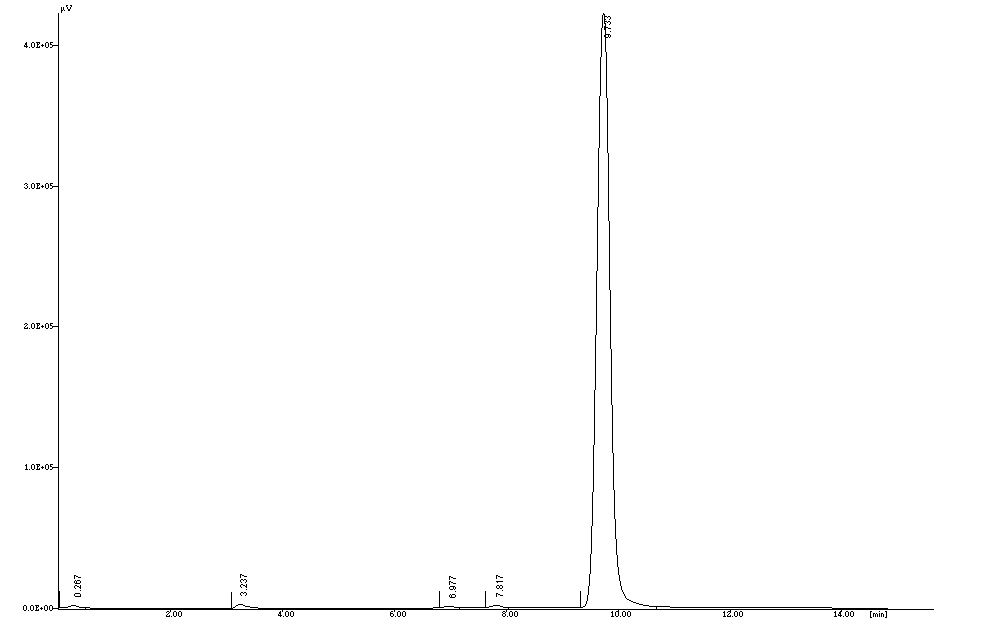

Supplement: Figure S13 — NP-HPLC chromatogram of compound 11. Solvent: 5% v/v isopropanol in dichloromethane increasing to 17% in 12 min; flow rate:1 mL/min, λ = 245 nm (TIF) [file pone.0023922.s013.tif]

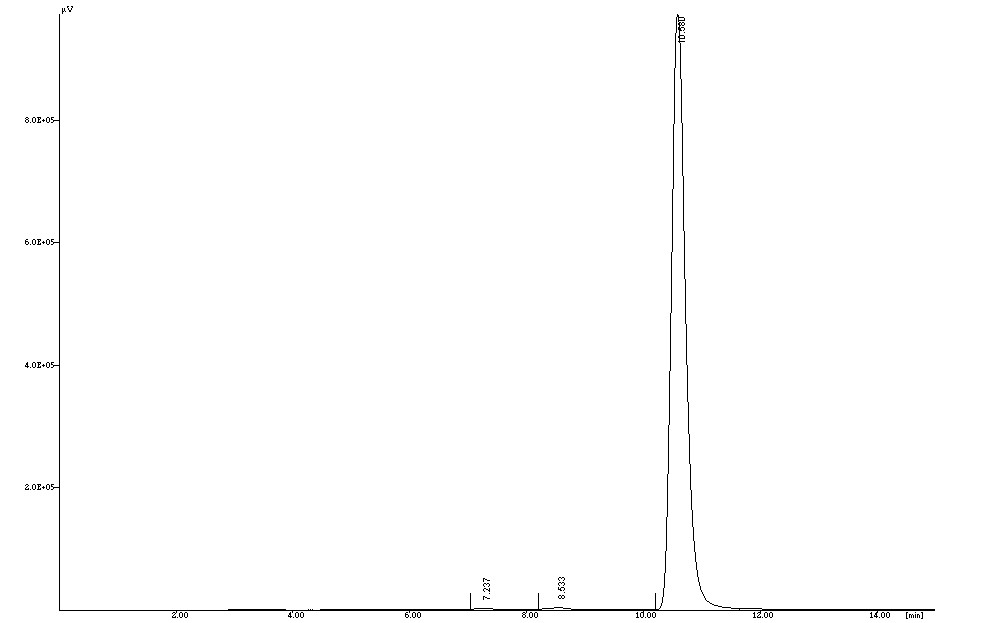

Supplement: Figure S14 — NP-HPLC chromatogram of compound 12. Solvent: 5% v/v isopropanol in dichloromethane increasing to 17% in 12 min; flow rate:1 mL/min, λ = 245 nm (TIF) [file pone.0023922.s014.tif]

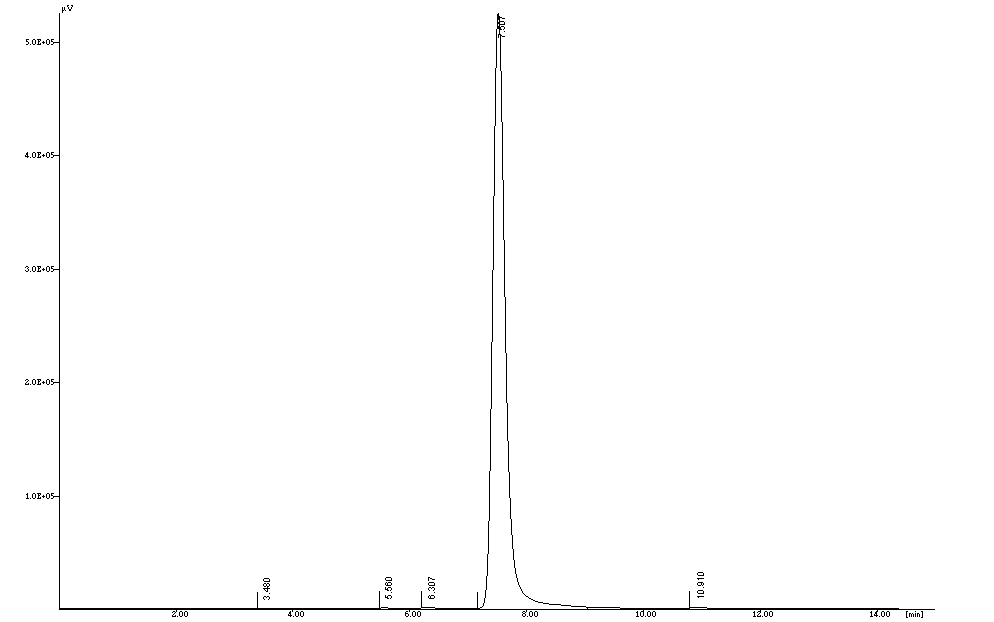

Supplement: Figure S15 — NP-HPLC chromatogram of compound 13. Solvent: 5% v/v isopropanol in dichloromethane increasing to 17% in 12 min; flow rate:1 mL/min, λ = 245 nm (TIF) [file pone.0023922.s015.tif]

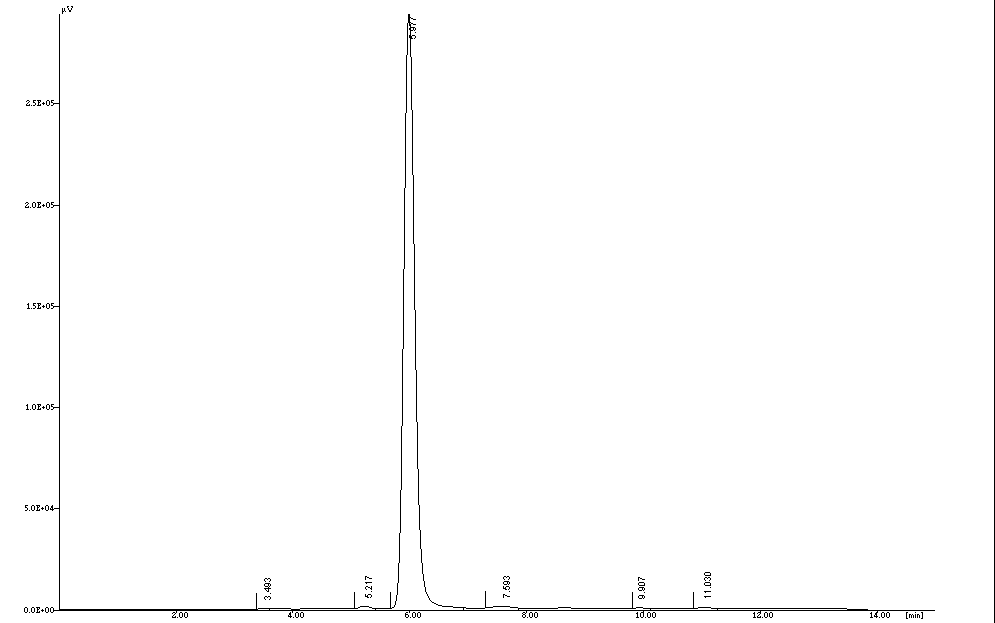

Supplement: Figure S16 — NP-HPLC chromatogram of compound 14. Solvent: 5% v/v isopropanol in dichloromethane increasing to 17% in 12 min; flow rate:1 mL/min, λ = 245 nm (TIF) [file pone.0023922.s016.tif]

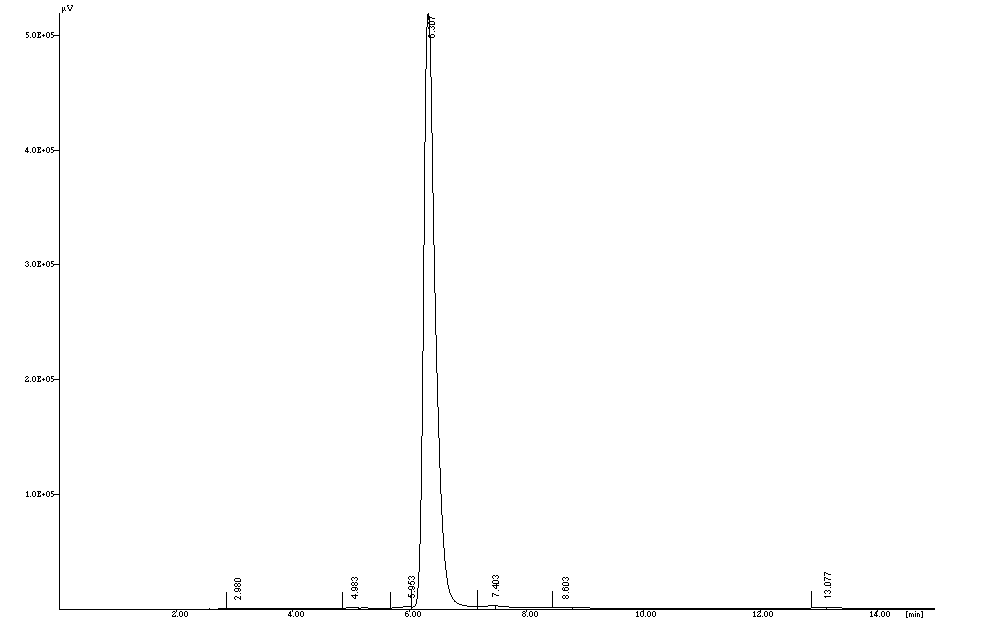

Supplement: Figure S17 — NP-HPLC chromatogram of compound 15. Solvent: 5% v/v isopropanol in dichloromethane increasing to 17% in 12 min; flow rate:1 mL/min, λ = 245 nm (TIF) [file pone.0023922.s017.tif]

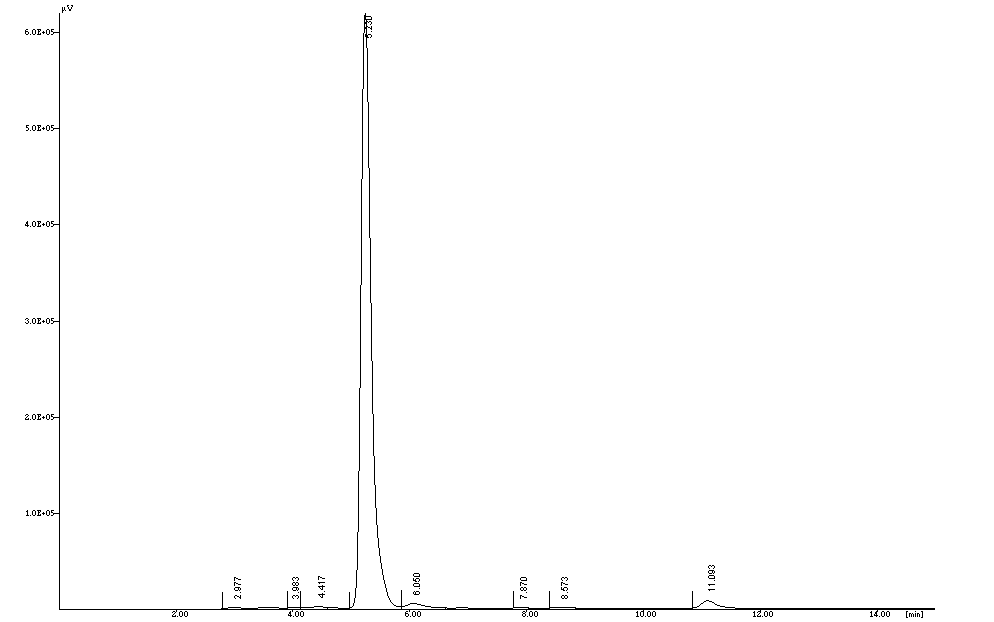

Supplement: Figure S18 — NP-HPLC chromatogram of compound 16. Solvent: 5% v/v isopropanol in dichloromethane increasing to 17% in 12 min; flow rate:1 mL/min, λ = 245 nm (TIF) [file pone.0023922.s018.tif]

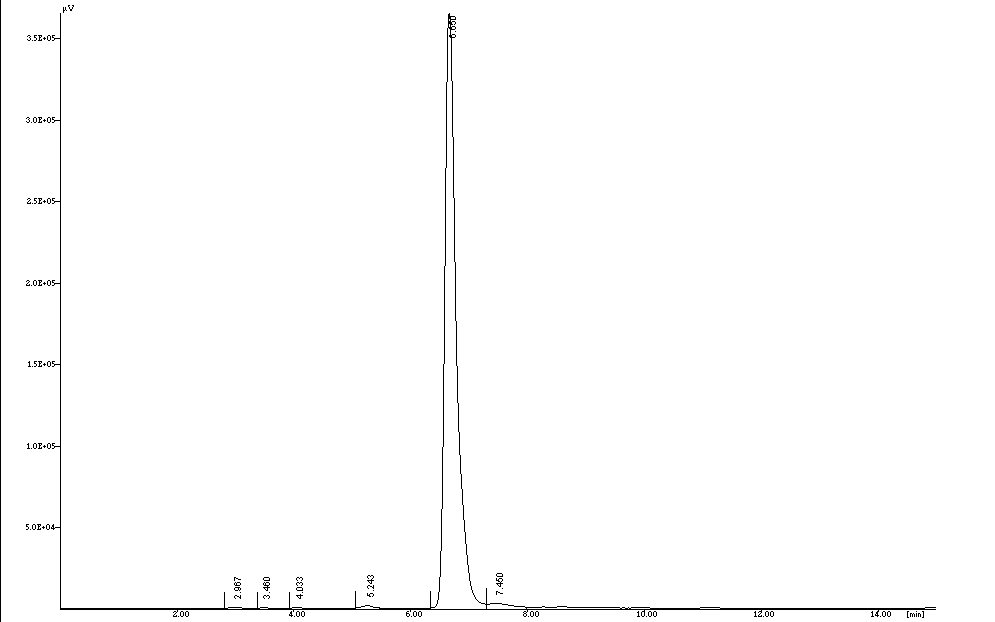

Supplement: Figure S19 — NP-HPLC chromatogram of compound 17. Solvent: 5% v/v isopropanol in dichloromethane increasing to 17% in 12 min; flow rate:1 mL/min, λ = 245 nm (TIF) [file pone.0023922.s019.tif]

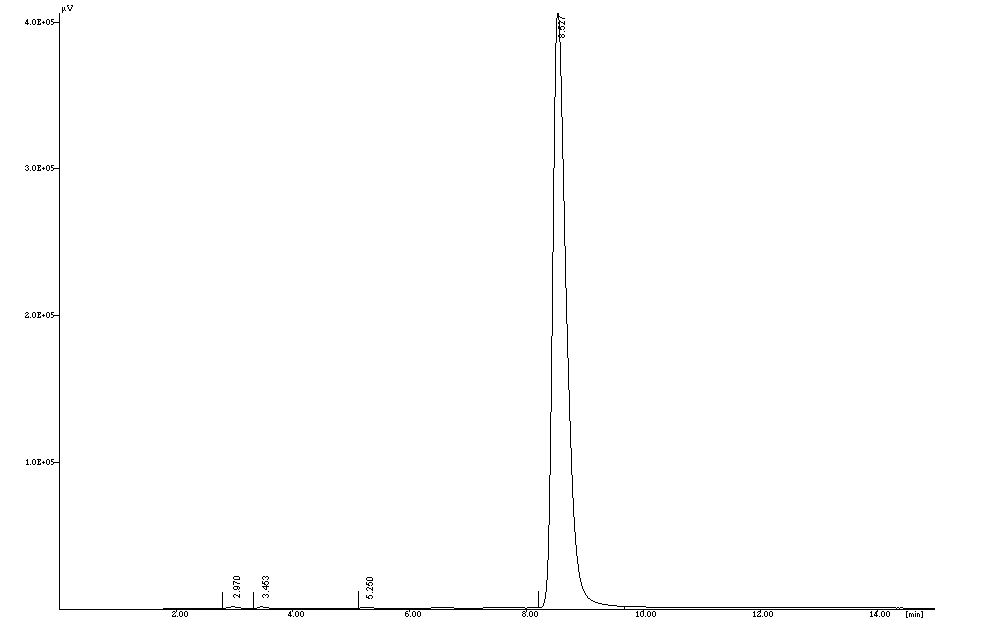

Supplement: Figure S20 — NP-HPLC chromatogram of compound 18. Solvent: 5% v/v isopropanol in dichloromethane increasing to 17% in 12 min; flow rate:1 mL/min, λ = 245 nm (TIF) [file pone.0023922.s020.tif]

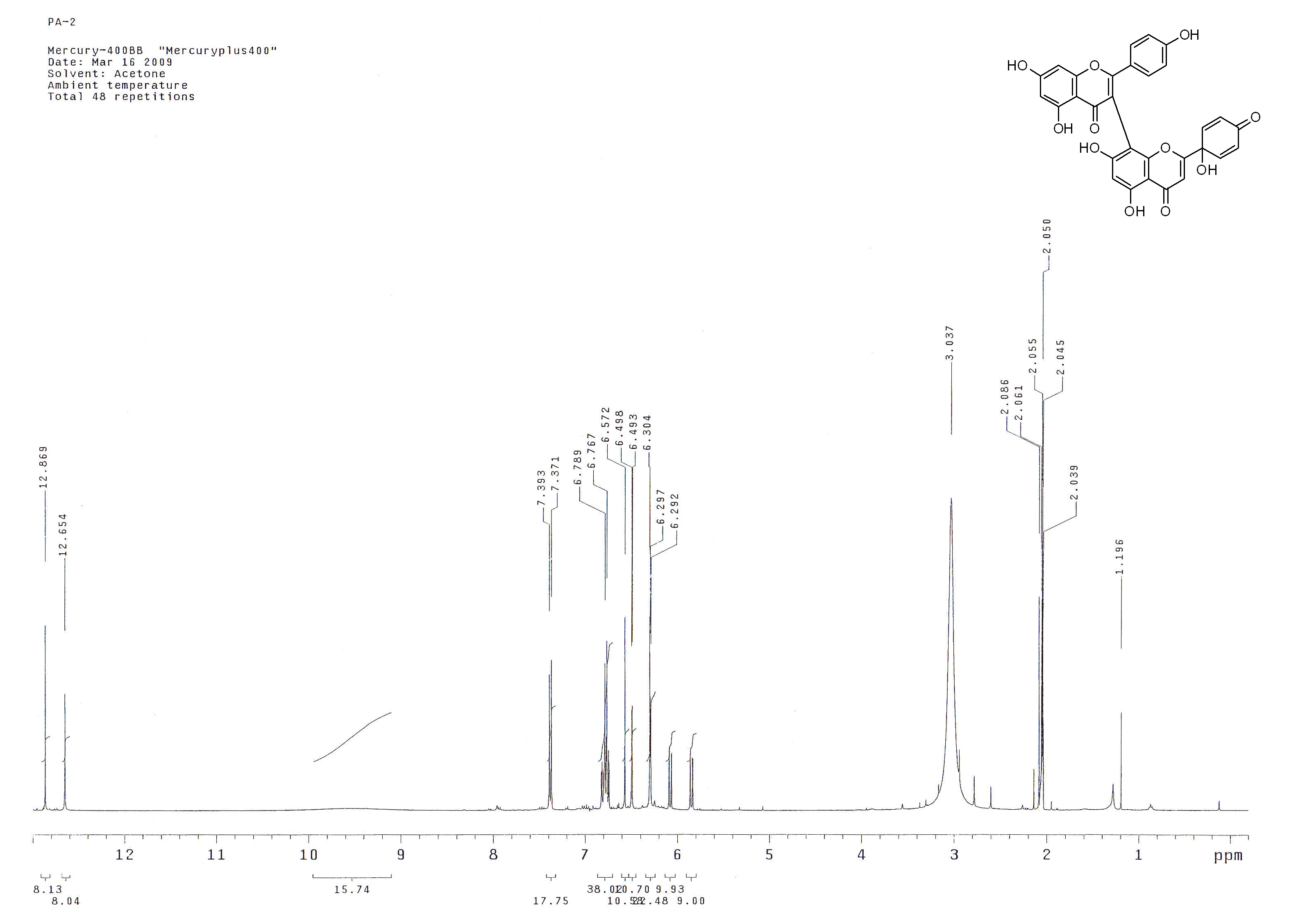

Supplement: Figure S21 — 400 MHz 1H NMR spectrum of compound 1″. (TIF) [file pone.0023922.s021.tif]

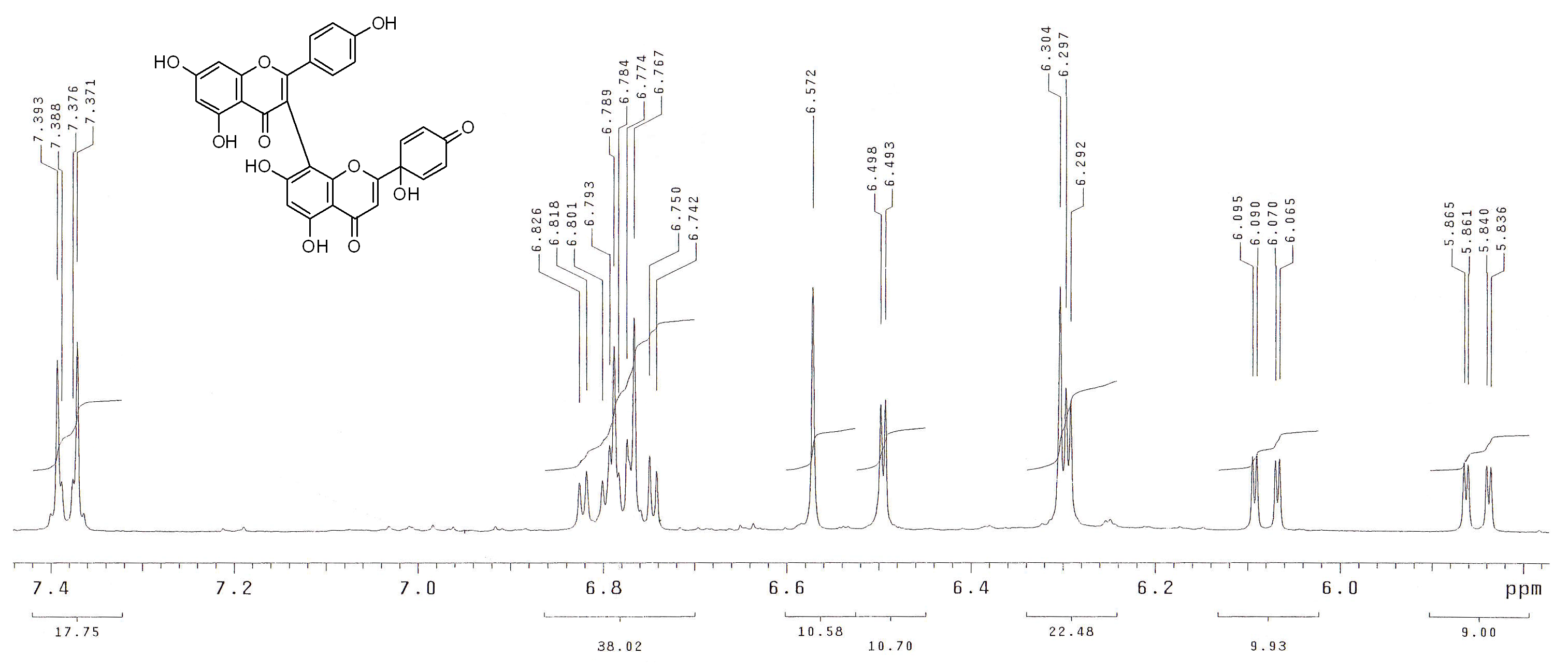

Supplement: Figure S22 — Zoom of 400 MHz 1H NMR spectrum of compound 1″. (TIF) [file pone.0023922.s022.tif]

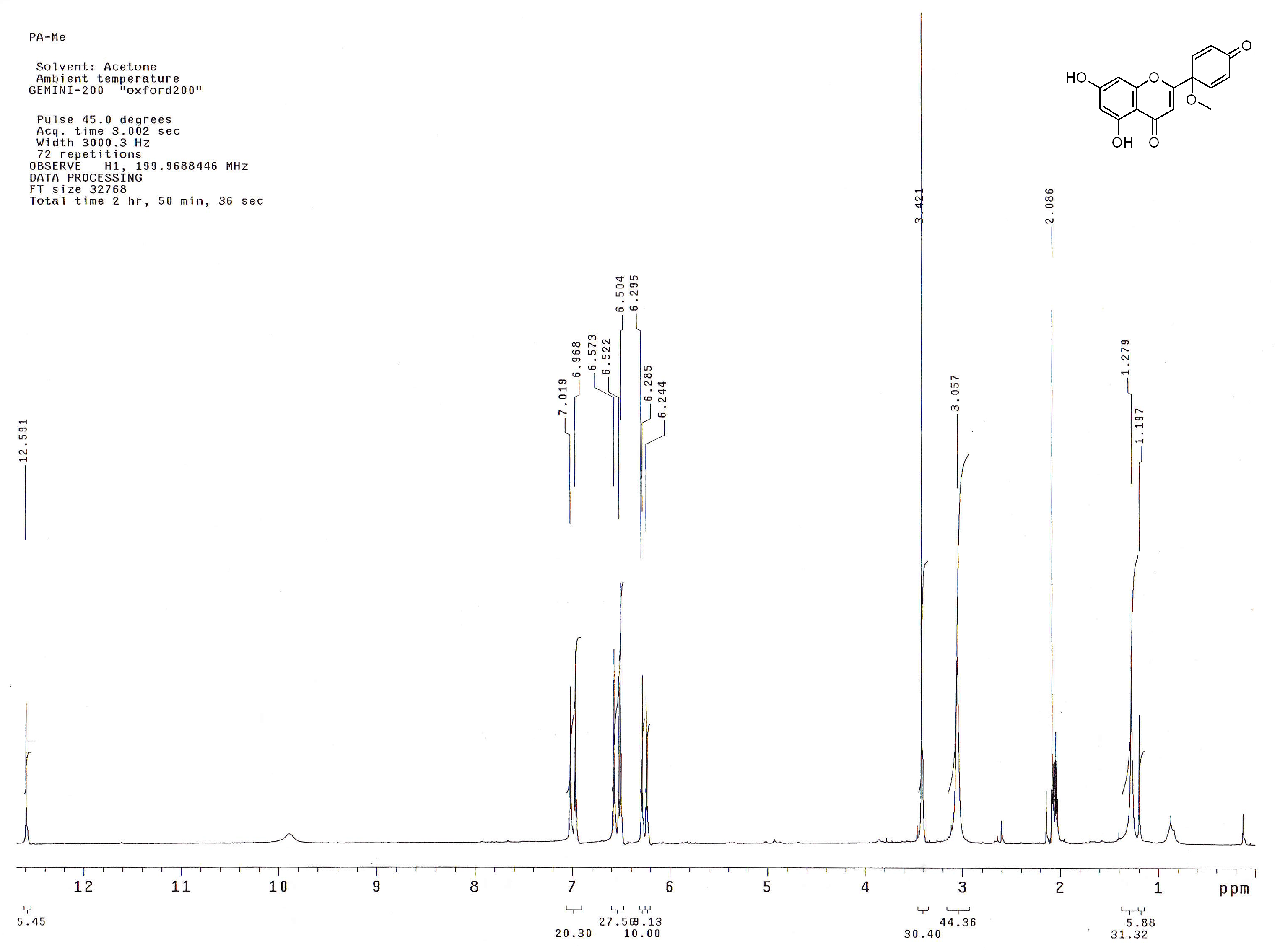

Supplement: Figure S23 — 200 MHz 1H NMR spectrum of compound 3 before crystallization. (TIF) [file pone.0023922.s023.tif]

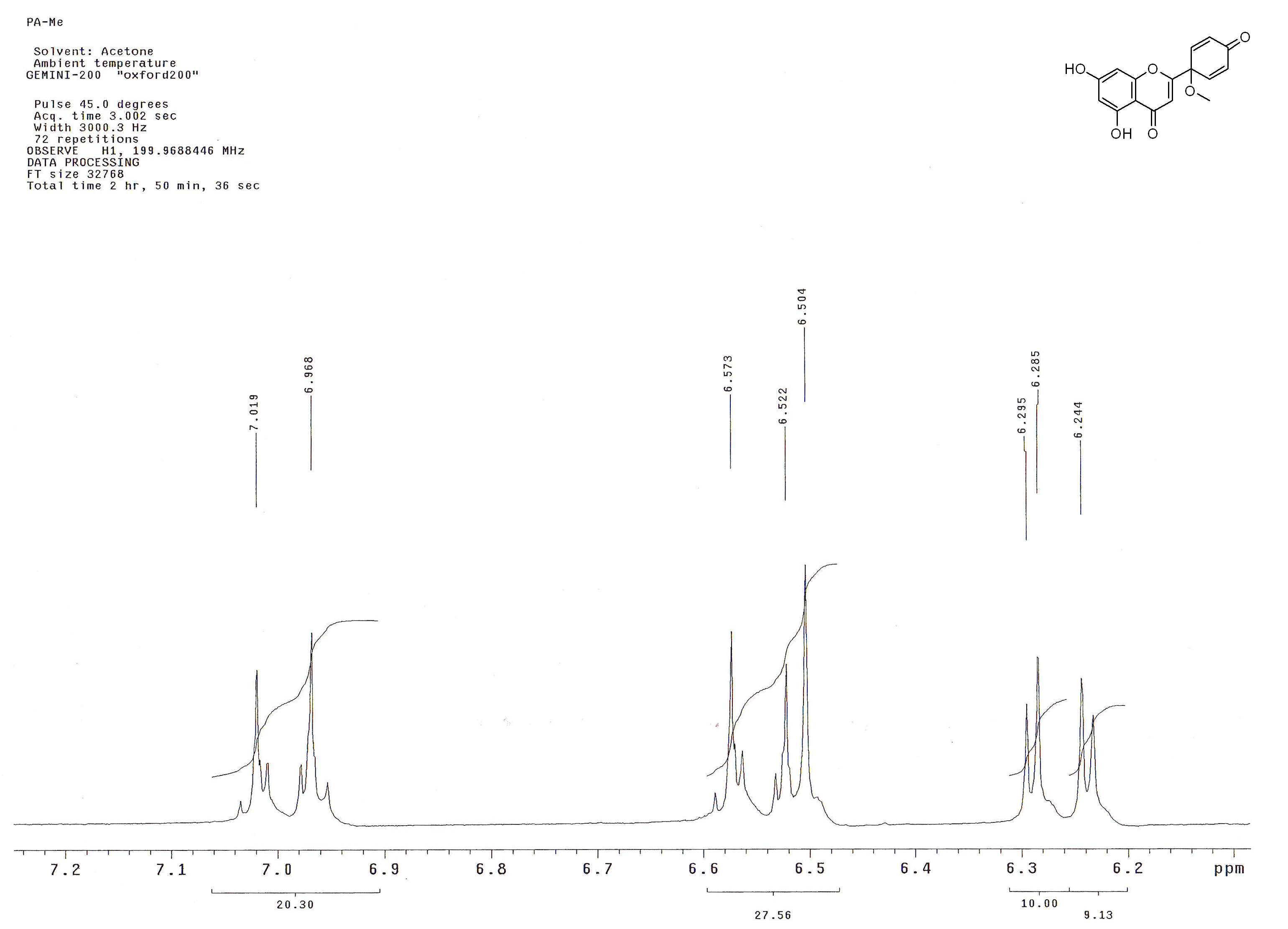

Supplement: Figure S24 — Zoom of 200 MHz 1H NMR spectrum of compound 3 before crystallization. (TIF) [file pone.0023922.s024.tif]

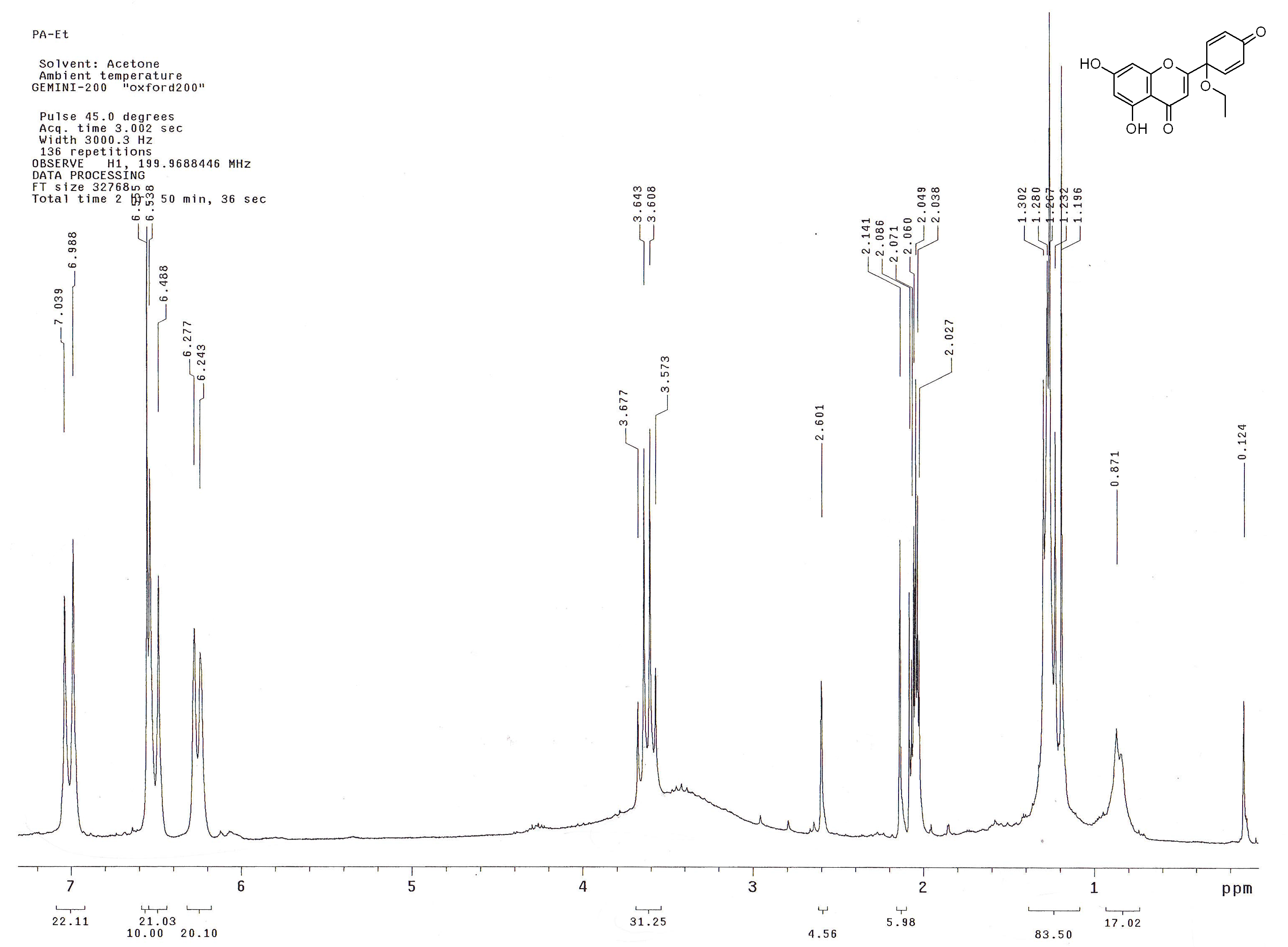

Supplement: Figure S25 — 200 MHz 1H NMR spectrum of compound 4 before crystallization. (TIF) [file pone.0023922.s025.tif]

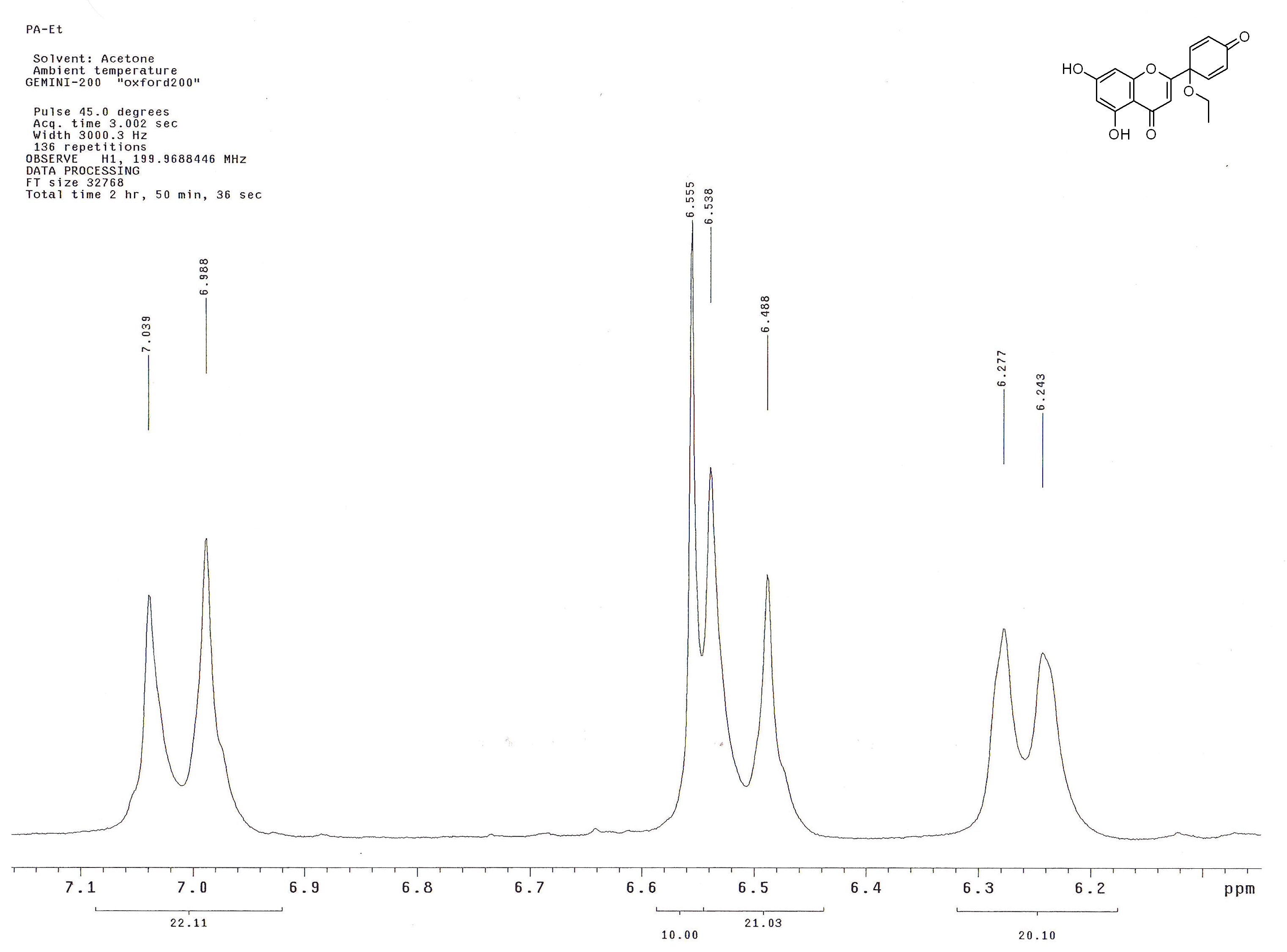

Supplement: Figure S26 — Zoom of 200 MHz 1H NMR spectrum of compound 4 before crystallization. (TIF) [file pone.0023922.s026.tif]

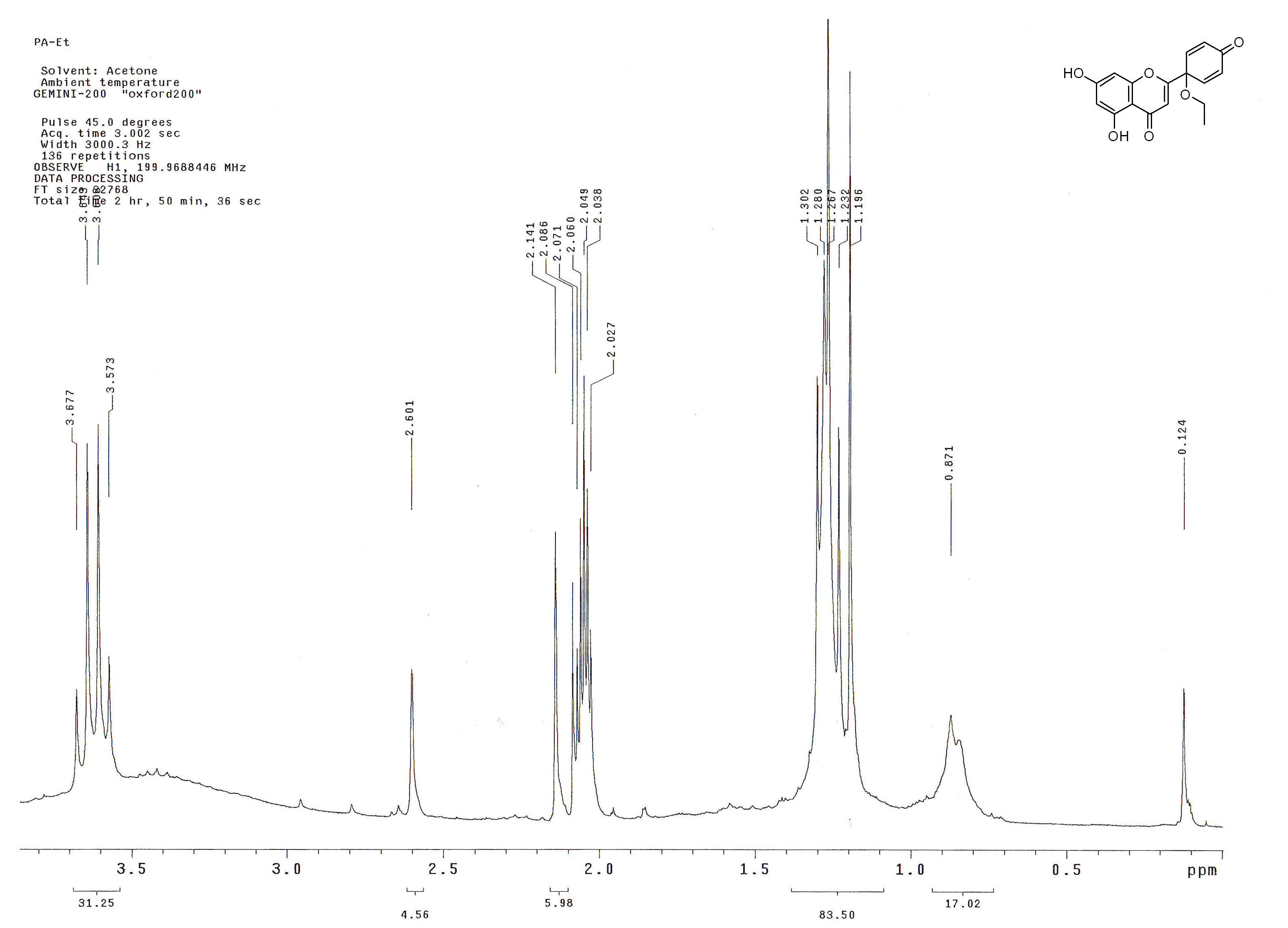

Supplement: Figure S27 — Zoom of 200 MHz 1H NMR spectrum of compound 4 before crystallization. (TIF) [file pone.0023922.s027.tif]

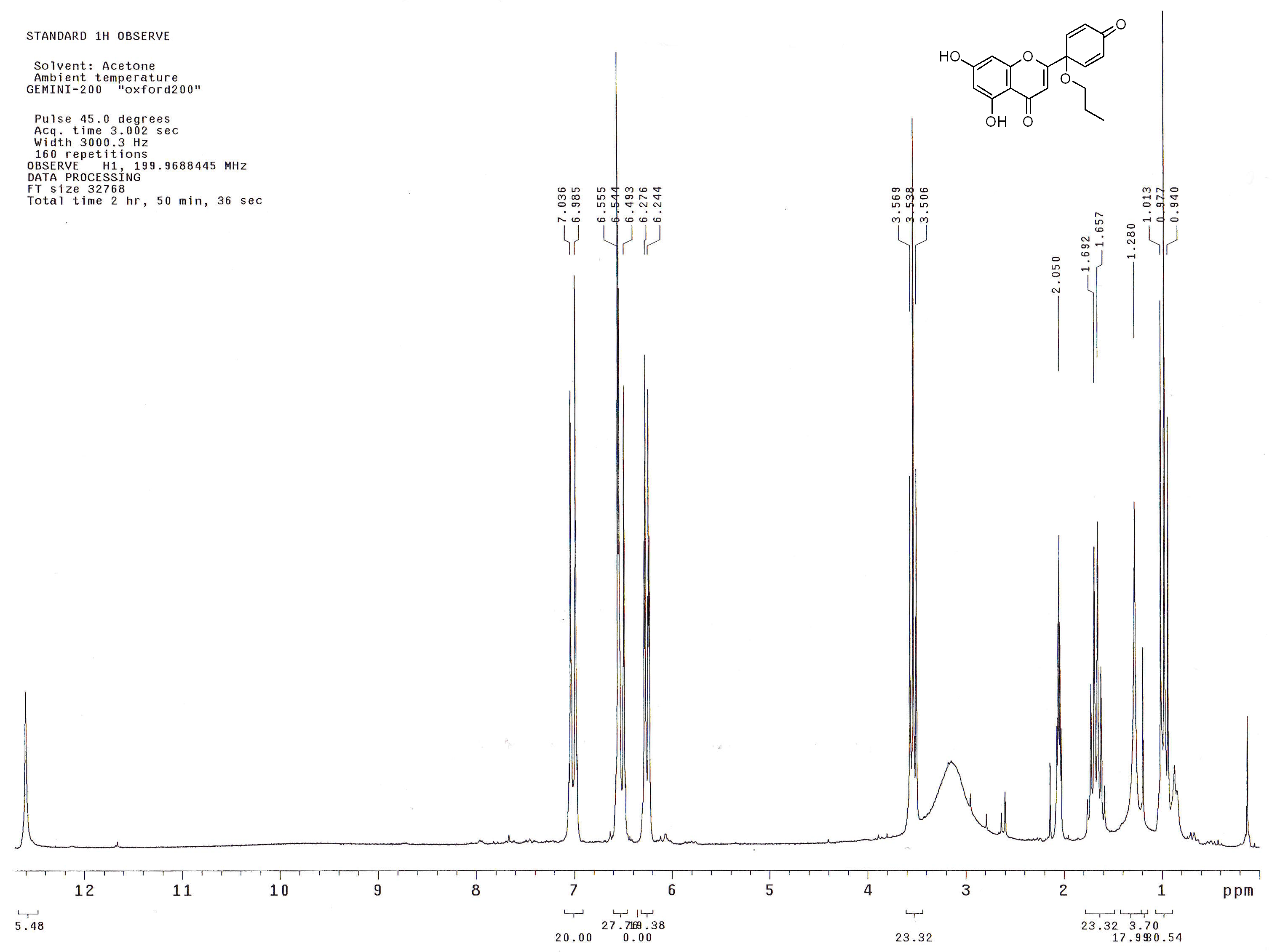

Supplement: Figure S28 — 200 MHz 1H NMR spectrum of compound 5 before crystallization. (TIF) [file pone.0023922.s028.tif]

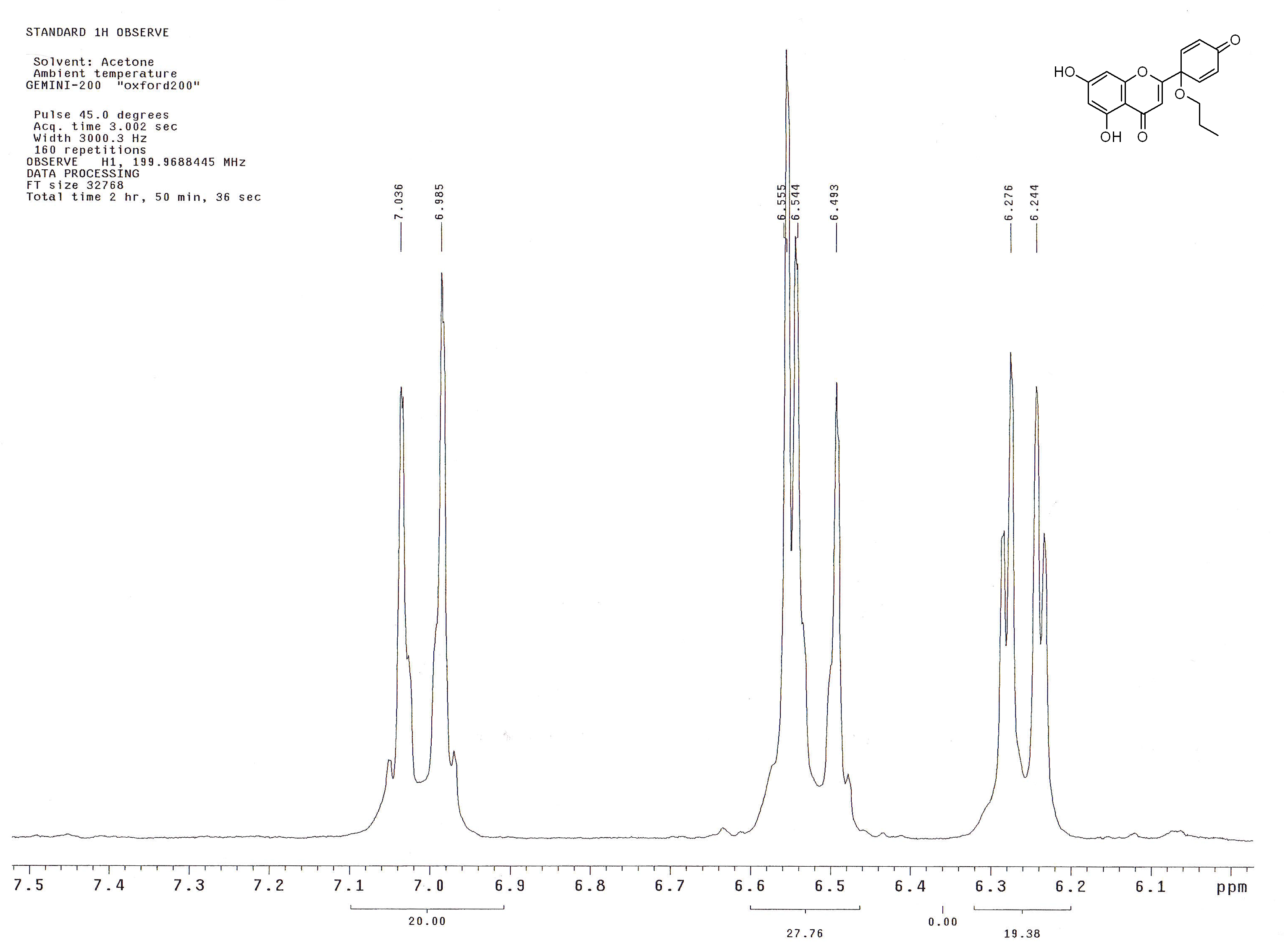

Supplement: Figure S29 — Zoom of 200 MHz 1H NMR spectrum of compound 5 before crystallization. (TIF) [file pone.0023922.s029.tif]

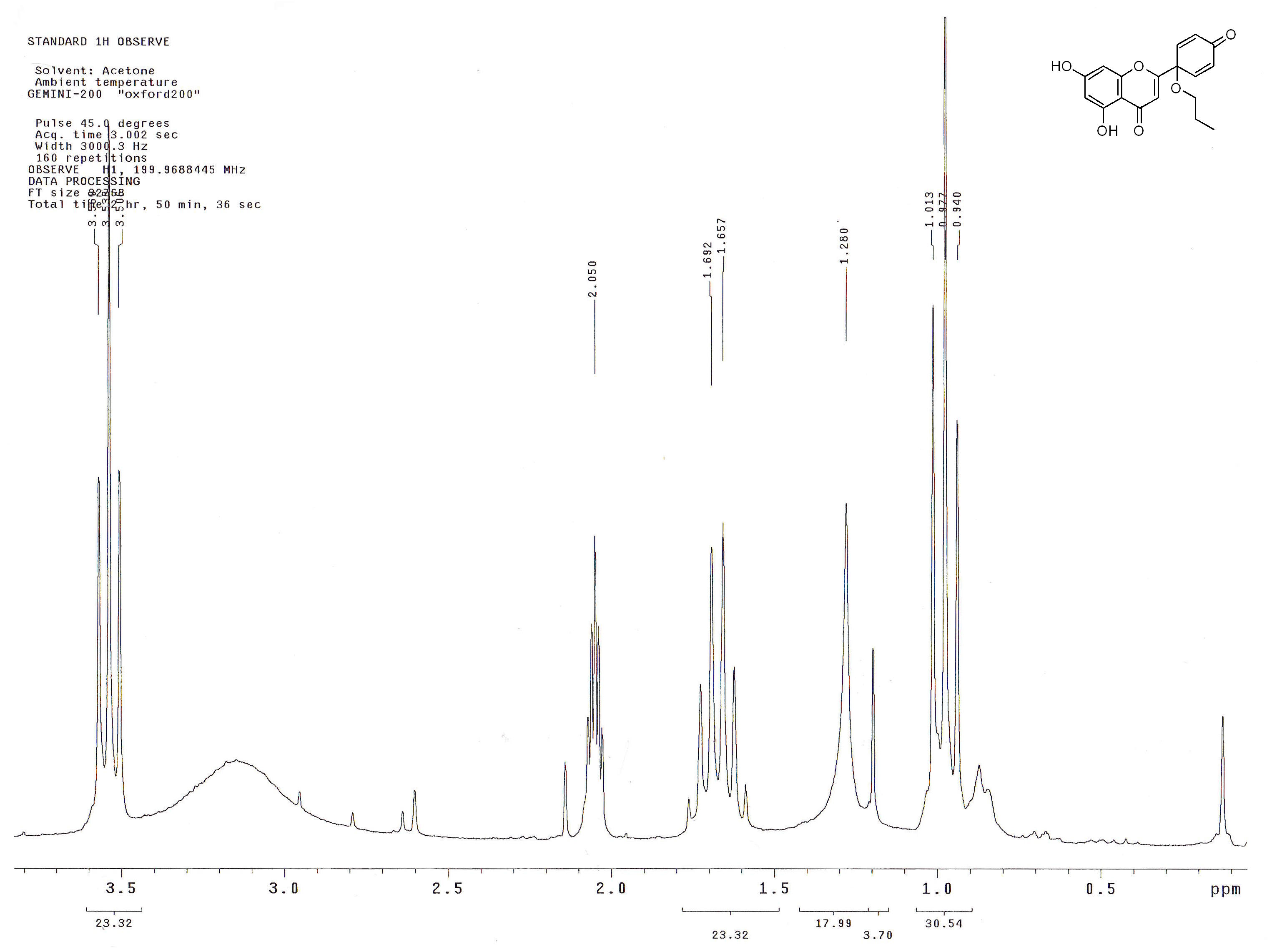

Supplement: Figure S30 — Zoom of 200 MHz 1H NMR spectrum of compound 5 before crystallization. (TIF) [file pone.0023922.s030.tif]

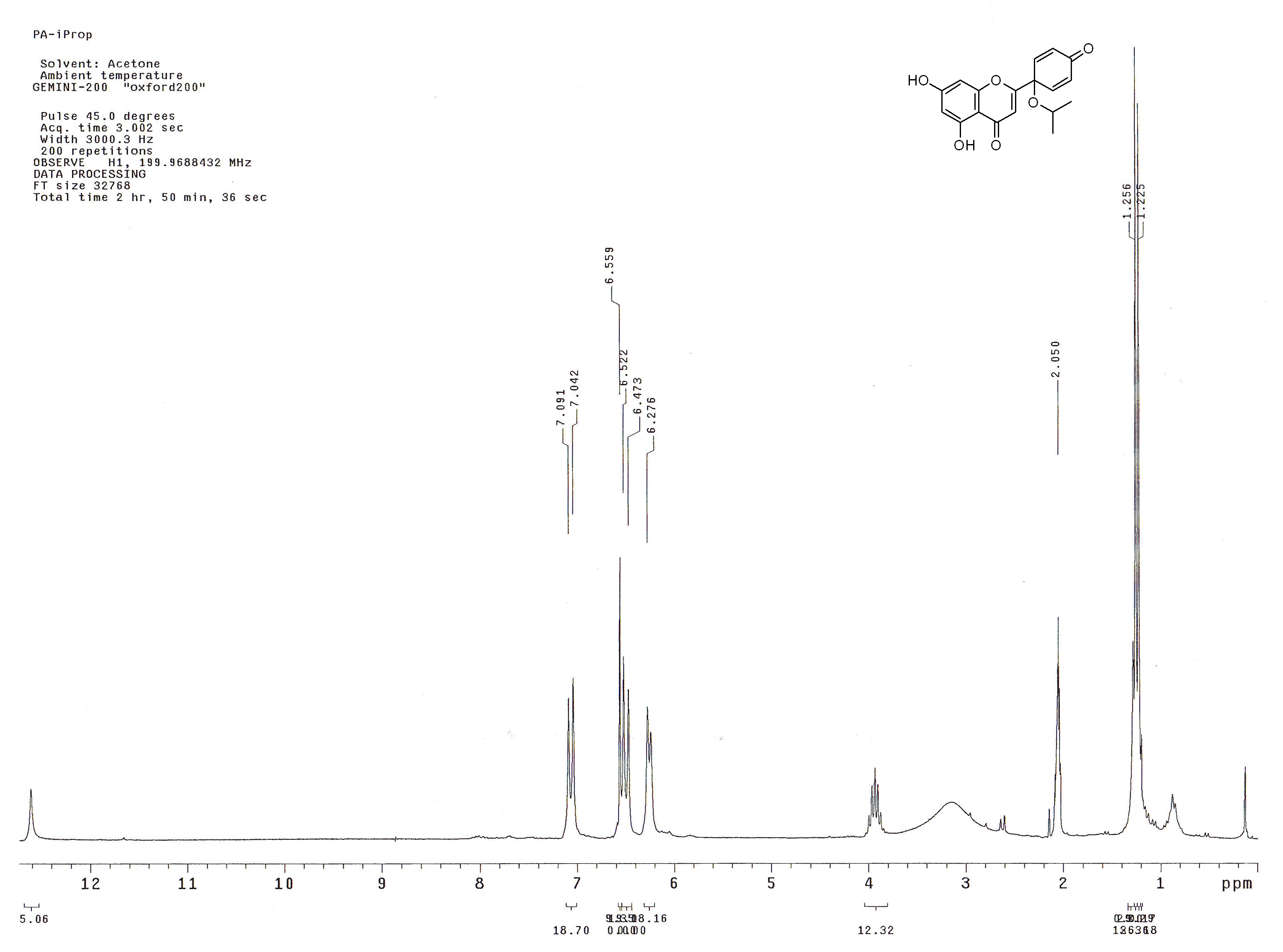

Supplement: Figure S31 — 200 MHz 1H NMR spectrum of compound 6 before crystallization. (TIF) [file pone.0023922.s031.tif]

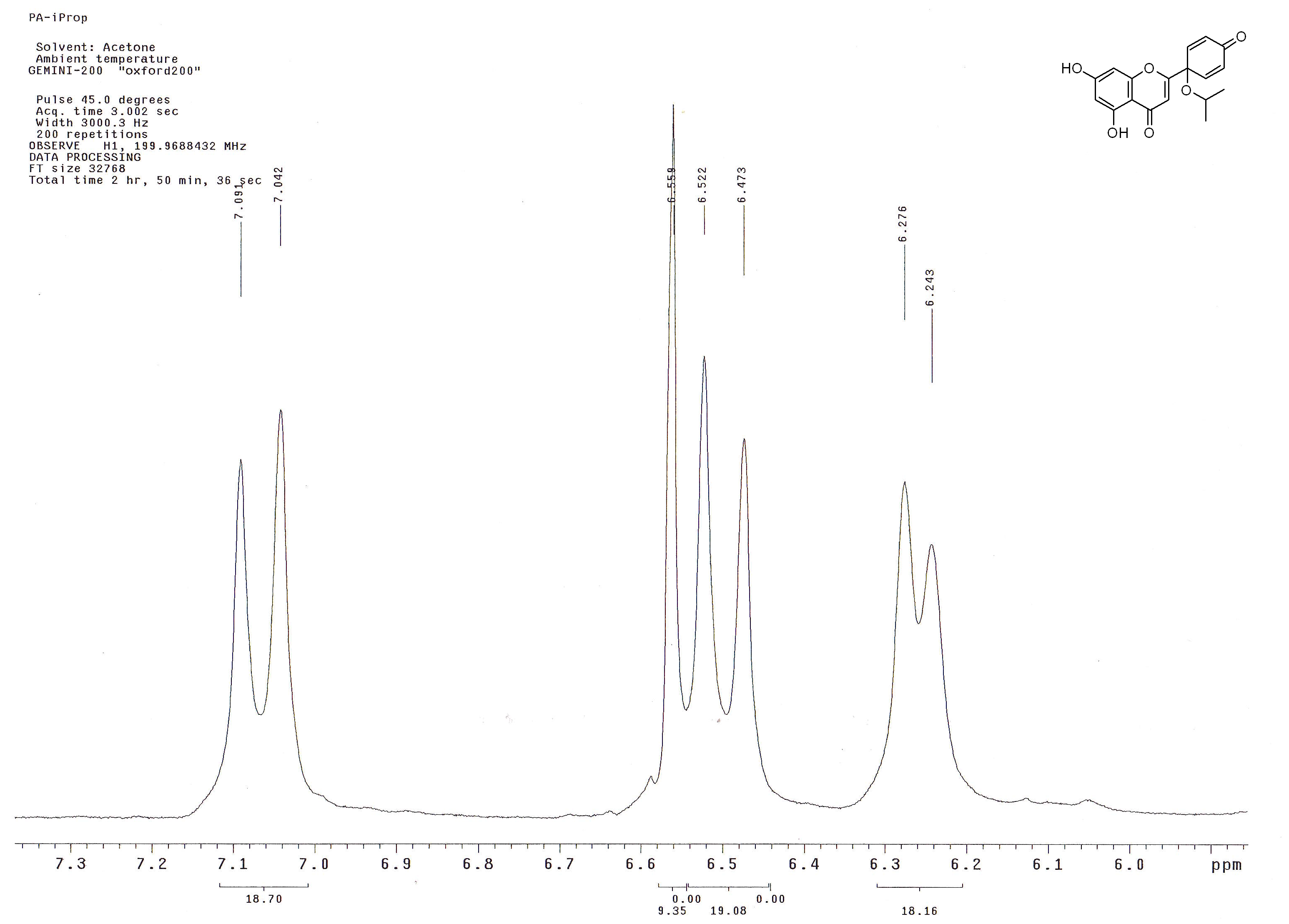

Supplement: Figure S32 — Zoom of 200 MHz 1H NMR spectrum of compound 6 before crystallization. (TIF) [file pone.0023922.s032.tif]

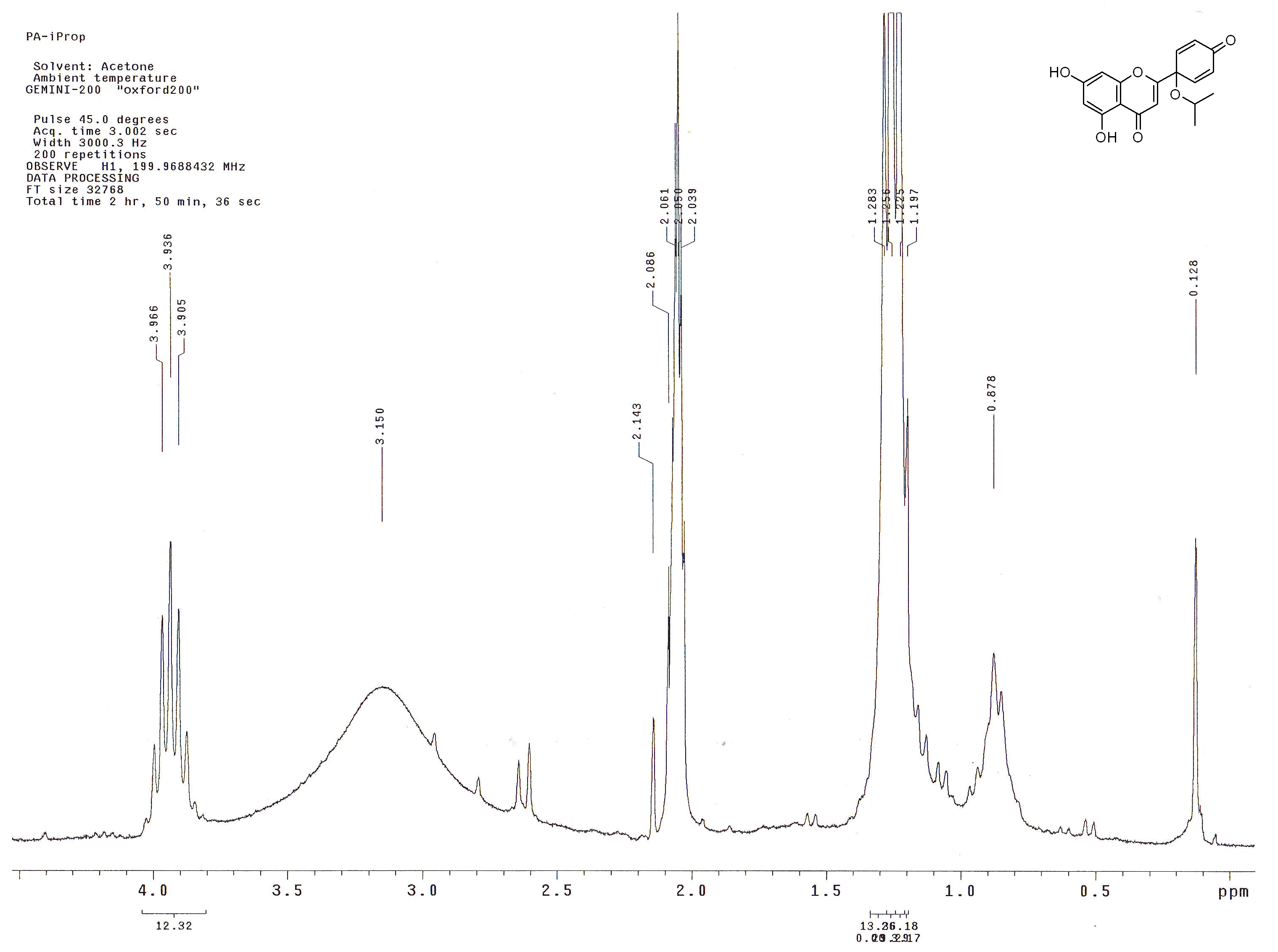

Supplement: Figure S33 — Zoom of 200 MHz 1H NMR spectrum of compound 6 before crystallization. (TIF) [file pone.0023922.s033.tif]

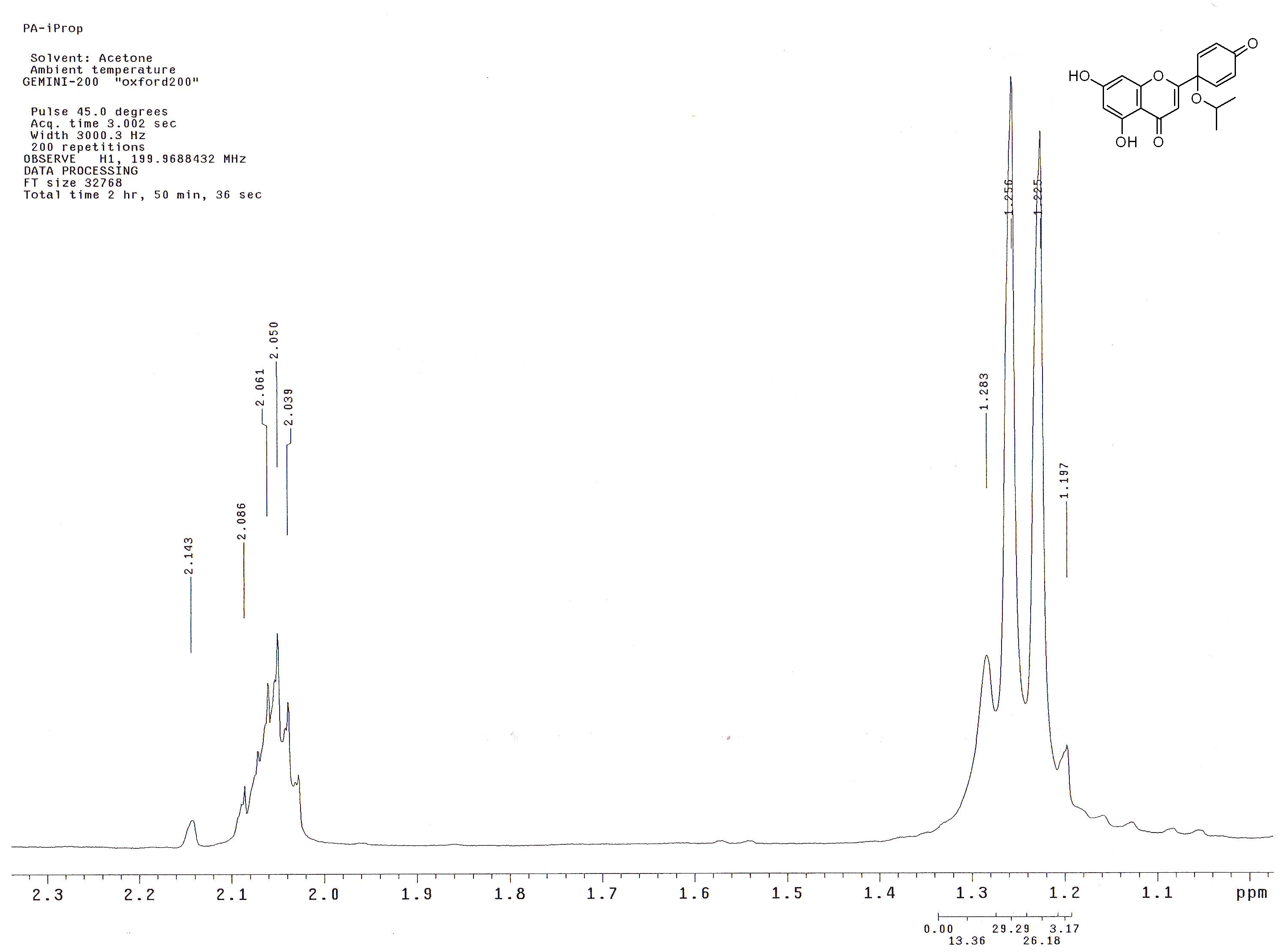

Supplement: Figure S34 — Zoom of 200 MHz 1H NMR spectrum of compound 6 before crystallization. (TIF) [file pone.0023922.s034.tif]

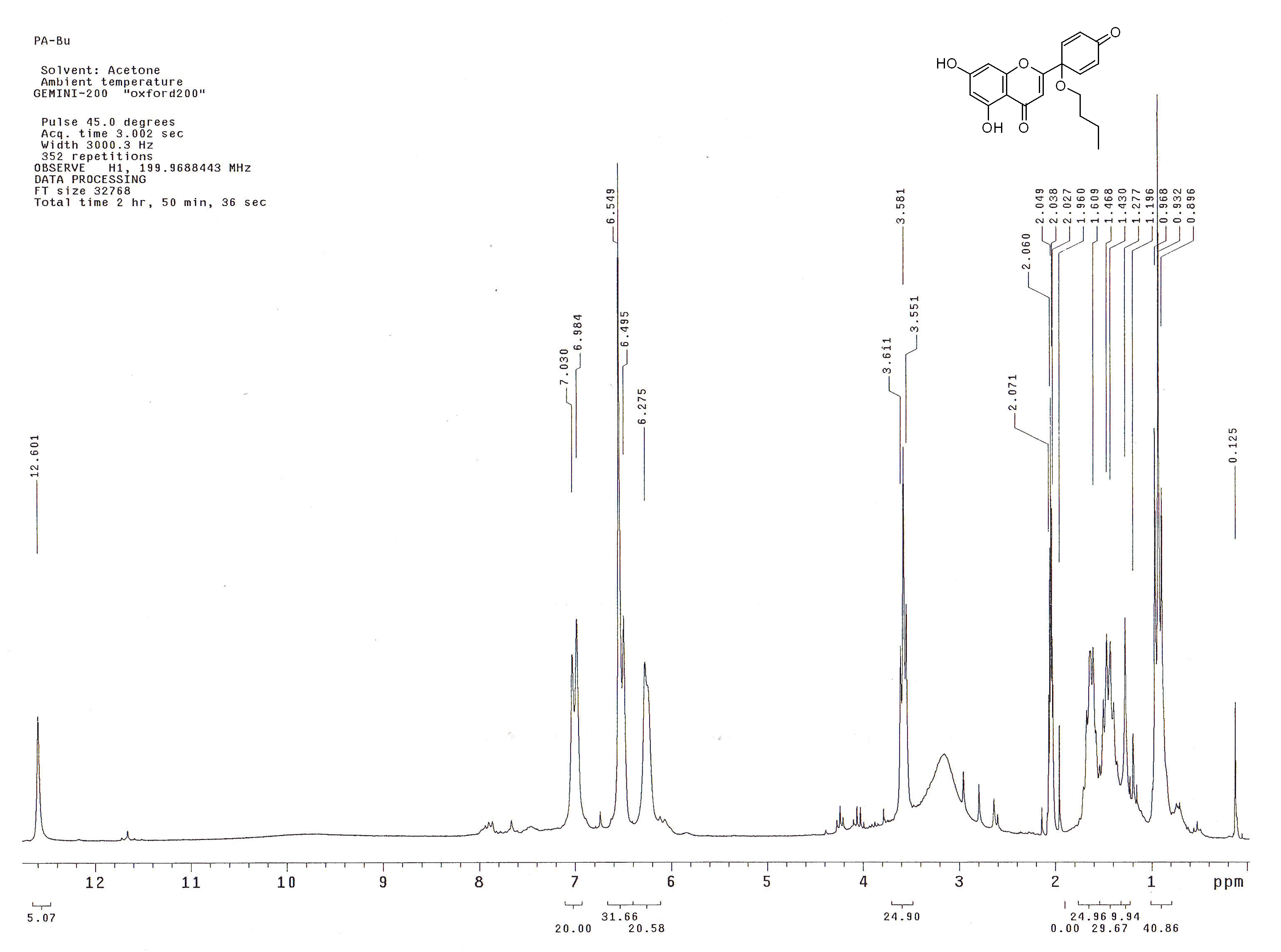

Supplement: Figure S35 — 200 MHz 1H NMR spectrum of compound 7 before crystallization. (TIF) [file pone.0023922.s035.tif]

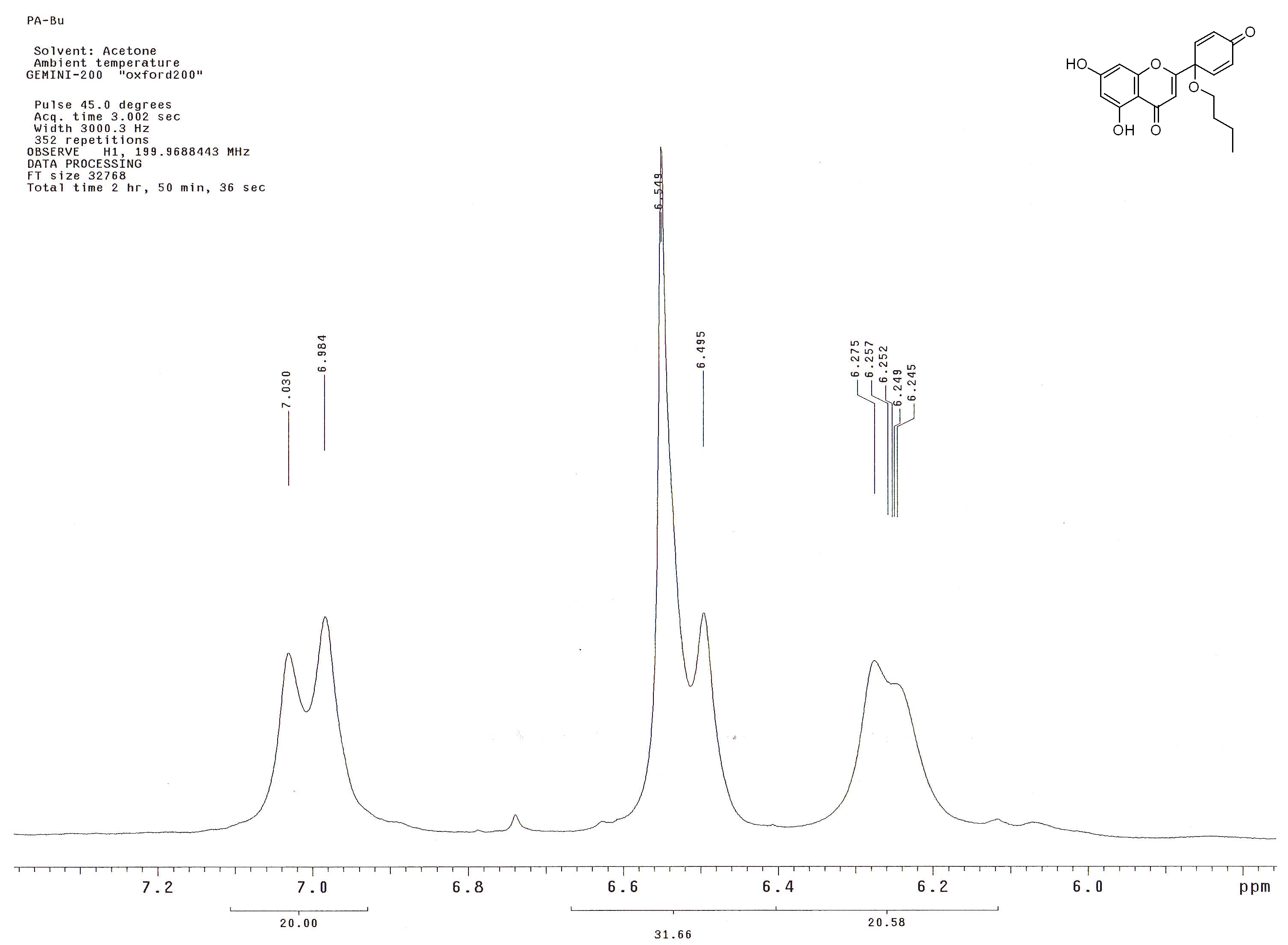

Supplement: Figure S36 — Zoom of 200 MHz 1H NMR spectrum of compound 7 before crystallization. (TIF) [file pone.0023922.s036.tif]

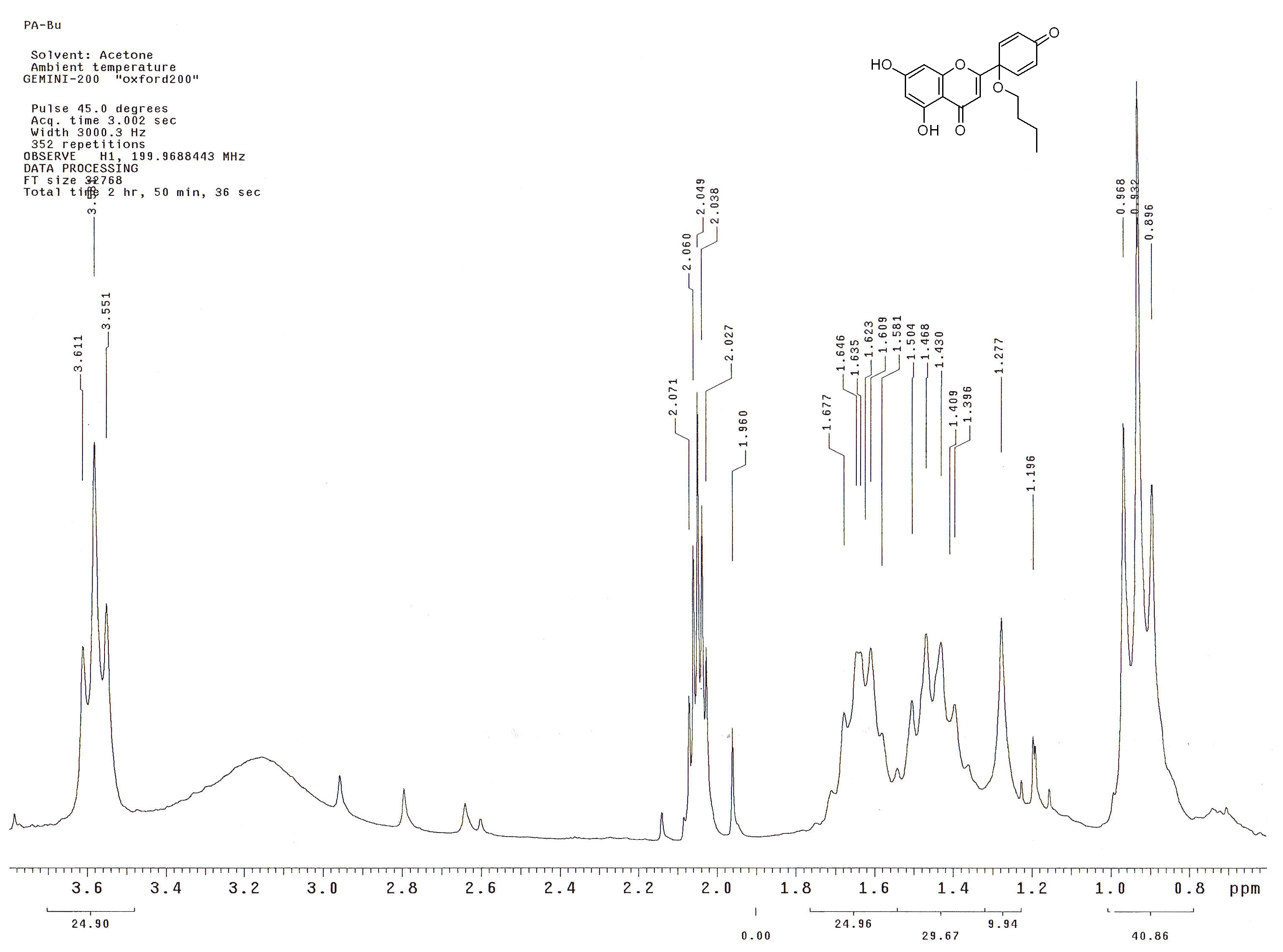

Supplement: Figure S37 — Zoom of 200 MHz 1H NMR spectrum of compound 7 before crystallization. (TIF) [file pone.0023922.s037.tif]

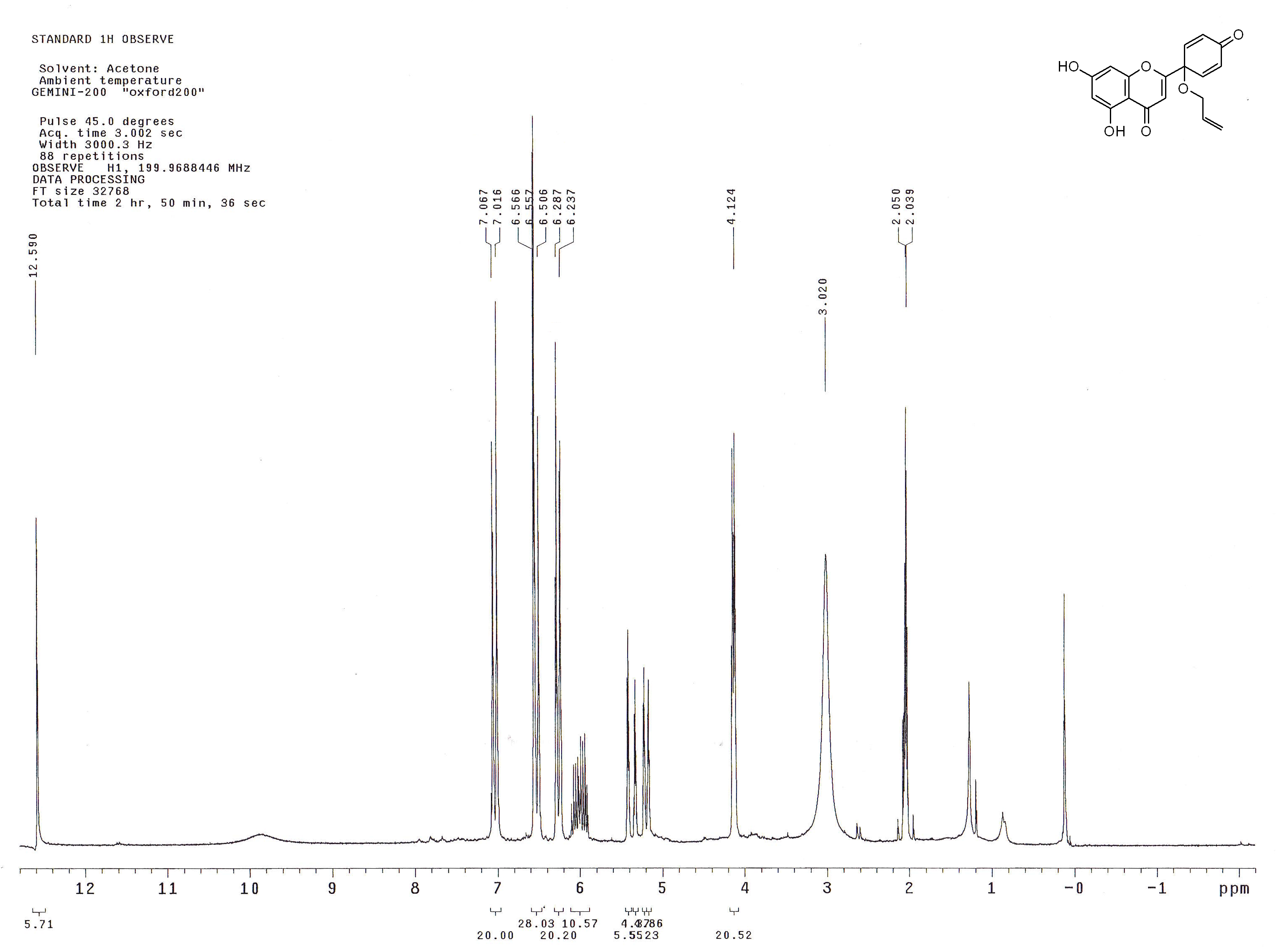

Supplement: Figure S38 — 200 MHz 1H NMR spectrum of compound 8 before crystallization. (TIF) [file pone.0023922.s038.tif]

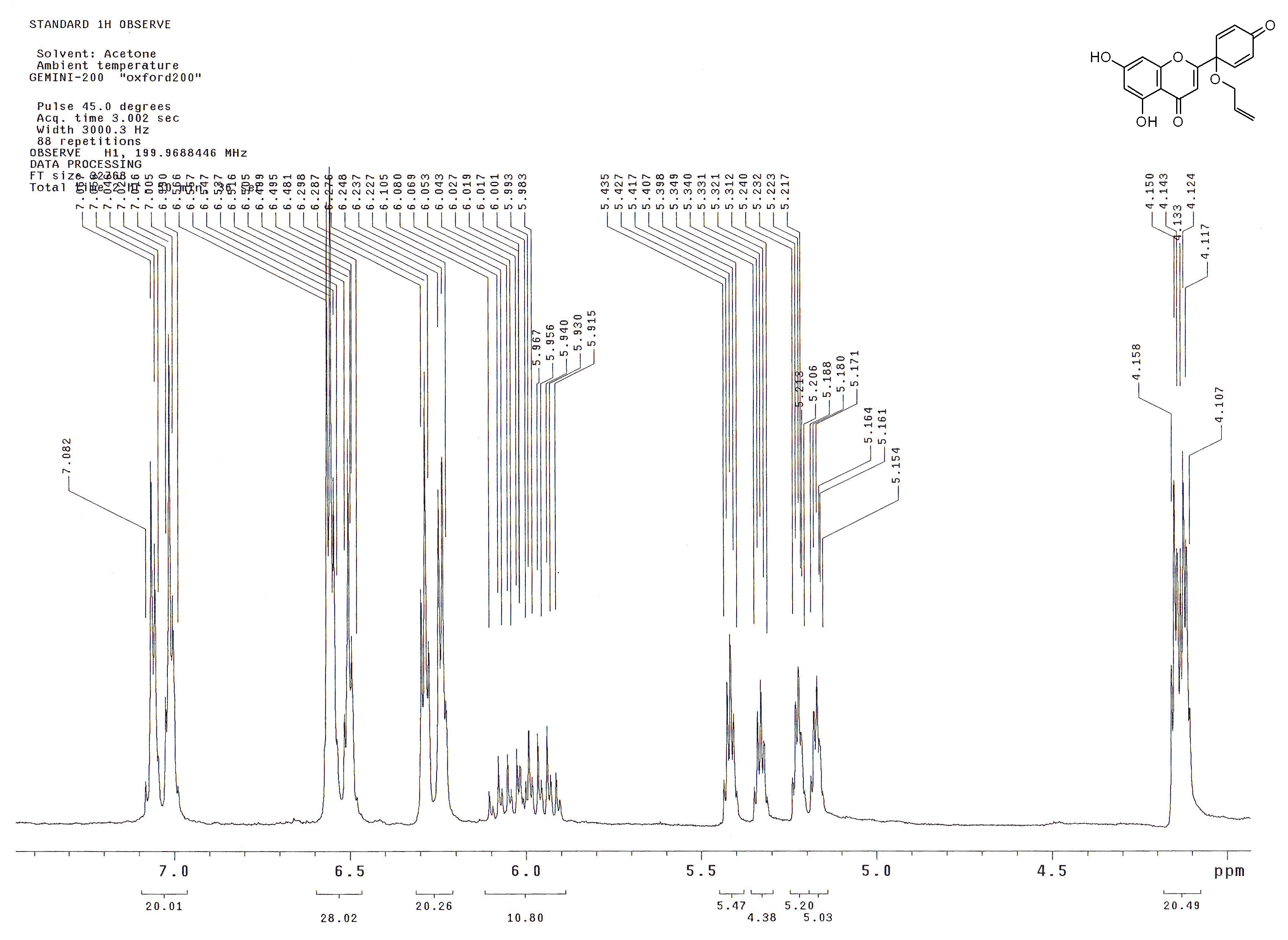

Supplement: Figure S39 — Zoom of 200 MHz 1H NMR spectrum of compound 8 before crystallization. (TIF) [file pone.0023922.s039.tif]

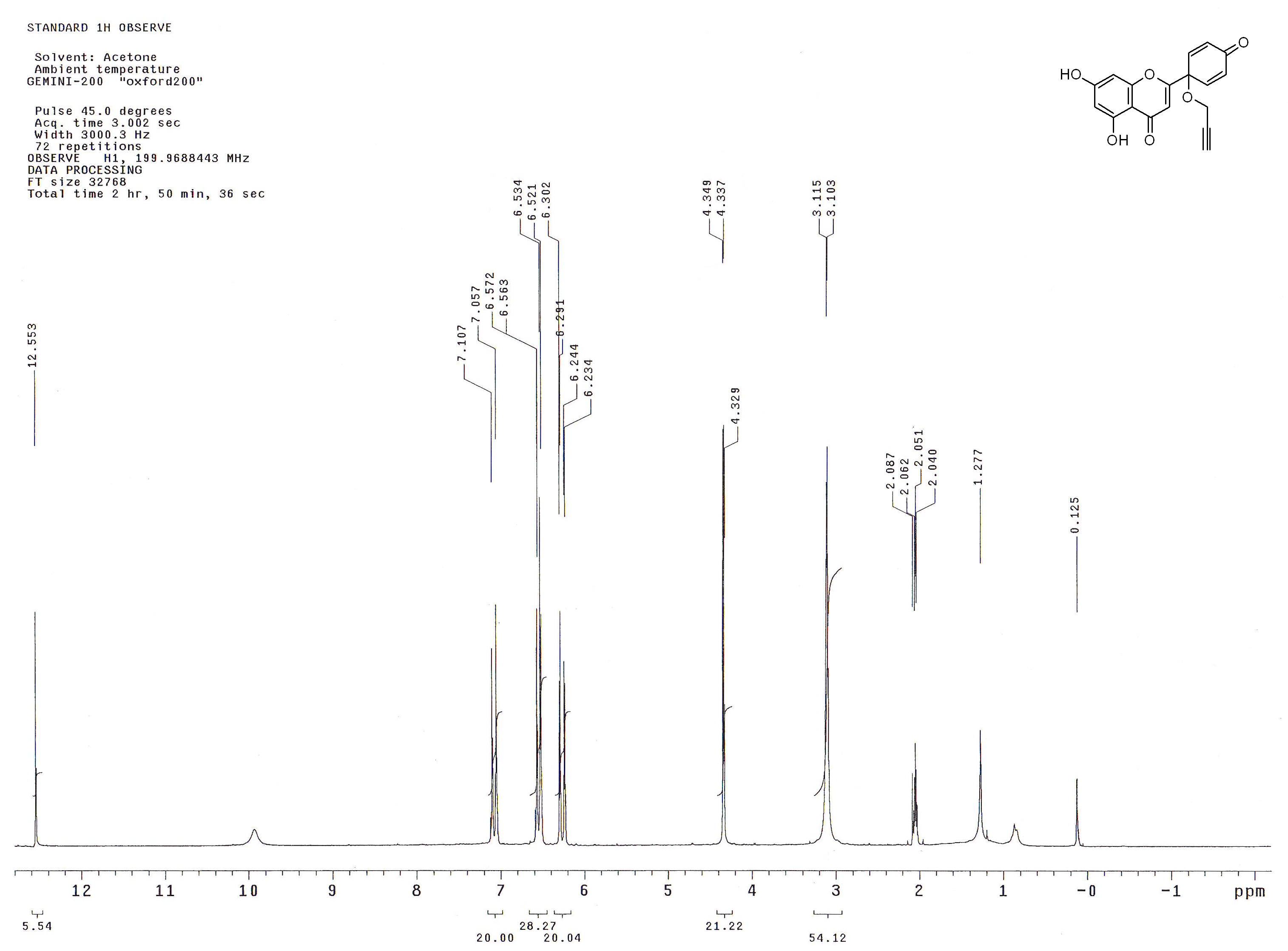

Supplement: Figure S40 — 200 MHz 1H NMR spectrum of compound 9 before crystallization. (TIF) [file pone.0023922.s040.tif]

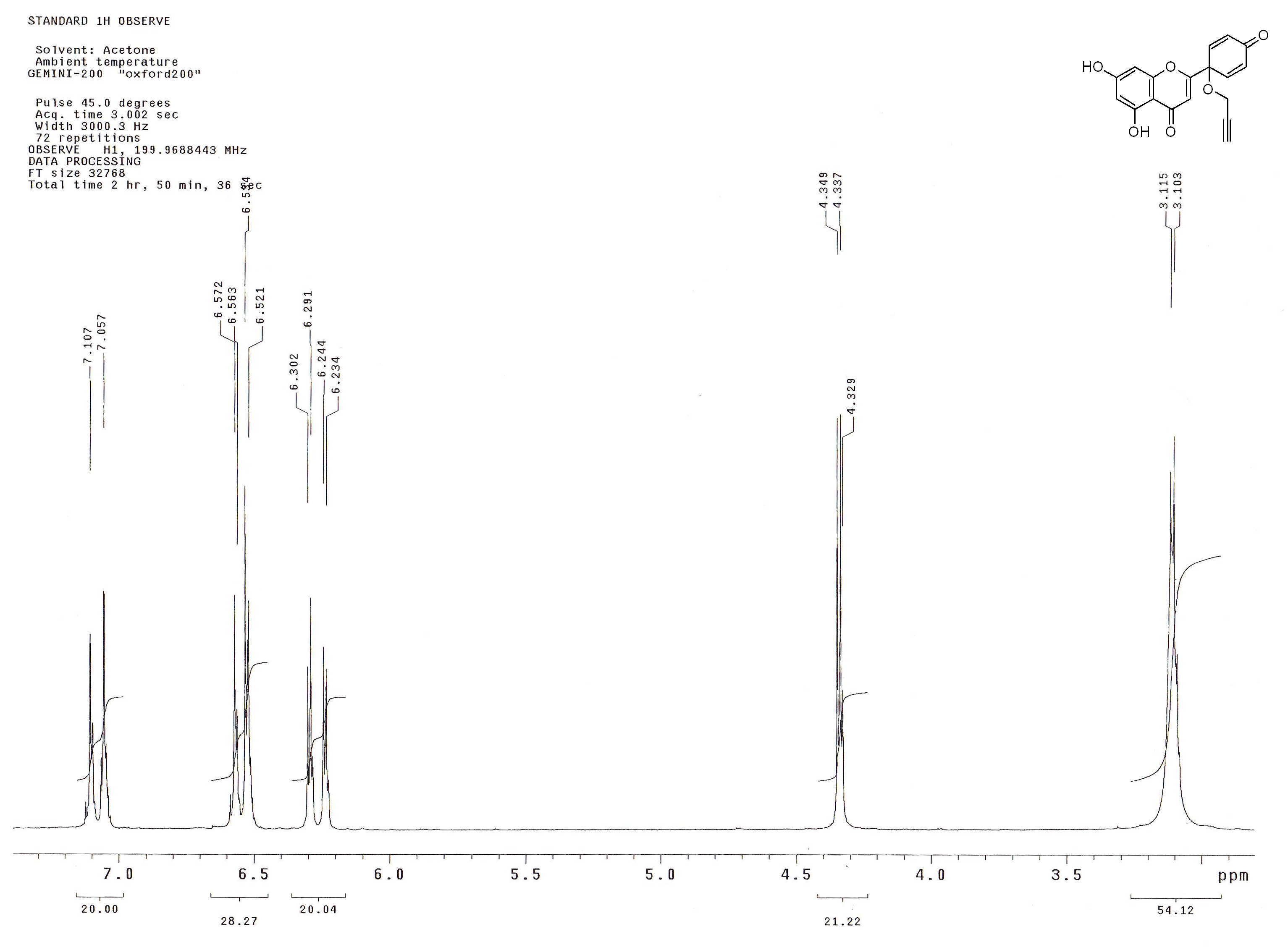

Supplement: Figure S41 — Zoom of 200 MHz 1H NMR spectrum of compound 9 before crystallization. (TIF) [file pone.0023922.s041.tif]

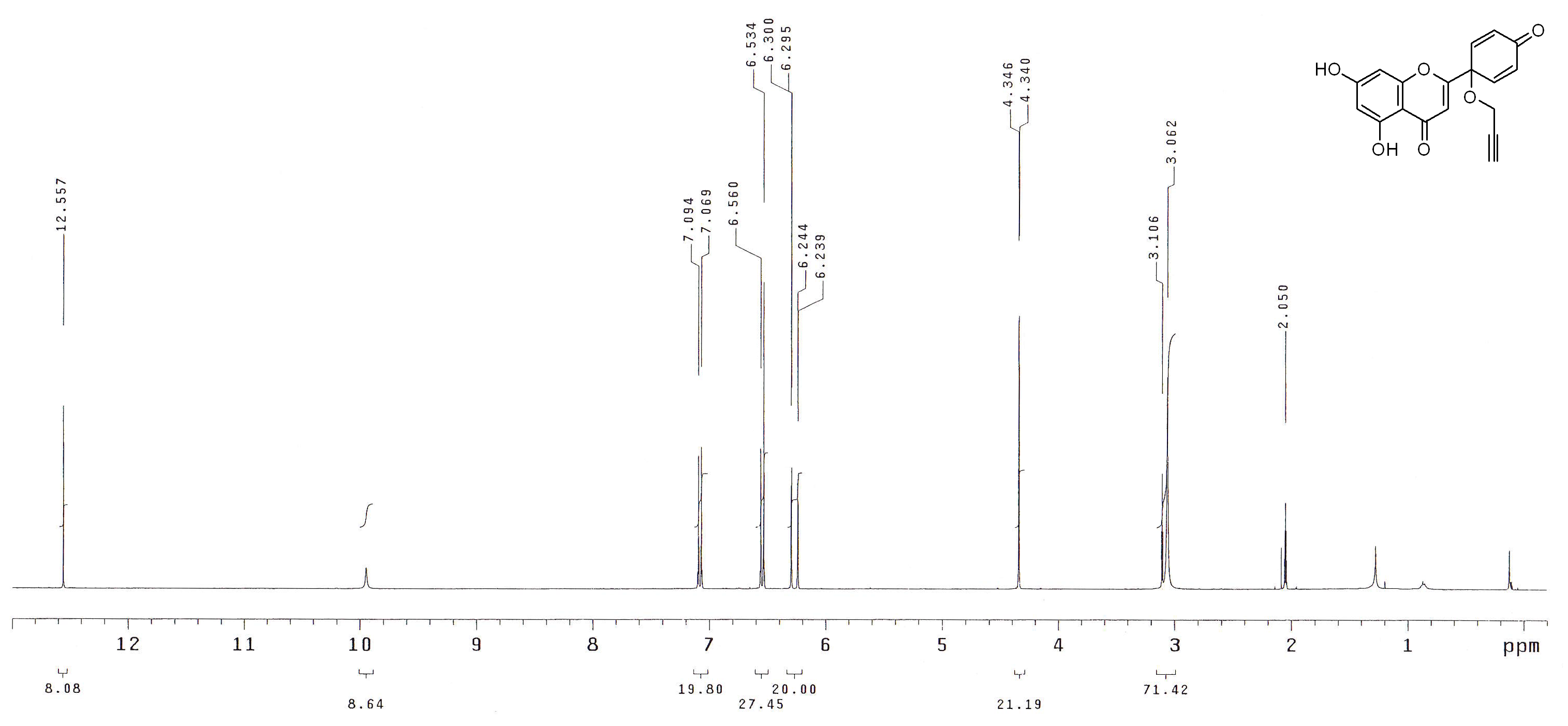

Supplement: Figure S42 — 400 MHz 1H NMR spectrum of compound 9 before crystallization. (TIF) [file pone.0023922.s042.tif]

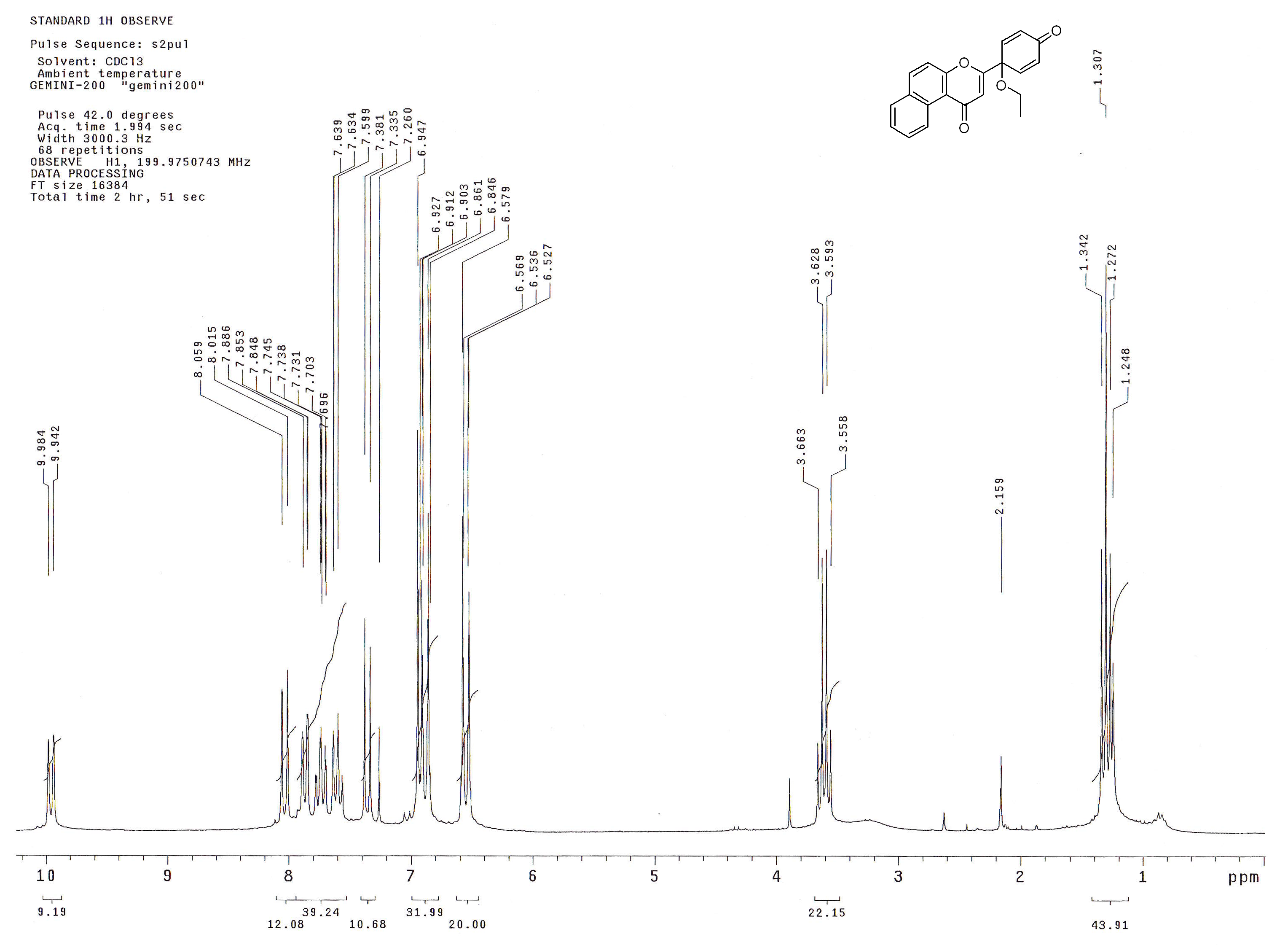

Supplement: Figure S43 — 200 MHz 1H NMR spectrum of compound 13 before crystallization. (TIF) [file pone.0023922.s043.tif]

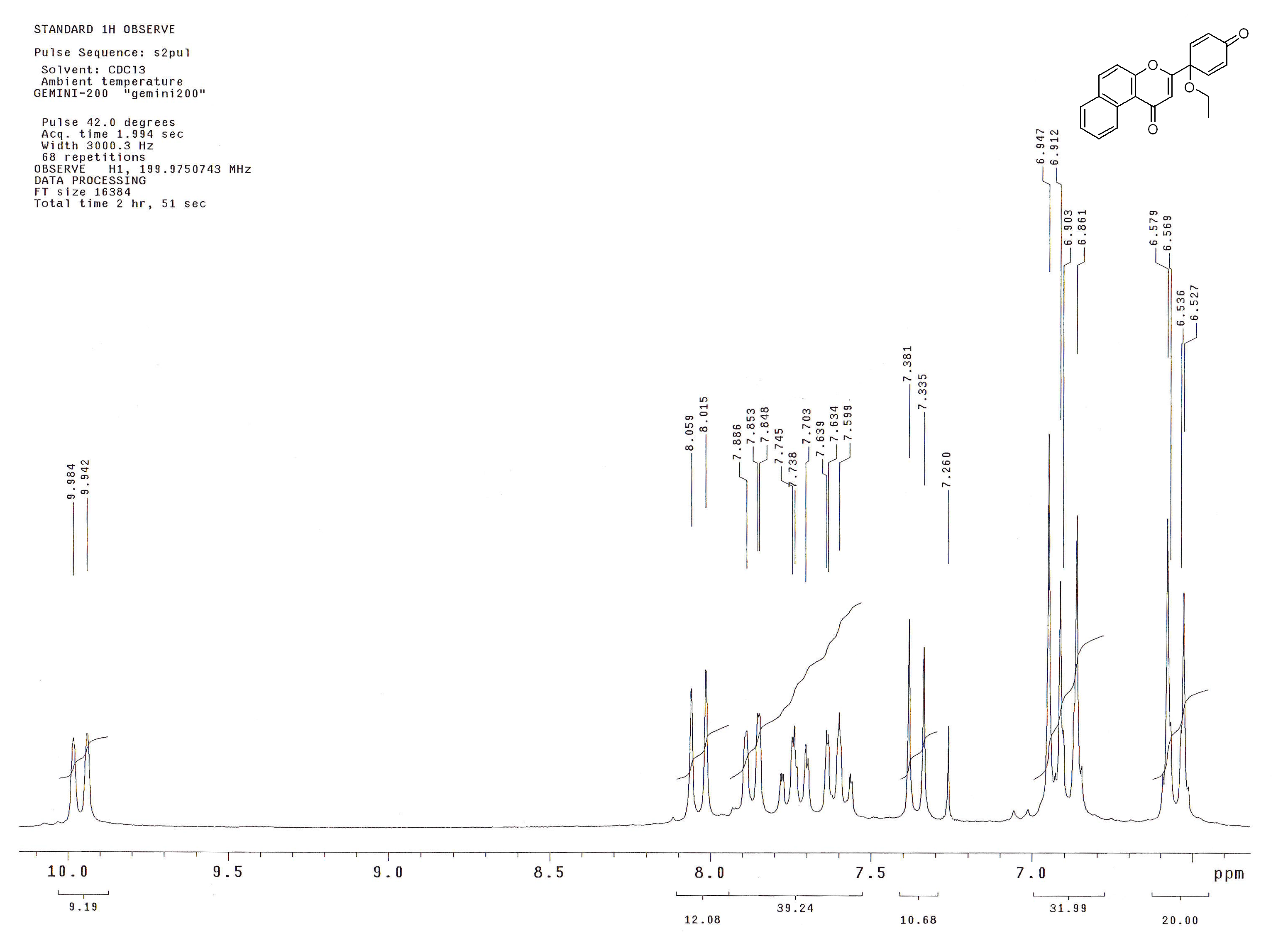

Supplement: Figure S44 — Zoom of 200 MHz 1H NMR spectrum of compound 13 before crystallization. (TIF) [file pone.0023922.s044.tif]

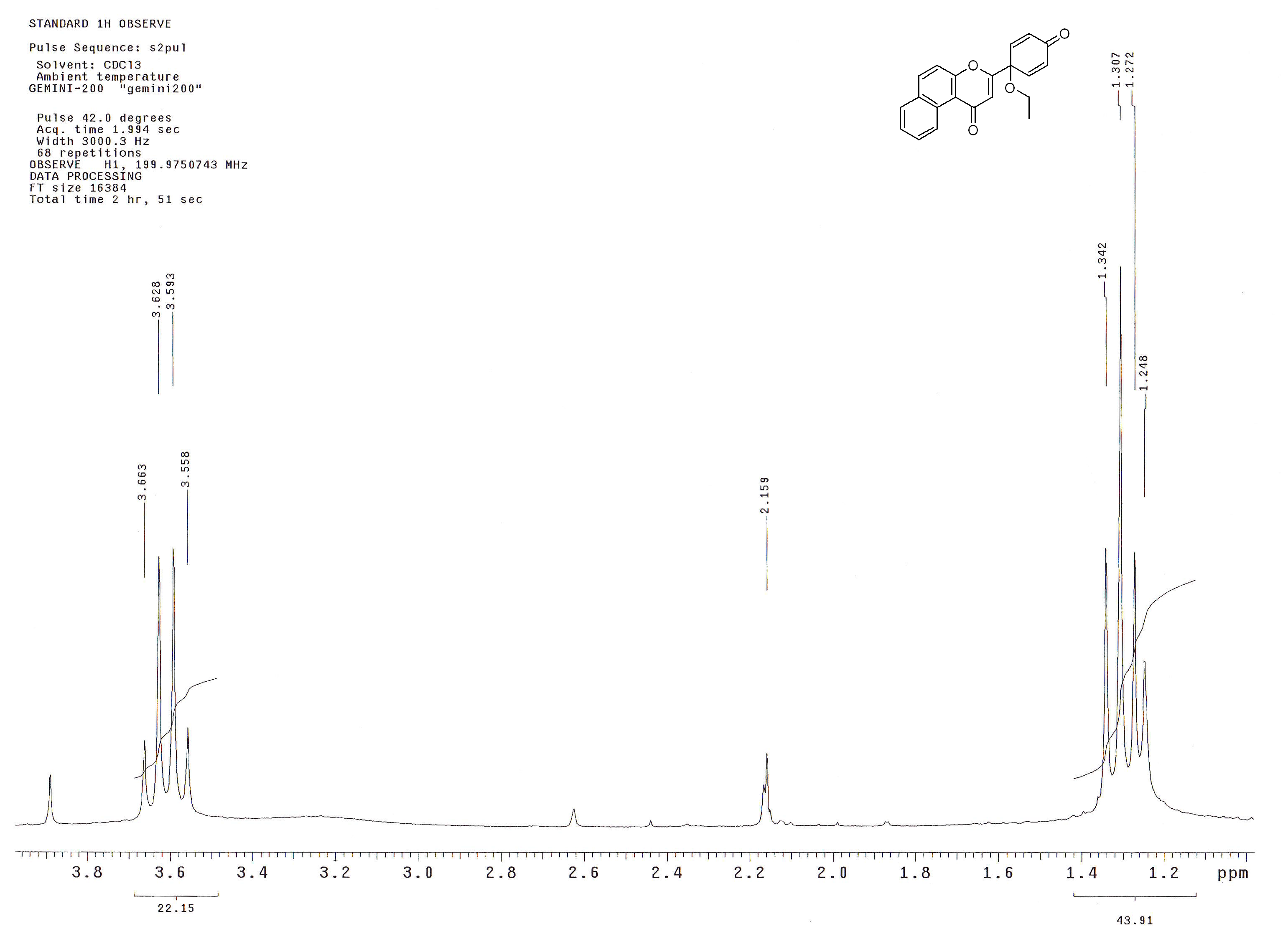

Supplement: Figure S45 — Zoom of 200 MHz 1H NMR spectrum of compound 13 before crystallization. (TIF) [file pone.0023922.s045.tif]

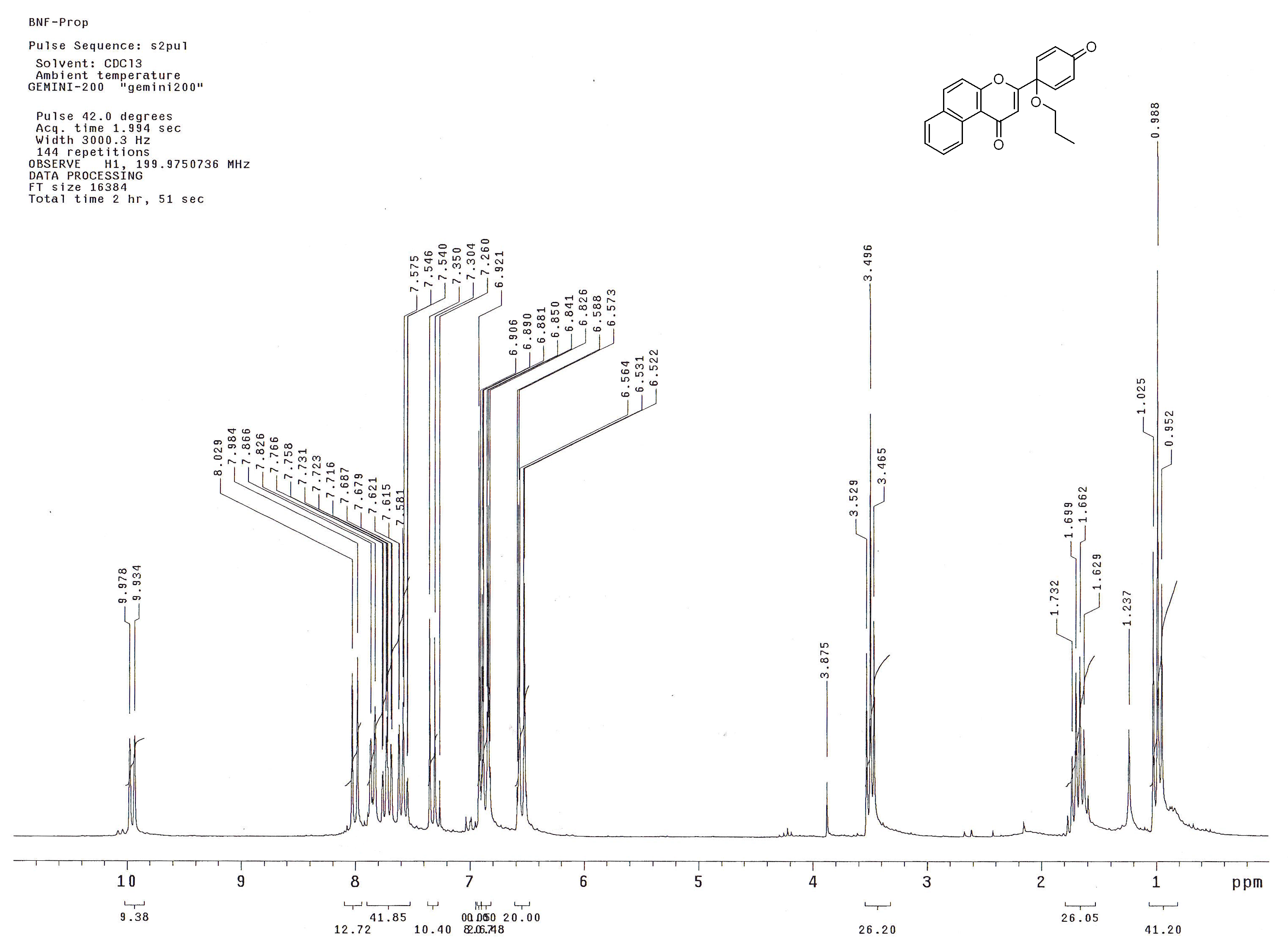

Supplement: Figure S46 — 200 MHz 1H NMR spectrum of compound 14 before crystallization. (TIF) [file pone.0023922.s046.tif]

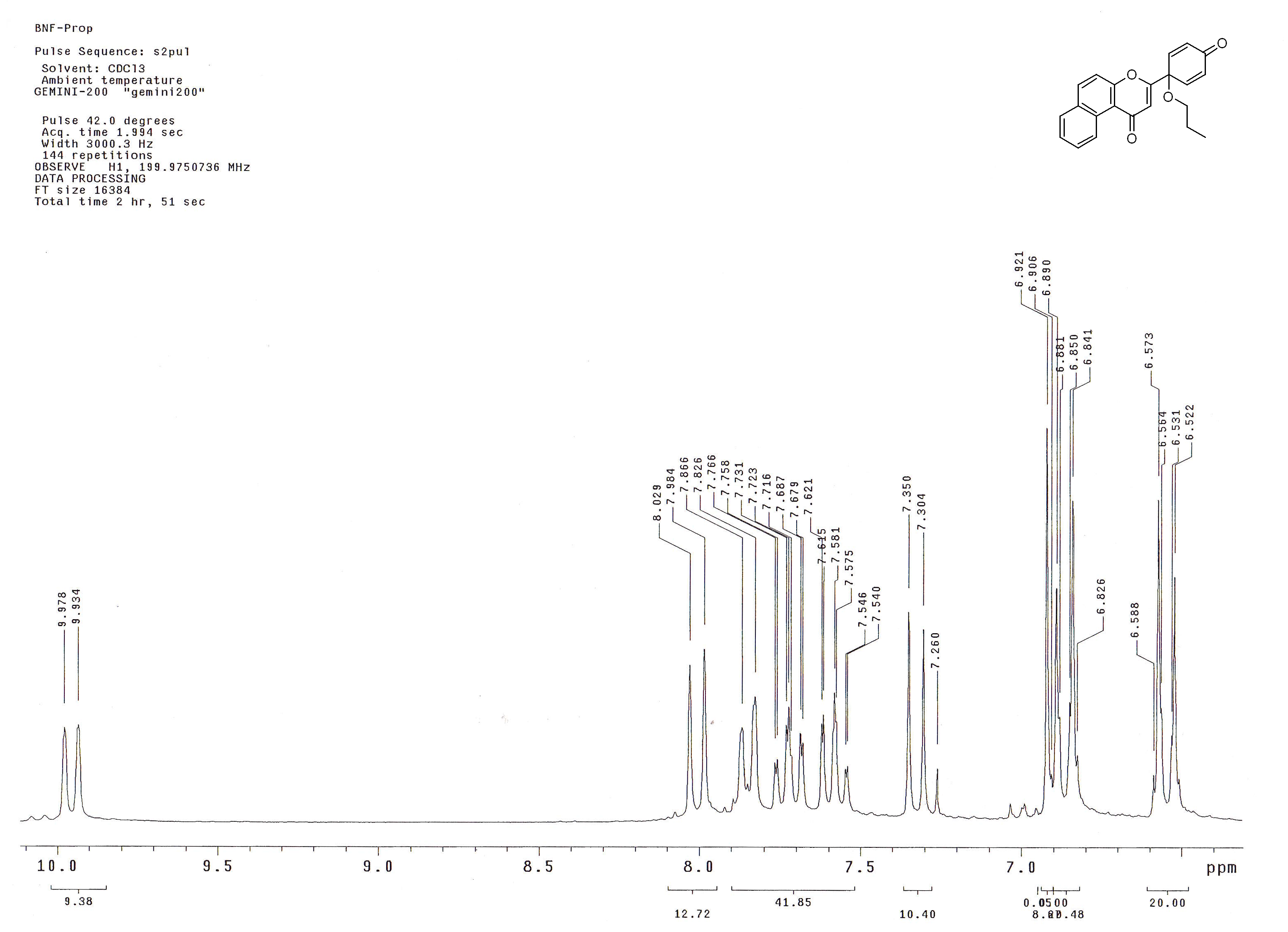

Supplement: Figure S47 — Zoom of 200 MHz 1H NMR spectrum of compound 14 before crystallization. (TIF) [file pone.0023922.s047.tif]

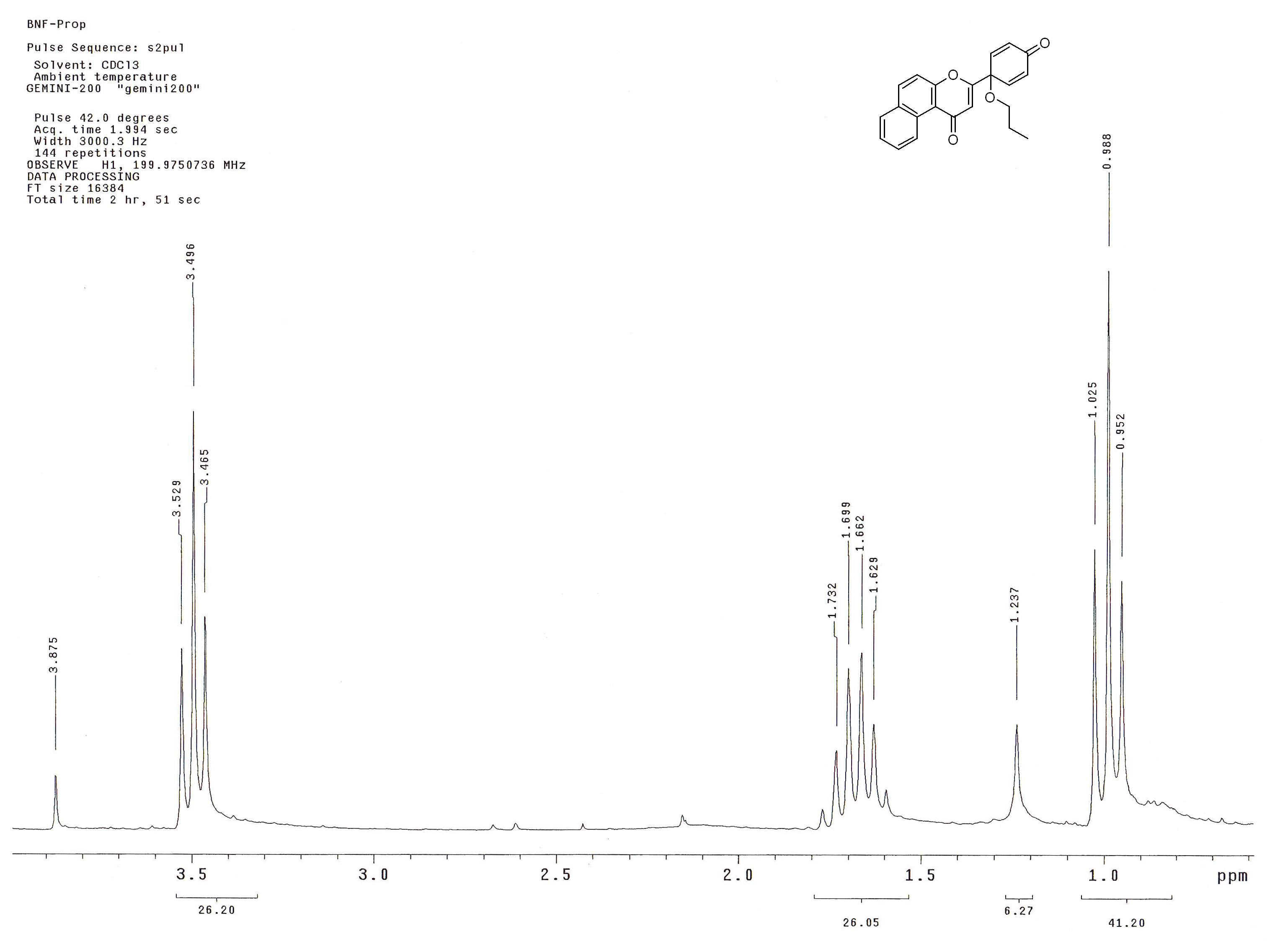

Supplement: Figure S48 — Zoom of 200 MHz 1H NMR spectrum of compound 14 before crystallization. (TIF) [file pone.0023922.s048.tif]

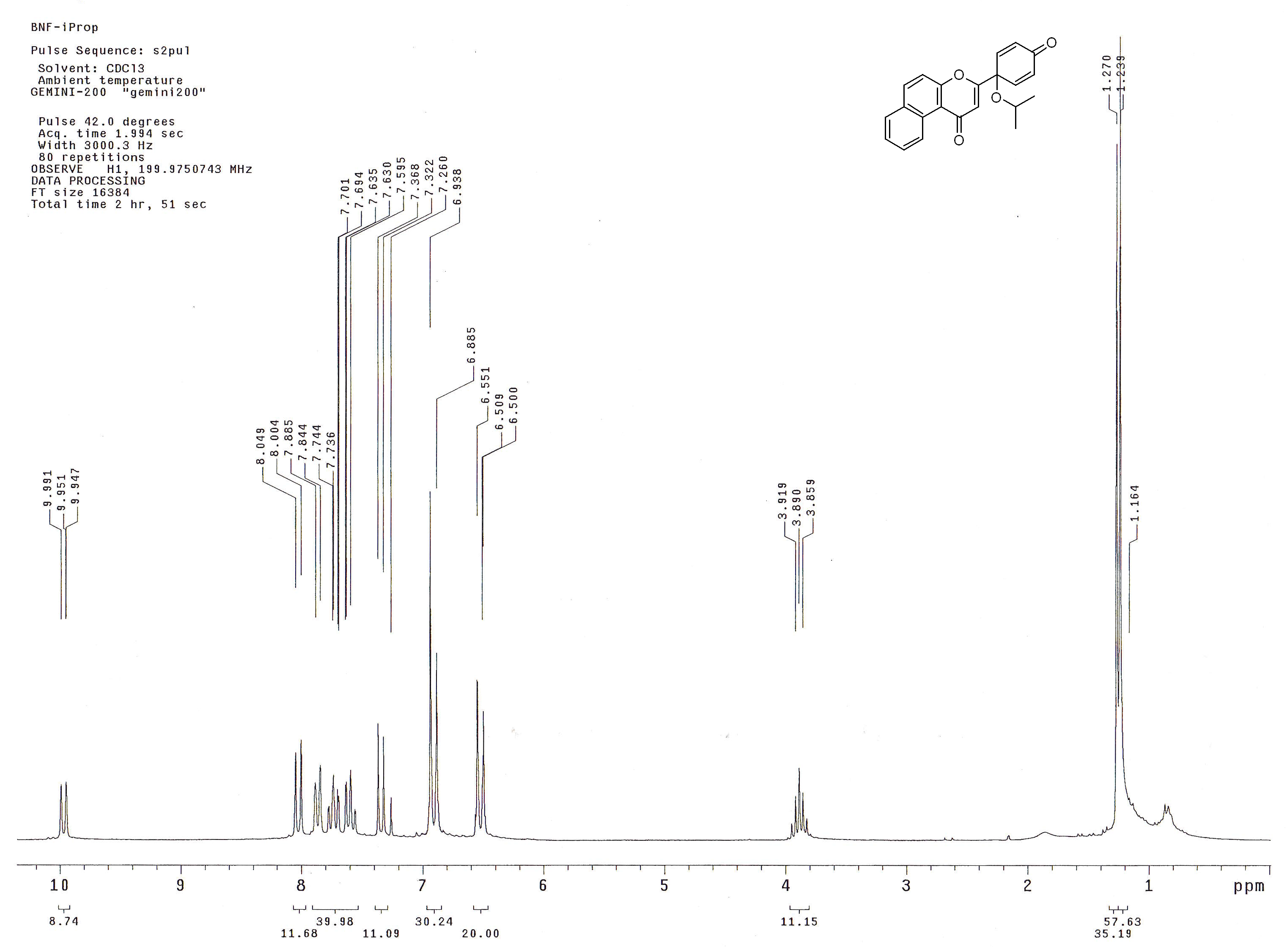

Supplement: Figure S49 — 200 MHz 1H NMR spectrum of compound 15 before crystallization. (TIF) [file pone.0023922.s049.tif]

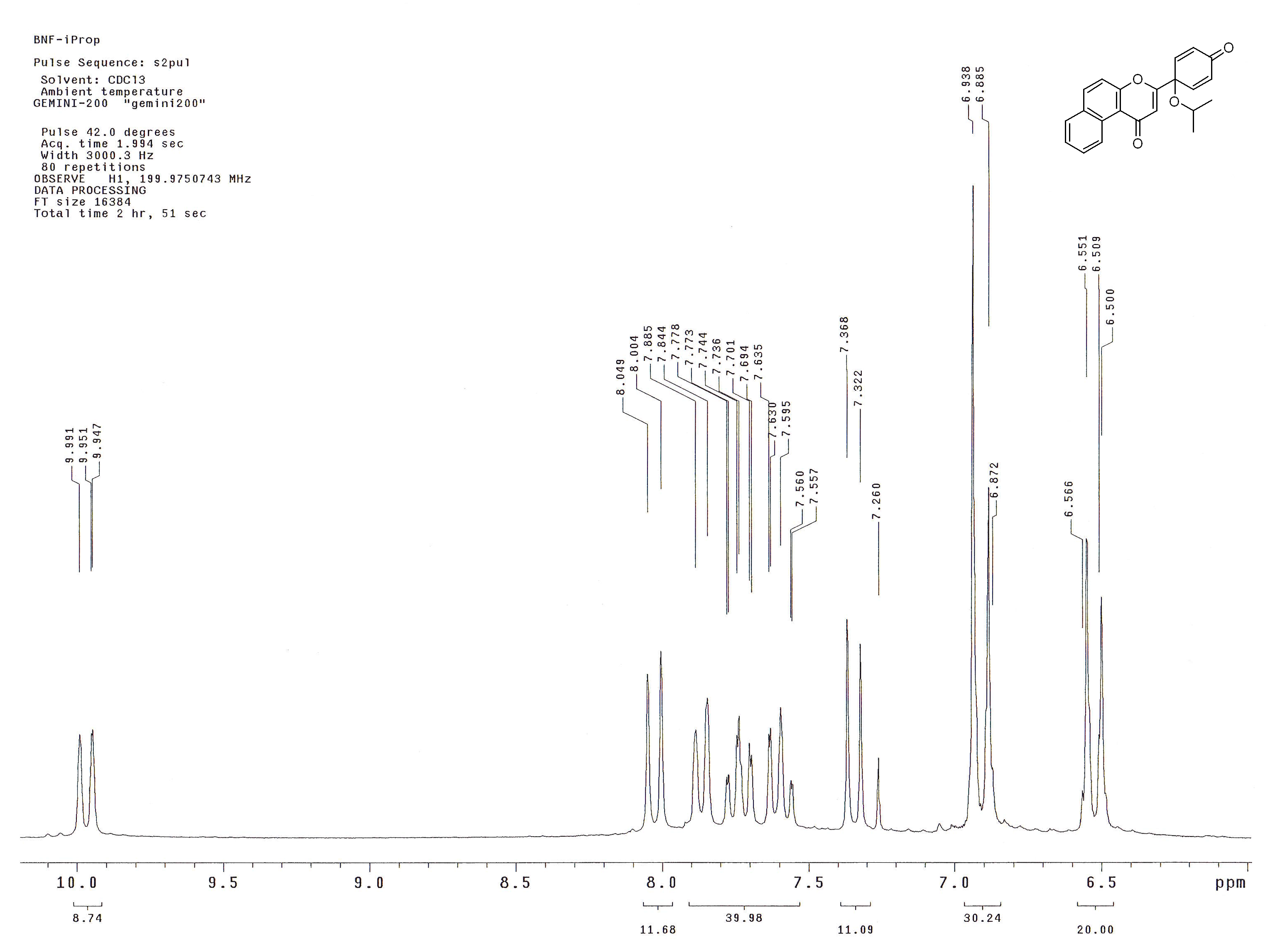

Supplement: Figure S50 — Zoom of 200 MHz 1H NMR spectrum of compound 15 before crystallization. (TIF) [file pone.0023922.s050.tif]

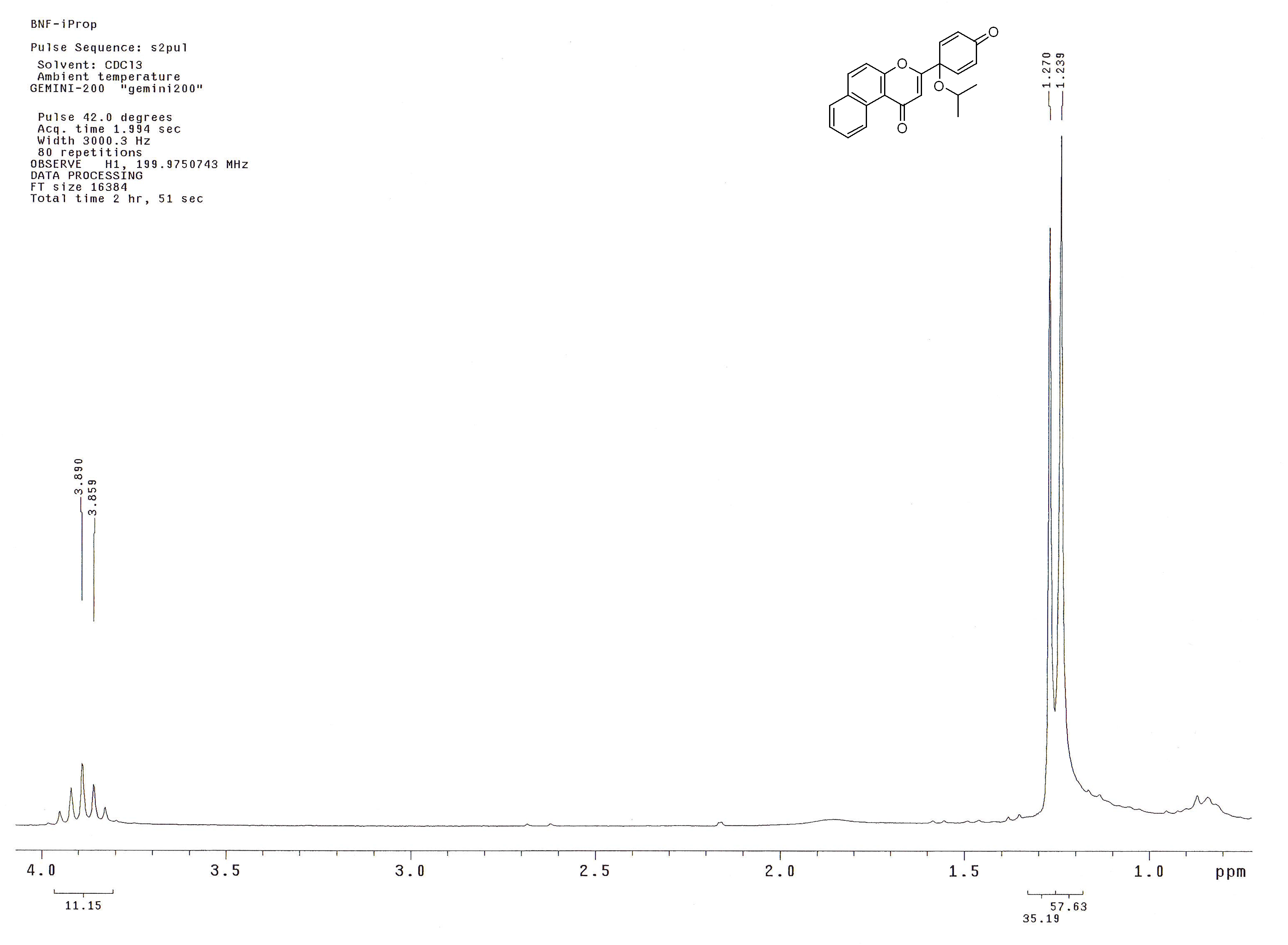

Supplement: Figure S51 — Zoom of 200 MHz 1H NMR spectrum of compound 15 before crystallization. (TIF) [file pone.0023922.s051.tif]

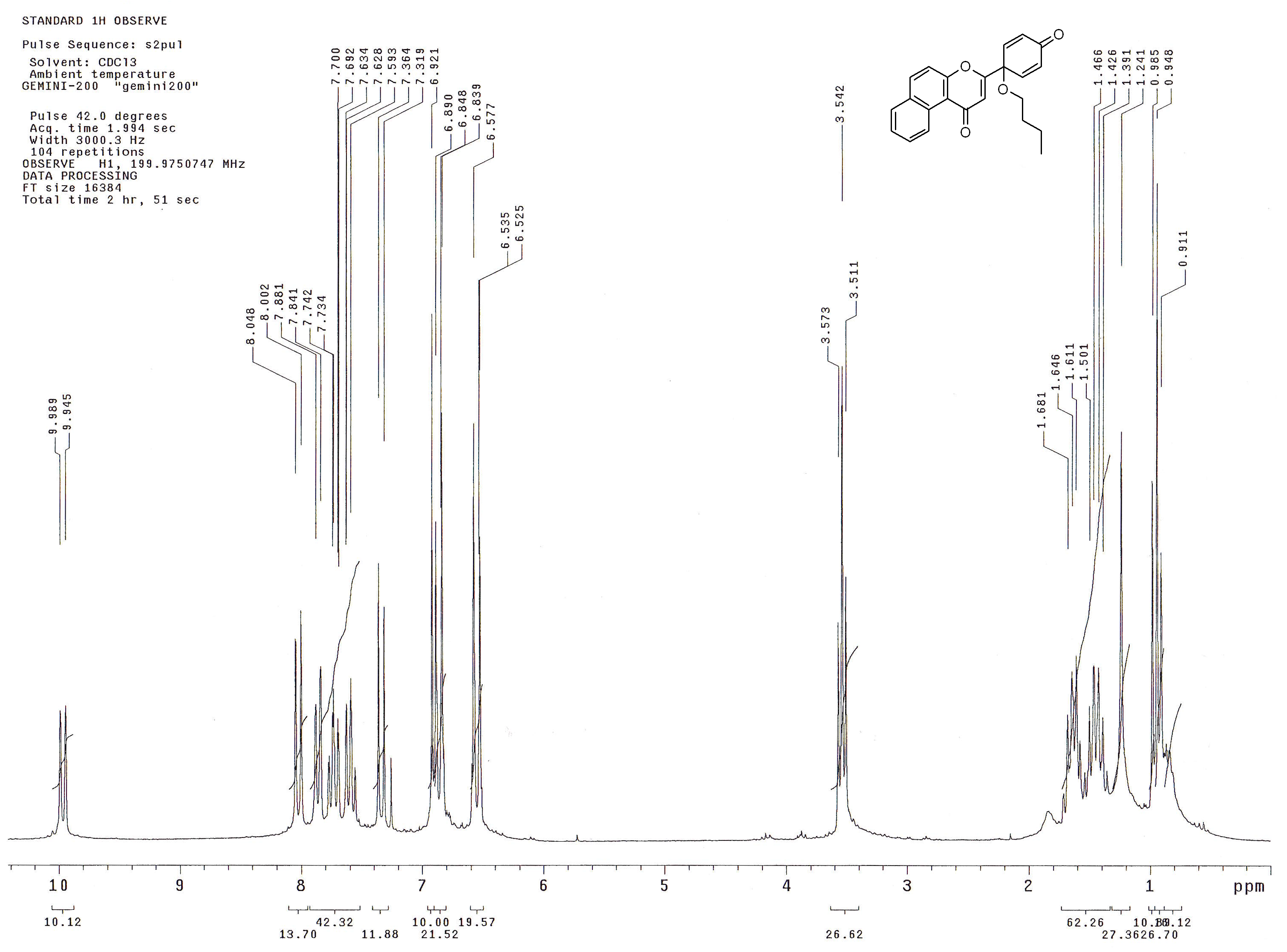

Supplement: Figure S52 — 200 MHz 1H NMR spectrum of compound 16 before crystallization. (TIF) [file pone.0023922.s052.tif]

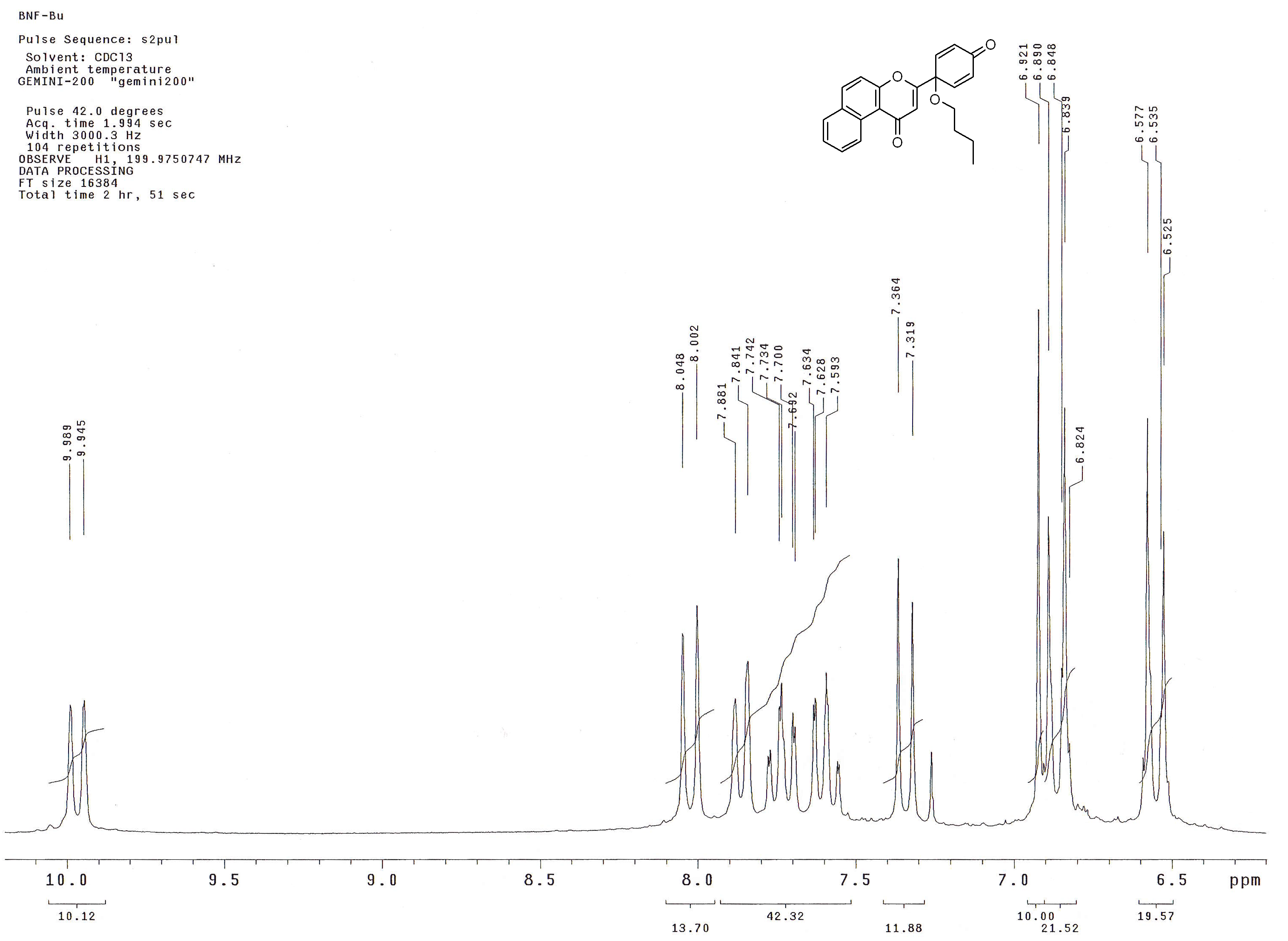

Supplement: Figure S53 — Zoom of 200 MHz 1H NMR spectrum of compound 16 before crystallization. (TIF) [file pone.0023922.s053.tif]

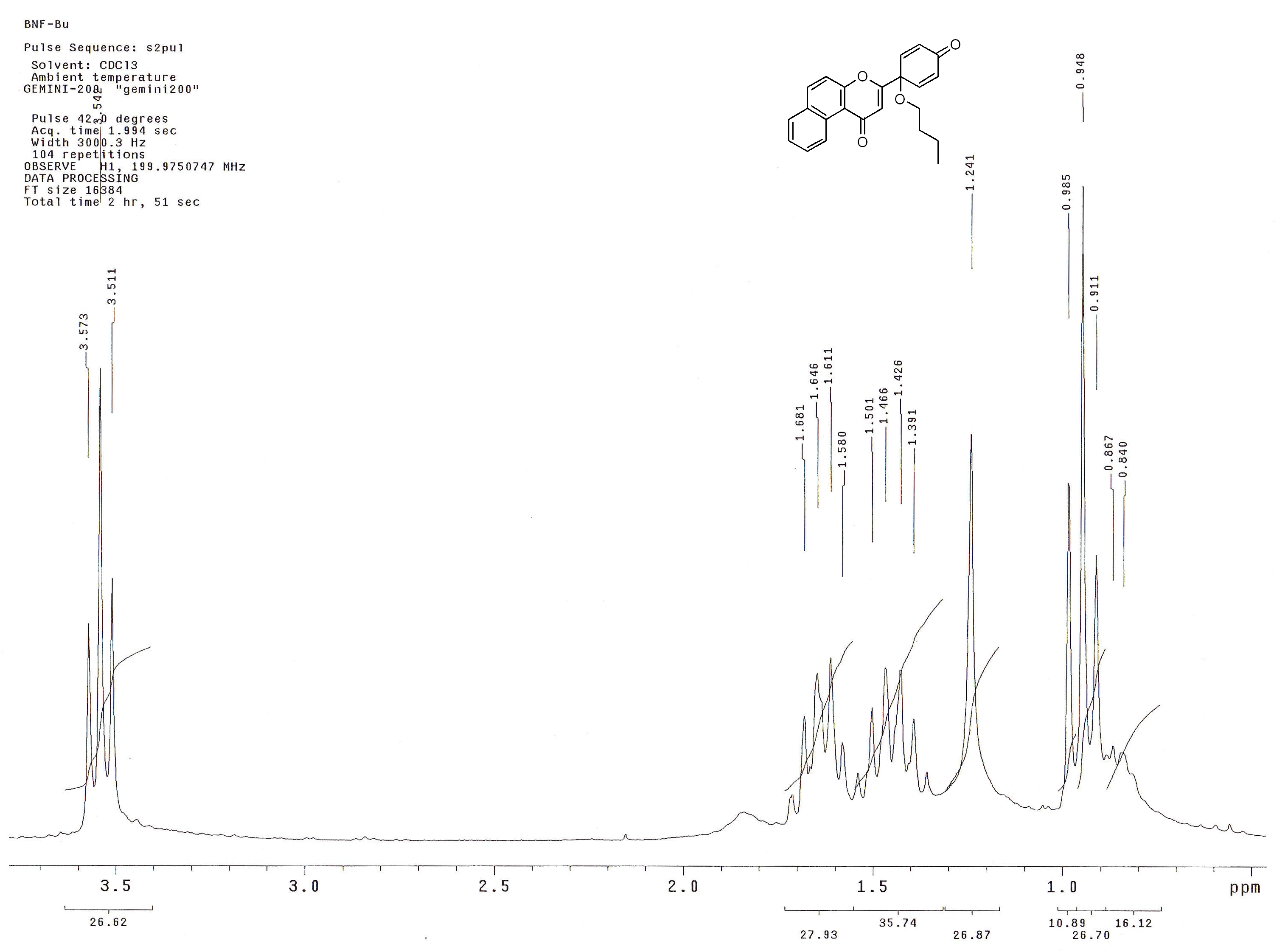

Supplement: Figure S54 — Zoom of 200 MHz 1H NMR spectrum of compound 16 before crystallization. (TIF) [file pone.0023922.s054.tif]

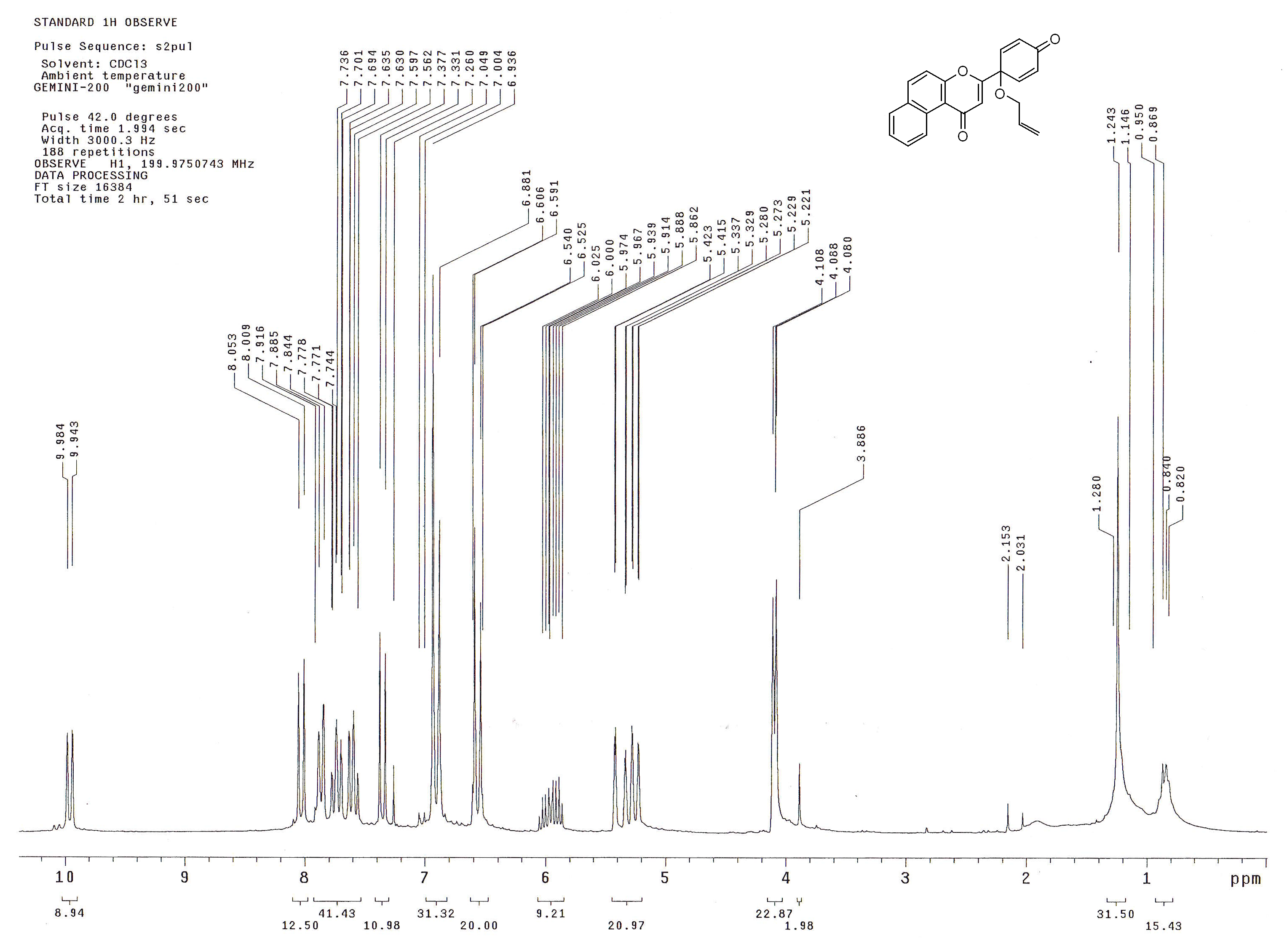

Supplement: Figure S55 — 200 MHz 1H NMR spectrum of compound 17 before crystallization. (TIF) [file pone.0023922.s055.tif]

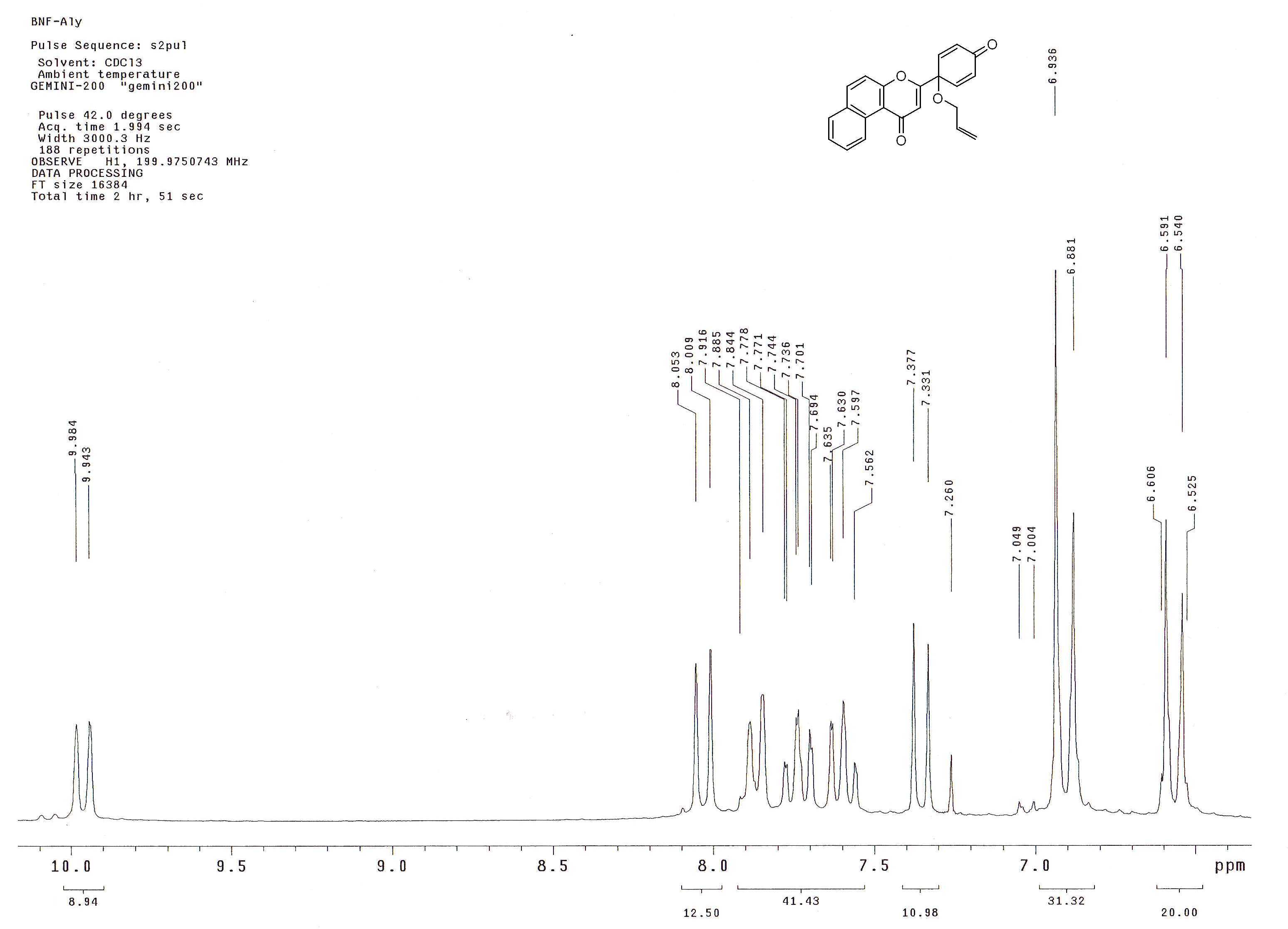

Supplement: Figure S56 — Zoom of 200 MHz 1H NMR spectrum of compound 17 before crystallization. (TIF) [file pone.0023922.s056.tif]

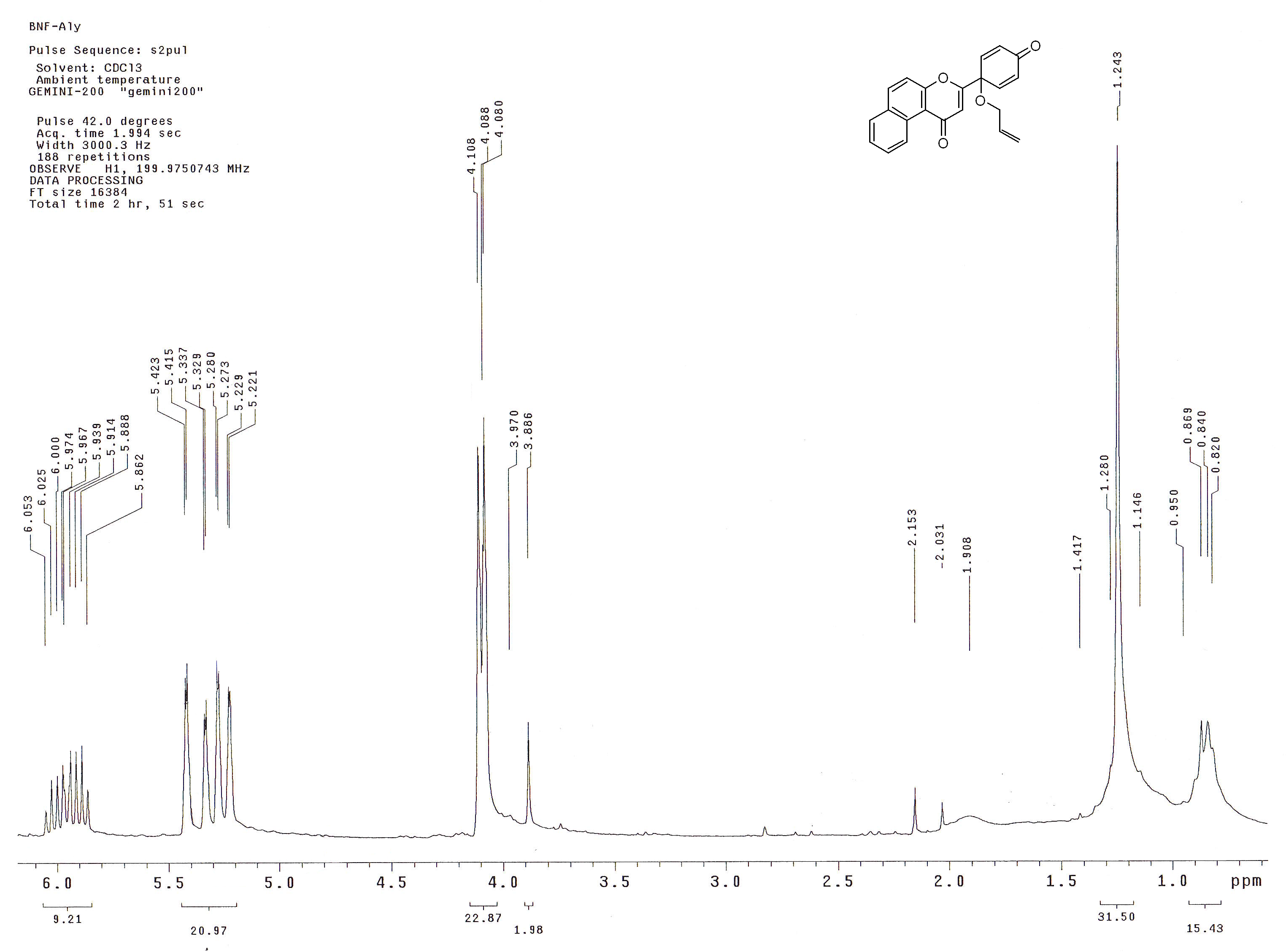

Supplement: Figure S57 — Zoom 200 MHz 1H NMR spectrum of compound 17 before crystallization. (TIF) [file pone.0023922.s057.tif]

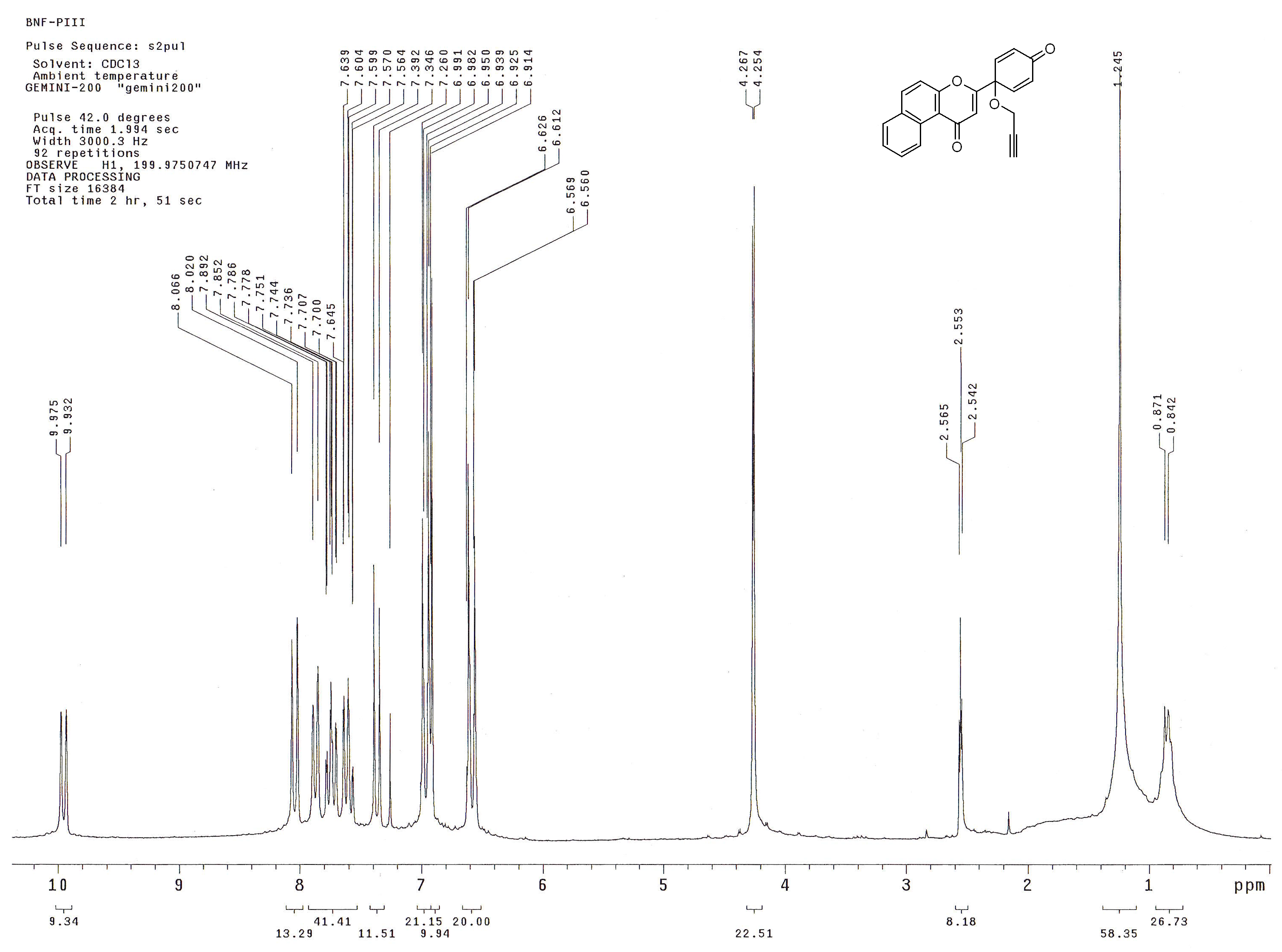

Supplement: Figure S58 — 200 MHz 1H NMR spectrum of compound 18 before crystallization. (TIF) [file pone.0023922.s058.tif]

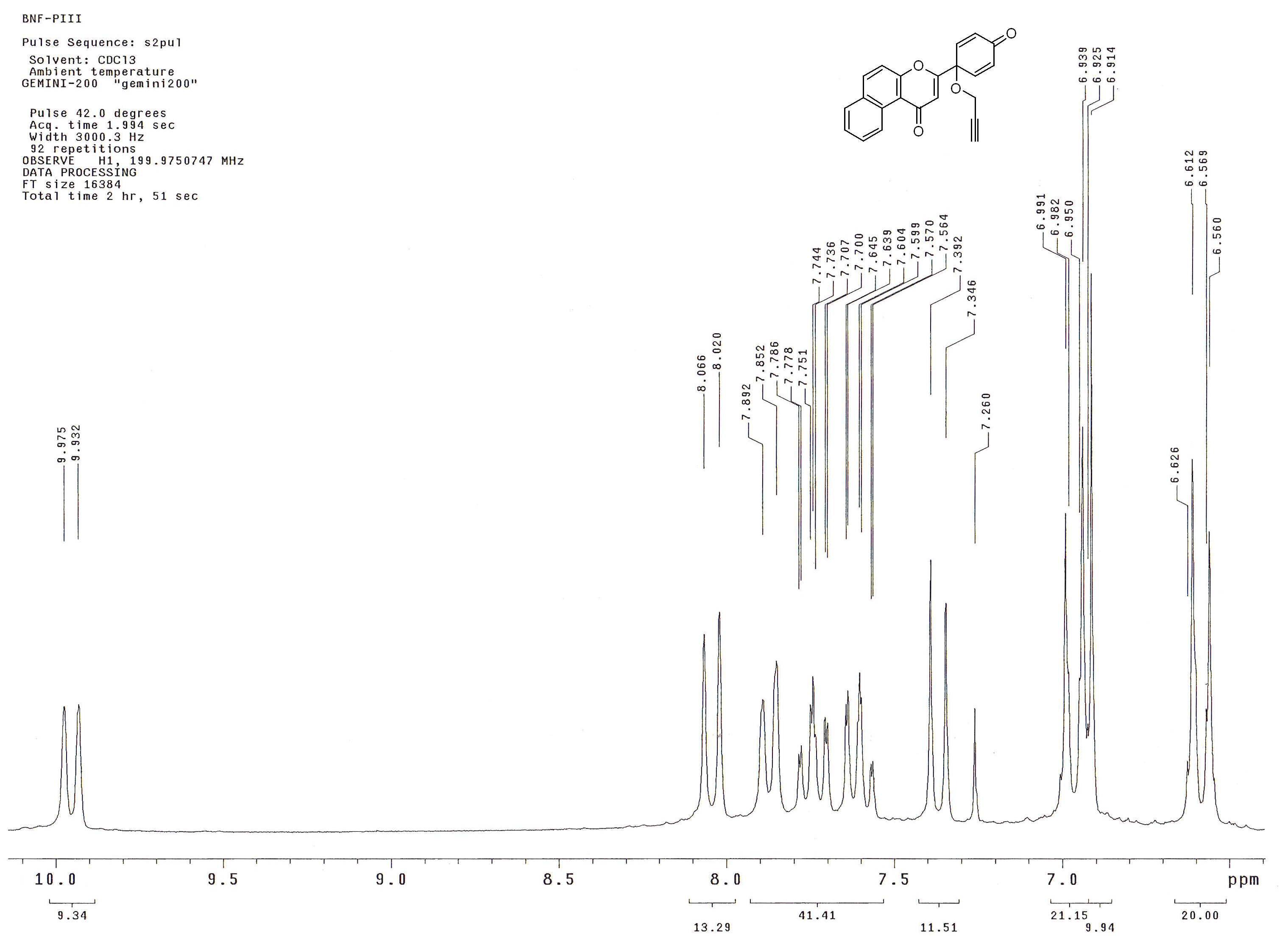

Supplement: Figure S59 — Zoom of 200 MHz 1H NMR spectrum of compound 18 before crystallization. (TIF) [file pone.0023922.s059.tif]

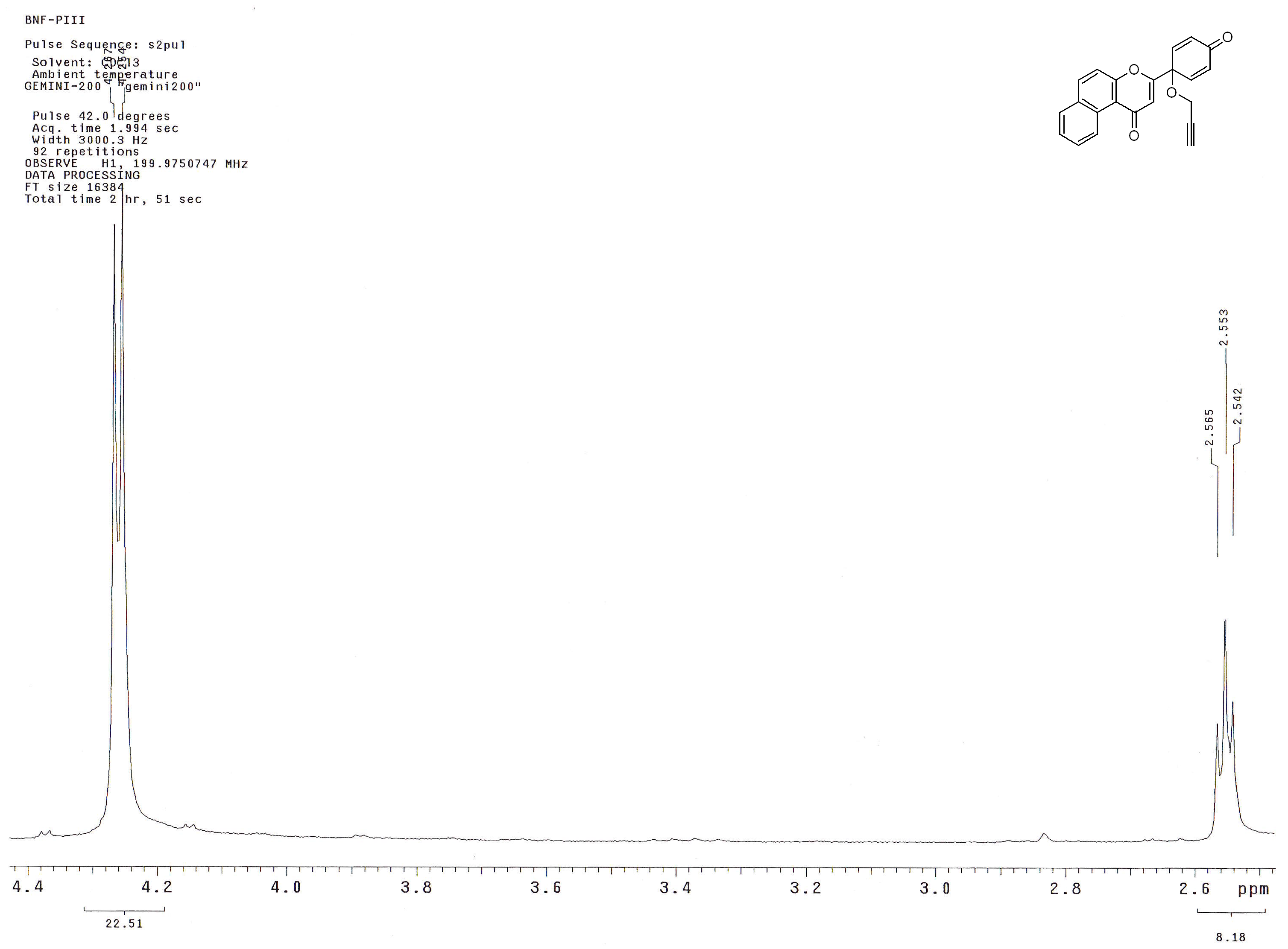

Supplement: Figure S60 — Zoom of 200 MHz 1H NMR spectrum of compound 18 before crystallization. (TIF) [file pone.0023922.s060.tif]
